# Vaccination accelerates the race of influenza virus against collective immunity

**DOI:** 10.1371/journal.ppat.1014311

**Published:** 2026-10-12

**Authors:** Igor M. Rouzine

**Affiliations:** Sechenov Institute of Evolutionary Physiology and Biochemistry RAS, St Petersburg, Russia; City University of Hong Kong, HONG KONG

## Abstract

Respiratory viruses persist in a population, because their rapid evolution allows them to escape recognition by antibodies in previously-infected individuals. To optimize parameters of an effective vaccine, an epidemiological model of a population infected with an evolving virus is coupled to a model of multi-locus evolution. Model parameters are informed from influenza virus data. The cases of overlapping and non-overlapping effect of consecutive vaccinations are considered. The results predict that vaccination will accelerate viral evolution and may even increase infection incidence, unless one of two scenarios takes place: either antibodies elicited by vaccine are more broadly neutralizing than antibodies to natural infection, or the vaccine vector lies slightly ahead of the dominant virus variant in the evolutionary trajectory. These results suggest that the probabilistic prognosis of virus evolution represents a promising strategy of vaccine improvement complementing the search for broadly-neutralizing vaccines.

## Author summary

Some viruses persist in a population, because their rapid evolution allows them to escape recognition by antibodies in previously-infected individuals. Developing effective vaccines against these viruses poses a challenge. To predict parameters of an effective vaccine, a mathematical model of population infected with various strains of evolving virus and keeping immune memory cells left by past viral strains is developed. The model is based on matching the standard epidemiological approach to multi-site evolution models and is informed from available data on influenza virus. Results demonstrate that vaccination will accelerate viral evolution and may even increase infection incidence, unless one of two following conditions takes place. Either the immune response to vaccine is more broadly neutralizing than that the immune response to infection, or the vaccine vector is slightly ahead of the virus on evolutionary trajectory. Given the difficulties with developing broadly-neutralizing vaccines, the “forward-neutralizing” vaccines offer a promising alternative. The approach relies on the accurate probabilistic prognosis of virus evolution from computational models and genomic data.

## Introduction

Vaccination is one of the greatest inventions that saves annually millions of lives. Effective and safe vaccines have been developed against such dangerous pathogens as smallpox, plague, rubella, mumps, measles, tuberculosis, polio, tetanus, rabies, hepatitis B, Ebola, yellow fever. However, there is a small group of pathogens constantly mutating to escape the immune response and persist in a population. The efficacy of vaccines against such pathogens remains limited, both for individual protection and for reducing their spread in a population.

Human immunodeficiency virus, hepatitis C virus, influenza virus, and coronaviruses belong to this exceptional group. They belong to four different genera and propagate by different routes. Their pathogenesis varies as well. HIV and HCV persist in an organism, while coronaviruses and influenza are cleared in several days or weeks. What all these different viruses have in common is the rapid evolution of the antibody–binding region used as a means of virus survival in a population. For SARS-CoV-2, the substitution rate in a population varies between different variants in the range of $(\text{0}.\text{7}-\text{1}.\text{1})\times {\text{1}\text{0}}^{-\text{3}}$ per year per base [1] for the entire genome and $(\text{5}-\text{6})\times {\text{1}\text{0}}^{-\text{3}}$ for envelope protein Spike [2–5]. The last substitution rate is three-fold higher than in the envelope proteins of influenza virus [6] and hepatitis C virus [7], yielding only to HIV envelope protein [8], $(\text{1}.\text{5}-\text{2})\times {\text{1}\text{0}}^{-\text{2}}$.

Rapid evolution in viral envelope proteins has multiple reasons. Firstly, each of them has a receptor-binding motif responsible for the entrance into a host cell, and its evolution may lead to an enhanced reproduction number [9–12]. Secondly, a receptor protein contains B cell epitopes, regions involved in the binding by neutralizing antibodies. Mutations in these regions decrease the binding energy and, hence, the viral recognition by the immune system [5,13,14]. These highly variable epitope regions have evolved to have low biochemical constraints on mutation, i.e., a low cost of mutation to viral fitness (reproduction number, transmission rate). In contrast, the receptor binding site has an important function and is very conserved, because it is located in a cavity between the variable regions to prevent its antibody binding [6,15–17].

Due to constant mutation in regions responsible for the neutralization by antibodies secreted by the memory B cells, the cited viruses persist in a population. Such adaptation of a species in the face of a co-evolving opposing species is termed “The Red Queen effect”. The process of antigenic escape was investigated in detail in genomic and immunological studies [18–24] and is the reason why influenza virus persists in the human population [6,25–32]. Consequently, the effectiveness of vaccination against influenza is highly variable and sometimes even negative, depending on the match of the vaccine vector to current circulating strains [33–35]. Perpetual antigenic escape is also observed in SARS-CoV-2 and seasonal human coronaviruses [2–5,36]. For the Omicron variants, the effectiveness of EU-licensed COVID-19 vaccines in preventing SARS-CoV-2 infection is only 34−66% after booster vaccination [37] and experiences further decline within 3–6 months [37].

Different viruses use different strategy of survival, including latency and interference with the immune system. By contrast to influenza and coronaviruses, HIV and HCV also directly interfere with the immune system [38–40]. The combined effect of mutation and interference renders the existing vaccines against HIV and HCV completely ineffective [40,41].

While it is clear that the virus evolution in antibody epitopes lowers vaccine effectiveness, the opposite effect of vaccination on the virus evolution remains a subject of controversy. Some studies argued that, because the virus is poorly transmitted in the vaccinated population, the reduction in the effective reproduction number by vaccination will also slow down the evolution of epitopes and suppress infection incidence [42–44]. However, these studies are all based on one-locus models of evolution that do not capture evolutionary dynamics properly.

As it is known in population genetics since 1930s, the effects of genetic linkage arising from the common ancestry of different loci including “clonal interference” and “genetic background effect”, alter all evolutionary properties of a population including the speed of adaptation [45,46], the role of deleterious mutations [46,47], and the shape of phylogenetic trees [48–50]. The larger the number of evolving loci, the stronger linkage effects [46,51]. To take into account the strong linkage effects for the mentioned viruses, one must resort to more complex, multi-locus models of evolution combined with epidemiological models [26,30–32]. The use of multi-locus models allows to hypothesize that vaccine might actually accelerate viral evolution due to the increased positive selection pressure for epitope mutations [52].

The present work is designed to shed light on this complex question. Previous epidemiological models coupled to multi-locus models considered only the immunity acquired due to natural infection [26,30–32]. In another work [53,54], both natural and vaccine immunity were included, but virus evolution was not considered. Here we include both types of immunity and multi-locus evolution. A virus variant-structured epidemiological model coupled to the multi-locus evolution models [25,30] is generalized to include the effect of vaccination.

As demonstrated below, vaccination exerts two opposing effects on virus evolution, as follows. Deceleration due to the decrease in reproduction number is opposed by acceleration due to the viral escape from additional vaccine immunity. The net effect of vaccine on the substitution rate and infection incidence is evaluated analytically, as a function of vaccine parameters, parameters of the immune system, and basic reproduction number.

The evolution rate, the incidence, and the effective reproduction number are calculated analytically, as a function of virological and immunological parameters, using the mapping of an epidemiological SIR model onto the analytic models of asexual multi-locus evolution. The focus is on parameter values pertaining to influenza virus strain A H3N2.

## Model and methods

### Model

The model has two levels, epidemiological and evolutionary, as described below. The epidemiological block is used to calculate the average selection coefficient created by the immunity of population. The average selection coefficient is plugged, as an input parameter, into a multi-locus model of evolution [55,56].

### Epidemiological model

To account for the immunity to infection, the model classifies individuals according to virus variants they have been infected with in the past or are infected currently. Different virus variants are labeled by antigenic coordinate x representing the antigenic distance between a virus variant and the reference variant (1). Antigenic distance is measured in amino-acid substitutions. Unlike in Hamming genetic distance, where aminoacid substitutions are simply counted, in antigenic distance they are weighted with their relative effects on the viral transmission rate, which vary broadly between sites (see below). The value of antigenic distance is normalized to be equal to the genetic distance when averaged over a large number of substitutions.

The antibody–binding segments of hemagglutinin protein, the main target of neutralizing antibody response, comprise 60 aminoacids, and each can change to 10 biochemically distinct variants. Another target protein is neuraminidase. Many of these sites are evolving at the same time [6,18,21]. Hence, the dimensionality of antigenic space in which evolution occurs is several hundred. However, empiric observations and computer simulation demonstrate that virus evolves, in the long term, along a quasi-one-dimensional trajectory [18,26,30]. The spontaneous canalization of virus trajectory in high-dimensional antigenic space occurs due to the competition between different virus variants for infectable individuals. Based on these previous results, the present model considers a one-dimensional trajectory of virus in multi–dimensional antigenic space and monitors the movement along that trajectory by a single antigenic coordinate x.

The immunity of an individual to a past infection is taken into account by recording the coordinate x of the last infecting virus, and the density of such individuals is denoted r(x), so that the integral is the total fraction of recovered individuals. Such an individual has antibodies against variant x and similar variants. The memory of older infections is expected to have a smaller effect [30] (*S4 Text*). The immune memory of a virus variant partly protects the person from infection with similar variants.

Binding between antibodies and their target regions in viral surface proteins (for influenza, hemagglutinin and neuraminidase) is sequence-specific. An aminoacid replacement in a binding region weakens binding, which effect can be measured in an antibody–binding competition essay [18]. Hence, individuals recovered from virus variants that are antigenically remote from the circulating variants are easier to infect and transmit more virions than individuals recovered from more recent variants similar to the challenge virus. This basic fact is taken into account by a cross-immunity function, defined as the normalized transmission rate of natural immunity, K(u), where u is the antigenic distance between the new virus variant and the variant that left immune memory. The normalized transmission function K(u), such that K(∞)=1, has a characteristic scale in u denoted $b_{y}.$ Parameter $b_{y}$ combines the cross-reactivity of antibodies and the breadth of the natural response characterizing the diversity of B cell receptors (antibodies) produced in response to an infection with a virus sequence. The cross-reactivity characterizes the effect of epitope variation on antibody neutralization. Below parameter $b_{y}$ is referred to as “response breadth.”

The densities of recovered r(x,t) and infected i(x,t) individuals in antigenic coordinate x change in time, as determined by the standard “infected-recovered” approach structured in antigenic coordinate [25,30] (Fig 1)

**Fig 1:**
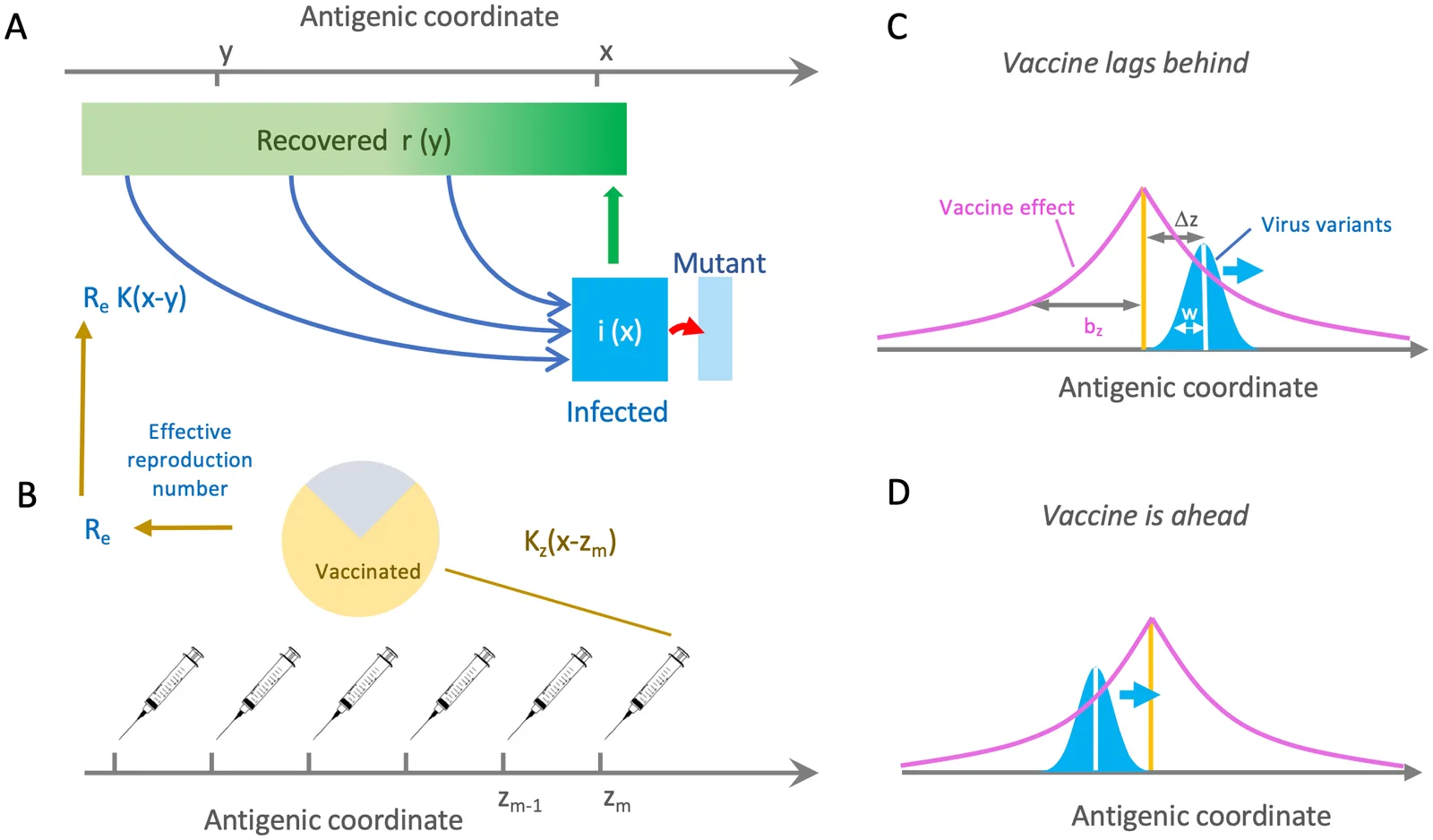
A model of respiratory virus evolution in the presence of natural immunity and vaccination. **(A)** Schematics of the model. Recovered individuals form distribution density r(y), where y is the antigenic coordinate of the viral variant an individual recovered from (green rectangle). Infected individuals form density i(x), where x is the coordinate of the current viral variant. They recover with a fixed exponential rate (green arrow). Individuals recovered from variant y can be infected with current variant x (blue arrows) with a rate proportional to $R_{e}K(x-y),$ where cross-immunity function K(u) is the normalized transmission rate in the presence of immunity. Yellow arrows show the shift of both distributions in time. **(B)** A part of population is vaccinated with sequential vectors with coordinates $z_{i}$, coverage $c_{z}<\text{1}$, and maximal efficacy $e_{z}<\text{1}$. Vaccines decrease the basic reproduction number in a naive population $R_{\text{0}}$ to an effective value $R_{e}<R_{\text{0}}.$ Relevant evolutionary factors are taken into account either by Monte-Carlo simulation or analytically by calculating effective selection coefficient and infected fraction and plugging them into the multi-locus models [30–32]. **(C)** Relevant scales in antigenic coordinate for the last vaccine and virus cloud: vaccine neutralization width $b_{z}$, vaccine lag behind the cloud center Δz, standard deviation of the infected distribution w. **(D)** Example of a vaccine ahead of the cloud center (Δz<0).


$$\begin{array}{c} \frac{\partial r(x,t)}{\partial t}=i(x,t)-r(x,t)\int_{x}^{\infty }dy\;R_{e}(y,t)i(y,t)K(y-x) \end{array}$$ (1)

$$\begin{array}{c} \frac{\partial i(x,t)}{\partial t}=-i(x,t)+R_{e}(x,t)i(x,t)\int_{-\infty }^{x}dy\;r(y,t)K(x-y) \end{array}$$

where K(u) takes into account collective immunity to past infections. Time unit is the transmission period, which is the same as the infectious period.

Eq. (1) satisfy the detailed balance condition, so that the total population size is conserved. Conservation can be checked by adding Eqs. (1), integrating inx from minus to plus infinity assuming K(p)=0 at p<0 to account for integral limits, and then swapping notation x and y in the second integral. The conservation of population size is also seen in the simulation example in Fig 2, where the net total area of two waves does not change in time. Hence one can use the normalization condition

**Fig 2:**
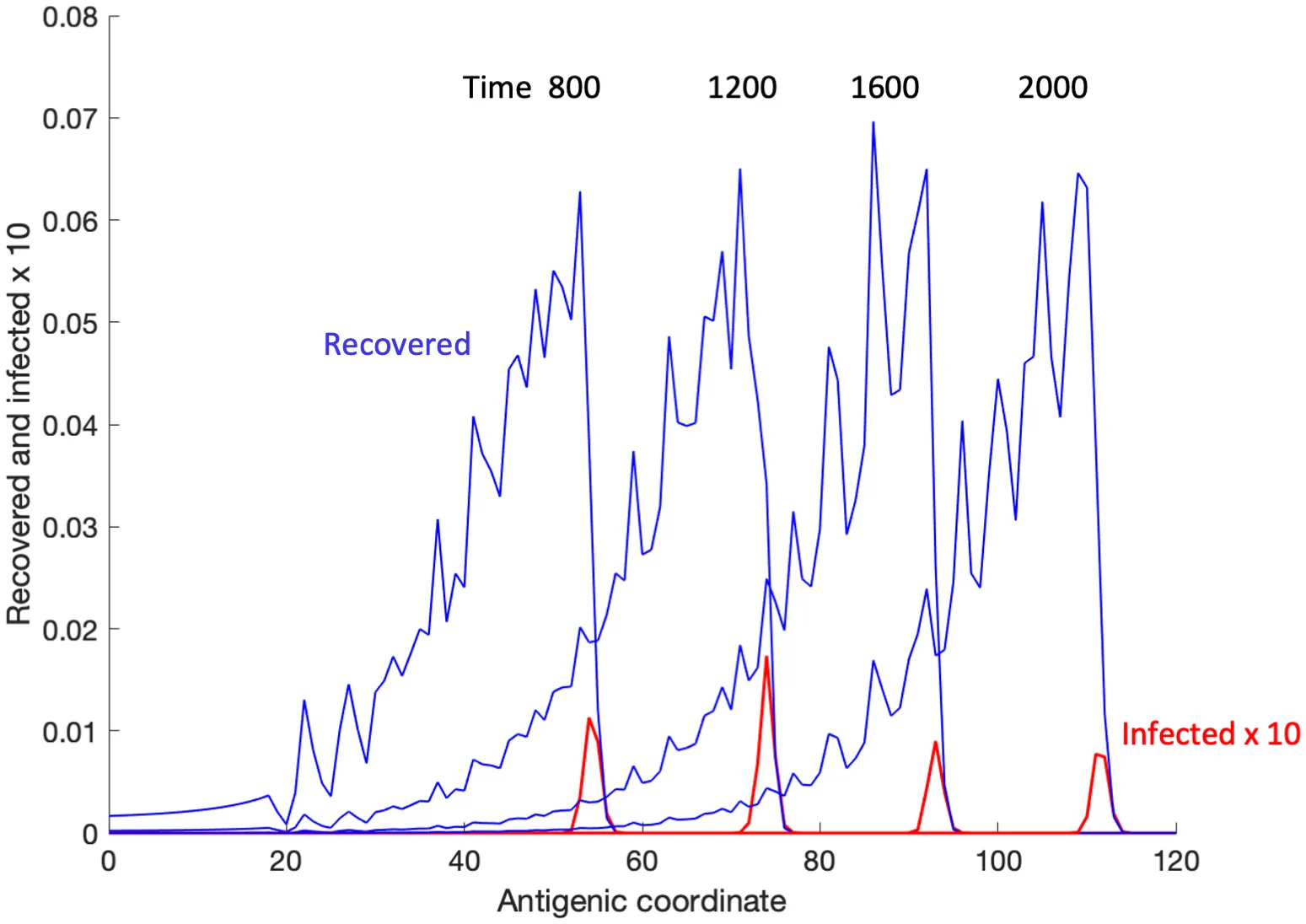
An example of Monte Carlo simulation of virus evolution for the model in Fig 1. X axis: antigenic coordinate. Y axis, blue and red lines: density of recovered and infected individuals (multiplied by 10) at different times shown in viral generations (transmission cycles). Parameters in simulation: Basic reproduction number $R_{\text{0}}=\text{1}.\text{8}$, cross-immunity function $K(p)=\left[\text{1}-exp\left(\frac{-p}{b_{y}}\right),p>\text{0};\text{0},p<\text{0}\right]$, breadth $b_{y}=\text{1}\text{1}$, mutation rate (probability) per neutralization region $U_{b}=\text{0}.\text{0}\text{0}\text{1}$, total population number ${\text{1}\text{0}}^{\text{8}}$, no vaccination. Implemented in MATLAB program *OneDwaveNew.m* at https://github.com/irouzine/RedQueen. See the full simulation in [57].


$$\begin{array}{c} \int_{-\infty }^{\infty }dx\left[i(x,t)+r(x,t)\right]=\text{1} \end{array}$$

As demonstrated in the present work, Eqs. (1) have a stationary solution, which is established after a long time has elapsed. The long-term solution does not depend on the initial conditions i(x,0),r(x,0). Only the transient dynamics before the establishment of the stationary wave, and whether it is established, depends on the initial conditions.

As the limits of integrals in Eqs. (1) imply, the model neglects infections of the individuals recovered from virus variants y ahead of the infecting virus x, i.e., with y>x. This condition is equivalent to an asymmetric kernel, K(p)=0 at p<0. The asymmetry removes a mirror wave and changes nothing else in results, because only a tiny fraction of the recovered is located in front of the infected [30].

The normalized transmission function K(p),p≡x−y, is averaged out over the level of memory B cells variable between individual patients, as well as over their antigenic polymorphism around the antigenic coordinate y. The following form was shown to interpolate well the data on influenza A virus obtained in an antibody-binding assay [57]


$$\begin{array}{c} K(p)=\frac{\text{1}}{\text{1}+\frac{e_{y}}{\text{1}-e_{y}}exp\left(\frac{-p}{b_{y}}\right)},p>\text{0} \end{array}$$ (2)

with K(0)= $\text{1}-e_{y}=\text{0}.\text{0}\text{2}\text{4}$ [57]. At p<0,K(p)≡0, as implied by the finite limits of integration in Eqs. (1). Here scale $b_{y}$ is used as a fitting parameter to fit observational data on the substitution rate, infection incidence, and the time to most recent ancestor (see the end of *Results* and S2 Text). In Eqs. (1), one neglects infections of the individuals recovered from virus variants y ahead of the infecting virus x, i.e., with y>x, which approximation introduced a small error [28,30] (S4 Text).

### Vaccination immunity

Random vaccination is included into this framework by introducing the effective reproduction number $R_{e}(x,t)$ defined as the average number of persons that would be infected by a person infected with virus x, in the presence of the immune memory left by vaccination but in the absence of previous infections. Its value is less than the basic reproduction number in a naïve population, by a factor, which depends on the antigenic distance between vaccine vector and challenge virus and the breadth $b_{z}$of the neutralization response induced by the vaccine. Generally speaking, response to infection and vaccination have different breadths, $b_{z}{\text{≠}b}_{y}.$ Thus, vaccinated individuals keep their natural immunity and acquire additional vaccine-induced immunity, and the two do not correlate (*S4 Text*). This assumption implies a simplest population model with uniform risk behavior and random vaccination. Indeed, the fraction of infected at any time is on the order of 1%, so that the correlations in a uniform population are small (*S4 Text*).

The effect of vaccination enters the model, Eq. (1), via the effective reproduction number in the presence of memory cells left by m previous vaccinations


$$\begin{array}{c} R_{e}(x,t)=R_{\text{0}}\prod_{j=\text{1}}^{m}\left[\text{1}-c_{z}\left(\text{1}-K_{z}\left(x-z_{j}\right)\right)\right],\;z_{j}\equiv jVt_{v} \end{array}$$ (3)

Here $R_{\text{0}}$ is the basic reproduction number in a naïve population, $c_{z}$ is the fraction of vaccinated population (“coverage”), and function $K_{z}\left(x-z_{j}\right)$, such that $K_{z}(\infty )=\text{1}$, is the normalized transmission rate of virus x after vaccination with a vector with antigenic coordinate $z_{j}$. Notation $\begin{array}{c} V\equiv \frac{d\overset{―}{x}}{dt} \end{array}$ is the average substitution rate, and $\overset{―}{x}(t)=\int_{-\infty }^{\infty }dxxi(x,t)$ is the average coordinate of the infected; $t_{v}$ is the time interval of vaccination. The dependence on time in Eq. (3) is step-wise: at each discrete vaccination time, the product in Eq. (3) acquires a new factor. Two relevant parameters are the last vaccine lag $\begin{array}{c} \Delta z\equiv \overset{―}{x}-z_{m} \end{array}$ and vaccination interval $\Delta z_{\text{1}}\equiv Vt_{v}$.

The following form of function $K_{z}(p)$ was shown to interpolate well the experimental data on influenza A virus [57]


$$\begin{array}{c} K_{z}(p)=\frac{\text{1}}{\text{1}+\frac{e_{z}}{\text{1}-e_{z}}exp\left(-\frac{\left|p\right|}{b_{z}}\right)} \end{array}$$ (4)

Each vaccine with vector z lowers reproduction number of virus x by a factor, that depends on the coverage and the antigenic distance between them x−z. The effective reproduction number, which is input in Eqs. (1), is a product over all vaccination events for the model version in Fig 4, and for the last vaccination event only, for the model version in Figs 2 and 3.

**Fig 3:**
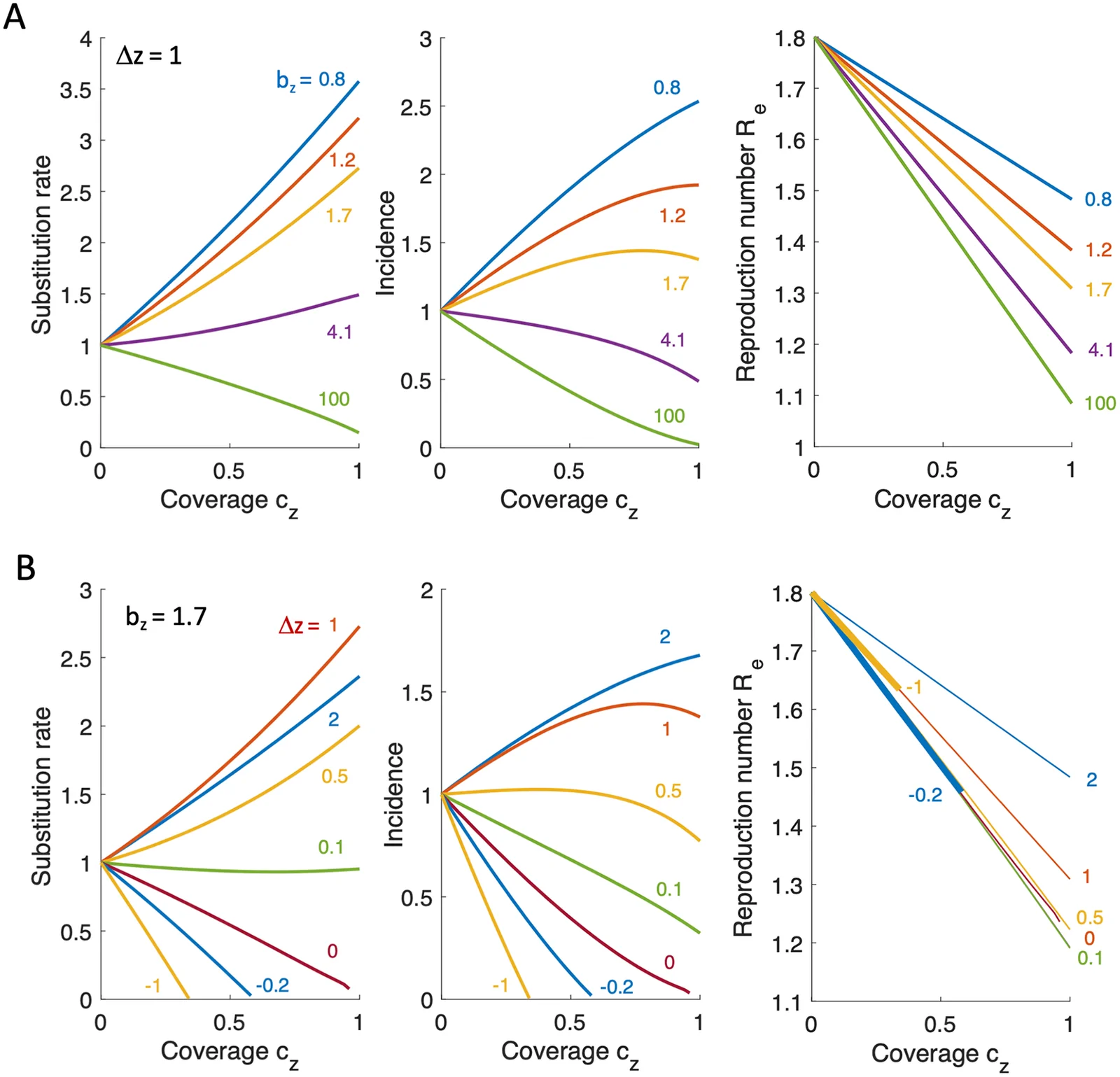
Change in the substitution rate, infection incidence, and effective reproduction number with vaccine coverage when only the last vaccination leaves memory cells. *X*-axis: Population fraction covered by vaccine $c_{z}$. **(A)** Numbers at curves is the vaccine neutralization breadth $b_{z}$ in antigenic coordinate. Vaccine lag behind the infected peak (Fig 1C) is fixed at Δz=1. **(B)** Values of vaccine lag Δz are on curves, the negatives mean that vaccine is ahead of the infected peak. Vaccine breadth is fixed at $b_{z}$= 1.7, which is the best-fit to influenza A challenge data [57]. Infected distribution width wis calculated self-consistently, as a function of coverage $c_{z}$. Fixed parameters in **(A, B)**: $R_{\text{0}}=\text{1}.\text{8}$ for influenza A H2N3 [21,64], maximal vaccine efficacy $e_{z}=\text{0}.\text{4}$ [57], population size $N={\text{1}\text{0}}^{\text{8}}.$ Natural immunity breadth $b_{y}=\text{4}.\text{1}$, mutation rate $U_{b}={\text{1}\text{0}}^{-\text{3}}$ from best fit to data on influenza A H3N2 (S2 Text). Selection coefficient is assumed to be uniformly distributed within an interval, whose average is calculated from the model (Fig 1). Default parameters are in Table 1.

### Mutation and evolutionary model

Eqs. (1) determine the dynamics of viral strains that already exist. What drives viral evolution is the emergence of new strains. Each strain x occasionally and accidentally undergoes a mutation event which changes its ability to be recognized by antibodies (phenotype). In other words, mutation causes a change in antigenic coordinate by Δx>0, where average over sites is by definition $\overset{―}{\Delta x}=\text{1}.$ The new influenza strain with a new antigenic coordinate, x+Δx, is either cleared from the individual or (with small probability) transmitted to another person.

Parameters describing random mutation are its average rate per genome per infectious period $U_{b}$ (Table 1) and the distribution density of its antigenic effect Δx. The actual distribution may be quite complex and should be measured in experiment. These relative effects vary broadly among substitutions and can be estimated from the HI assay or the transmission experiments on animals. In the present work, the phenotypic effect of mutation Δx is drawn randomly from a uniform distribution in the interval [0,2], with average 1. More complex forms of distributions are reviewed in *S2 Text*.

**Table 1:** Input parameters of the model. Parameter estimates are inferred from challenge studies and neutralization competition assays performed for influenza A H3N2 virus (see references in [57]) or obtained in S2 Text by fitting multi-locus models’ results to the observed values for the average time to the most recent common ancestor and the evolution rate of the virus.

Notation	Definition	Range, value	Source
$R_{\text{0}}$	Basic reproduction number in a naïve population	1.8	[21,64]
$t_{trans}$	Transmission cycle	5 days	
K(u)	Normalized transmission rate as a function of antigenic distance u between new and old virus	Eq. (6)	[57]
$K_{z}(u)$	Same for vaccine	Eq. (8)	[57]
$b_{y}$	Breadth/cross-reactivity of natural immunity, the scale of K(u)	4.1	fit to data in S2 Text
${\text{1}-e}_{y}=K(\text{0})$	Normalized transmission rate with homologous strain	0.024	[57]
$c_{z}$	Vaccine coverage	[0,1]	
$e_{z}=\text{1}-K_{z}(\text{0})$	Maximal vaccine efficacy	0.4	[57]
$t_{v}$	Time interval between vaccinations	0.5 year or variable	
$V_{\text{0}}$	Substitution rate in epitopes in the absence of vaccine	$\text{0}.\text{0}\text{3}\text{6}\frac{aa}{cycle}=\text{2}.\text{6}$ aa/year	[30, 46, 55, 56, 65]
Δz	Last vaccine’s lag	1.0 or variable	
$b_{z}$	Vaccine breadth in $K_{z}(u)$	1.7 or variable	[57]
N	Population size	${\text{1}\text{0}}^{\text{8}}$	
$U_{b}$	Mutation rate per epitope region per transmisson	${\text{1}\text{0}}^{-\text{3}}$	fit to data in S2 Text
ρ(s)	Distribution of selection coefficient s	uniform $\frac{\text{1}}{\text{2}\sigma },s\in [\text{0},\text{2}\sigma ]$, σ is output	
$\Delta z_{\text{1}}=V_{\text{0}}t_{v}$	Antigenic distance between vaccinations	1.3 or variable	
$\begin{array}{c} \begin{array}{c} \alpha =\frac{e_{z}c_{z}}{\Delta z_{1}} \end{array} \end{array}$	Composite vaccine parameter	variable	

Above model in Eqs. (1) is connected to the model of evolution of a haploid asexual population. Two methods can be used: numerical Monte Carlo simulation [57] and an analytic solution [30]. Because detailed simulation has been performed by another team [57], the focus of the present work is on analysis.

Monte-Carlo simulation is a computer realization of the stochastic version of Eqs. (1). Here the average number of infected $\begin{array}{c} N\;i(x) \end{array}$ at each discrete coordinate x is replaced with the Poisson random number with the same average provided it is less than 10. This replacement introduces random genetic drift. The model also includes incoming mutations into the infected compartment $\begin{array}{c} N\;i(x) \end{array}$ with average number $NU_{b}\Delta t\left[i(x+\text{1})+i(x-\text{1})\right]$ and outcoming mutations with average number $\begin{array}{c} {\text{2}NU}_{b}\Delta t\;i(x) \end{array}$. The average number of incoming mutations is replaced with its Poisson fluctuation if it is less than 10. This procedure is implemented in MATLAB program *OneDwaveNew.m* deposited at https://github.com/irouzine/RedQueen. An example of simulation is shown in Fig 2.

The analytic approach implemented in S1 Text, *Supplement online*, is based on deriving the average fitness landscape and the fraction of infected individuals from epidemiological equations, Eqs. (1). The fitness landscape defined as the number of secondary infections per infected individuals per transmission period w(x,t) is found from the second of Eq. 1 as [30]


$$\begin{array}{c} w(x,t)=\frac{\partial logi(x,t)}{\partial t}=-\text{1}+R_{e}(x,t)\int_{-\infty }^{x}dy\;r(y,t)K(x-y) \end{array}$$ (5)

where the density of recovered r(y,t) is derived analytically from Eqs. 1. Then average selection coefficient σ is calculated as the slope $\begin{array}{c} dw/dx \end{array}$ at coordinate x equal to the average coordinate of circulating virus $\begin{array}{c} \begin{array}{c} \overset{―}{x}(t)=\int_{-\infty }^{\infty }dx\;x\;i(x,t) \end{array} \end{array}$. The average selection coefficient σ is used as an input parameter for a previously–studied multi-locus model with uniformly distributed selection coefficient (S2 Text). The details of the derivation are given in S1 Text.

Annual incidence of infection is defined as the fraction of population infected during one year, which comprises all virus variants. The fraction of the infected individuals $f_{inf}$ is defined as $\begin{array}{c} \begin{array}{c} f_{inf}=\int_{-\infty }^{\infty }dx\;i(x,t) \end{array} \end{array}$, which is less than the annual incidence by the factor of 73 for the infectious period of 5 days. This value is found self-consistently in the derivation in S1 Text.

Results are obtained in two limiting cases: (i) when the reproduction number in Eq. (3) includes all vaccinations (Fig 4), and (ii) when only the most recent vaccination leaves immune memory, $z_{j}=z_{m}$ (Fig 3). These two cases are implemented in MATLAB programs *all_vaccine_w_Dz_bz.m* and *vaccine_w_Dz_bz.m*, respectively. The theoretical limit of extremely frequent vaccination is implemented separately in program *vaccine.m* (Fig 4D). All three programs are deposited at https://github.com/irouzine/Red-Queen. The model uses several assumptions based on observations on influenza and some other respiratory viruses and discussed in S4 Text.

**Fig 4:**
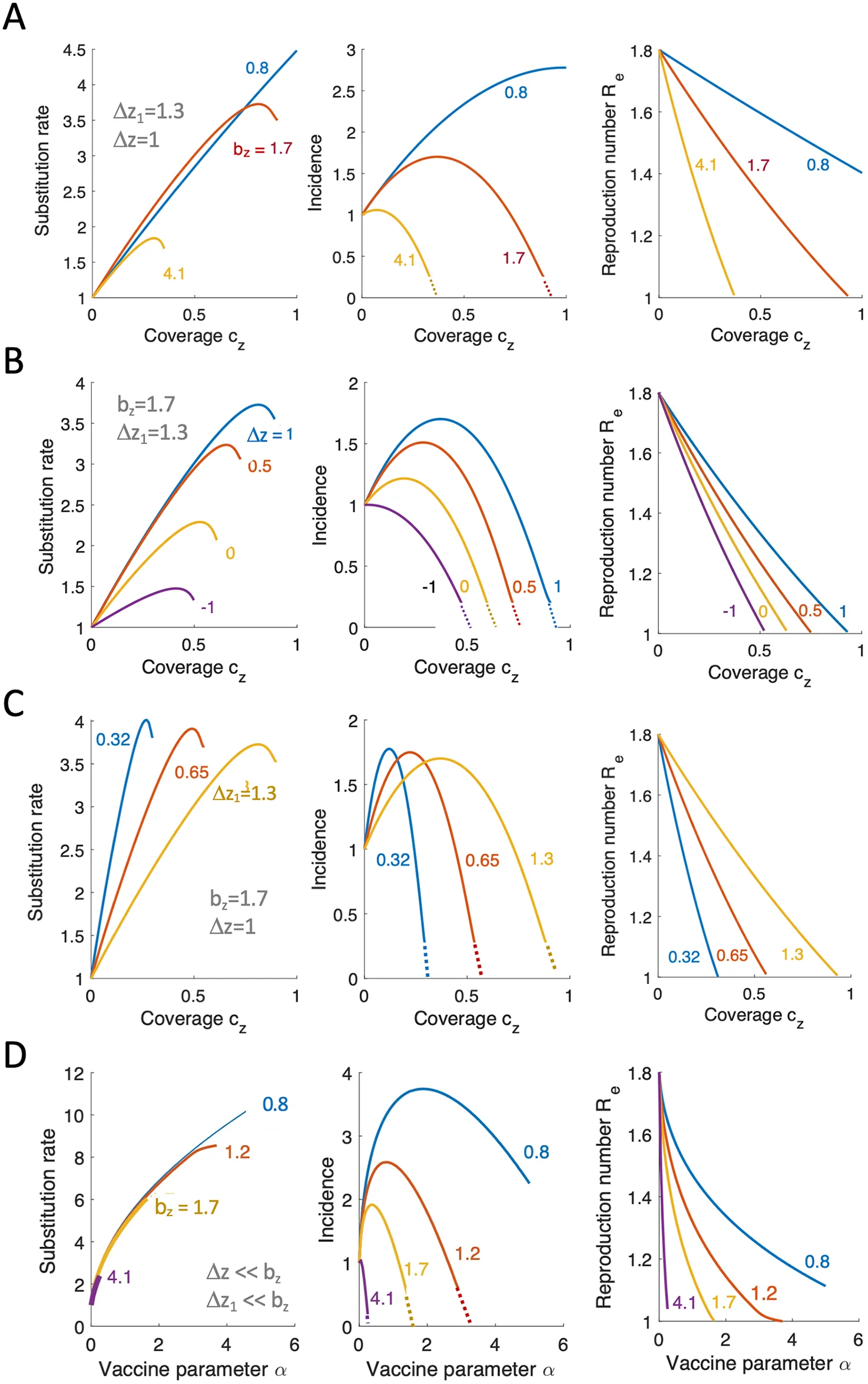
Change in the substitution rate, infection incidence, and reproduction number with vaccine coverage when consecutive vaccination events leave overlapping memory effects. *X*-axis is (A-C) population coverage by vaccine $c_{z}$ and (D) composite vaccine parameter $\alpha =e_{z}\frac{c_{z}}{\Delta }z_{\text{1}}$, where $\Delta z_{\text{1}}$ is antigenic distance between vaccinations and $e_{z}$ is maximal vaccine efficacy. **(A)** Vaccine breadth $b_{z}$ is shown on curves. Fixed parameters: antigenic distance of the last vaccine vector from the most abundant virus strain (vaccine lag) Δz=1, vaccination interval in antigenic distance $\Delta z_{\text{1}}=\text{1}.\text{3}$. **(B)** Last vaccine lag Δz is on curves, $b_{z}=\text{1}.\text{7}$ and $\Delta z_{\text{1}}=\text{1}.\text{3}$ fixed. **(C)** Vaccination interval $\Delta z_{\text{1}}$ is on curves, $b_{z}=\text{1}.\text{7}$ and Δz=1 fixed. **(D)** Hypothetical limit of very frequent vaccination ($\Delta z,\Delta z_{\text{1}}\ll b_{z}.$ Vaccine breadth $b_{z}$ is on curves. Dotted lines in (A-D) are linear interpolation to the virus extinction point where the reproduction number is equal to 1. Other parameter values as in Fig 3.

## Results

The model described in the previous section establishes a general relationship between immunological, epidemiological, and evolutionary properties of a virus in the presence of vaccination. Below the evolution rate and the incidence of influenza in a population are calculated as a function of model parameters including vaccine parameters. The model parameters in the absence of vaccine are estimated using the available data on influenza strain A H3N2. The detailed derivation is given in S1 Text.

The limits of validity are set by the following conditions. Firstly, the total number of of mutations arising in the infected part of population per transmission period must be large, $N_{inf}U_{b}\gg \text{1}$. For typical parameters estimated for influenza virus (Table 1), the condition holds in a large city or a country with millions of residents (*Discussion*). Secondly, vaccine vector is assumed to lag behind the center of virus cloud by less than the vaccine neutralization breadth, $\begin{array}{c} \Delta z\;b_{z}. \end{array}$ For modern vaccination protocols, this is usually the case. Thirdly, the number of loci is assumed to be large. The analytic derivation given in the matematical appendix (*Supplement* online, S1 Text) uses these strong inequalities to obtain approximate results neglecting small errors. Main steps of the derivation are discussed below.

### Recovered individuals and the traveling wave.

Epidemiological dynamics is analyzed in two steps [30]. First, in the realistic parameter range, the frequency of infected individuals, i(x,t), represents a solitary peak, much more narrow in antigenic distance x than the frequency of recovered individuals, r(x,t)(Fig 2). This fact allows to find the form of recovered distribution r(x,t). Second, the theory of asexual evolution is applied to obtain the evolution speed and incidence of infection. According to these models, the distribution of virus variants represents a traveling wave in fitness coordinate [46,50,55,56,58–60]. Traveling waves appear often in modeling various biological processes [61–63].

The main steps of the derivation in S1 Text are, as follows.

First, consider dynamics of the existing strains and neglect mutation. Mutation will be included later on. Assuming sufficiently frequent vaccination as stated above, $\Delta z<b_{z},$ the effective reproduction number in the presence of vaccine $R_{e}(x,t)$ given by Eq. (3) is almost constant. Apart from its small oscillations in time and coordinate, the right-hand sides of Eqs. (1) have continuous translational symmetry along antigenic coordinate.

Eqs. (1) have a traveling wave solution of the form $r(x,t)=r\left(x-\overset{―}{x}(t)\right)$ and $i(x,t)=i\left(x-\overset{―}{x}(t)\right)$, where $x-\overset{―}{x}(t)\equiv u$ is the relative antigenic coordinate of a strain. Here $\begin{array}{c} \begin{array}{c} V=\frac{d\overset{―}{x}}{dt} \end{array} \end{array}$ is the wave speed, which slowly changes in time. Because the number of loci is assumed to be very large, the velocity can be considered approximately constant within the broad time interval, larger than the time interval of wave shift by its width. By the choice of units of antigenic distance x (*Model and Methods*), this value is also equal to the average number of non-synonymous nucleotide substitutions per year. Without the loss of generality, the maximum of the infected i(u)can be chosen at u=0. An approximate solution of Eqs. (1) for infected and recovered individuals has the traveling wave form (S1 Text)


i(u)=AVf(u)

$$\begin{array}{c} (-u)=\left\{ \begin{array}{c} Aexp\left[-AR_{e}\int_{\text{0}}^{u}du^{\prime }K\left(u^{\prime }\right)\right],\;u>\text{0}\\ \text{0},\;u<\text{0} \end{array}\right. \end{array}$$ (6)

This solution is valid only in the asymptotic limit where vaccination is frequent, $\Delta z\ll b_{z}$, as assumed above. As already mentioned, effective reproduction number $R_{e}$ is almost constant, with small periodic oscillations in time and coordinate due to vaccination, Eq. (3). Hence the system of Eqs. (1) has approximate translational invariance, and the problem is reduced to that solved in [30], with $R_{e}$ replacing $R_{\text{0}}$. The oscillatory component of $R_{e}$ becomes important later on.

In Eq. (6), f(u) is a narrow peak with unit area and a width much less than that of the recovered distribution, r(u)(Fig 2). Normalization factor A in Eq. (6) is found from the normalization condition $\begin{array}{c} \begin{array}{c} \int_{-\infty }^{\text{0}}du\;r(u)\approx \text{1} \end{array} \end{array}$. At this point, the explicit form of function f(u)and the wave speed V are unknown and not required for the derivation of the average selection coefficient. Both are calculated later on in the evolutionary part of the model (S2 Text). The fraction of infected individuals


$$\begin{array}{c} f_{inf}=\int_{-\infty }^{\infty }du\;i(u)=AV \end{array}$$ (7)

is assumed to be small, which is true for influenza (${\text{1}\text{0}}^{-\text{2}}$).

Monte Carlo simulation confirms the existence of a steady traveling wave with two linked components moving in antigenic coordinate (Fig 2). Infected wave i(u) represents a narrow peak with a relatively small area. Recovered profile r(u) displays a sharp step at the maximum of i(u) and a tail decaying towards the negative coordinate. The sharp step is due to the continuous recovery of infected individuals. The decay in the tail is caused by reinfection of recovered individuals once they become genetically remote from the moving front of wave r(u). This part of derivation captures only the shape of the recovered peak leaving the narrow infected peak undefined so far. The wave stabilty is analyzed by the computer simulation in [57].

### Moving fitness landscape

In order to determine the infected individual distribution, i(u), the models of multi–locus evolution are used [46,50,55,56,58–60]. An important input parameter is fitness landscape. The important feature of natural selection caused by the immune memory is that the fitness landscape, which creates the traveling wave, travels with the wave. The recovered individuals create the fitness landscape for the evolution of the virus. Reinfection moves the recovered distribution forward in x and so on.

The form of fitness landscape is derived from the standard definition of viral fitness w(x,t) as the average number of secondary infections caused by an infected individual. Formally, fitness w(x,t) is the exponential expansion rate of infected density i(x,t) per infectious period, as given by Eq. (5), and it can be expressed in terms of relative coordinate $u=x-\overset{―}{x}(t)$ with respect to virus peak. The value of w(x,t) is positive ahead of the virus peak and negative behind the peak, indicating that virus strains are selected for in front of the infected peak and selected against in the wake of the wave, which pushes the wave forward.

In the vicinity of the infected peak at u=0, fitness landscape is approximately linear. The landscape slope σ represents the average fitness effect of mutation (average selection coefficient). From Eq. 5, the derivation in S1 Text yields


$$\begin{array}{c} \sigma ={\;\frac{\partial w(x,t)}{\partial x}|}_{u=\text{0}}=R_{e}F\left(R_{e}\right)+\frac{dR_{e}}{dx}\int_{\text{0}}^{\infty }du\;K(u)r(-u)=R_{e}F\left(R_{e}\right)+\frac{dR_{e}}{R_{e}dx} \end{array}$$ (8)

$$\begin{array}{c} F\left(R_{e}\right)\equiv \int_{\text{0}}^{\infty }du\;r(-u)\frac{dK(u)}{du} \end{array}$$

The effect of vaccination enters via the effective reproduction number $R_{e}$, which is lowered by vaccination, as given by Eq. 3. Notably, the first term in Eq. (8) decreases with vaccination coverage, because it decreases $R_{e},$while the second term is positive and increases with vaccination coverage. In other words, vaccine modifies the fitness landscape through decreasing the effective reproduction number, but when the virus moves away from vaccine in antigenic space, its reproduction number increases back. Specific expressions for Eq. (8) are given below for two limiting scenarios.

### The speed of evolution and infection incidence

So far, the speed of viral evolution V and the density of infected were left undetermined. The result for the average selection coefficient, Eq. (7), reduces the problem to the models of multi-locus evolution where the speed was expressed in terms of population size, selection coefficient and mutation rate. Two limiting scenarios regarding the immunological longevity of vaccination effect are considered. In the first scenario, only the last vaccination leaves immune memory. In the second scenario, the effect of vaccine is long-lasting and accumulates over consecutive vaccinations.

### Last-vaccination scenario

Suppose, by the time of each vaccination, the immune memory of the previous vaccination is gone. In this case, average selection coefficient has form (7), where effective reproduction number and its derivative in antigenic coordinate are replaced with their average values over circulating viral strains


$$\begin{array}{c} \left\langle R_{e}\right\rangle =R_{\text{0}} \end{array}$$

$$\begin{array}{c} \left\langle K_{z}(u+\Delta z)\right\rangle =\frac{\int_{-\infty }^{\infty }du\;i(u)K_{z}(u+\Delta z)}{\int_{-\infty }^{\infty }du\;i(u)} \end{array}$$

$$\begin{array}{c} \left\langle \frac{dR_{e}}{dx}\right\rangle =R_{\text{0}}c_{z}\left\langle \frac{dK_{z}}{dx}\right\rangle =R_{\text{0}}c_{z}\frac{\int_{-\infty }^{\infty }du\;i(u)\left\langle K_{z}^{\prime }(u+\Delta z)\right\rangle }{\int_{-\infty }^{\infty }du\;i(u)} \end{array}$$ (10)

Here infected distribution i(u) is approximated with a Gaussian with center at 0 and variance $w^{\text{2}}$, which is calculated self-consistently together with evolution rate and incidence V, as explained in S2 Text.

As follows from Eqs. (9) and (10), vaccination is causing three main effects on virus evolution, as follows. Firstly, viral transmission decreases with vaccination, i.e., $R_{e}$ in the first term in selection coefficient, Eq. (8), decreases, causing the flattening of fitness landscape and the deceleration of evolution. Another factor slowing evolution down is the reduction in the number of escape mutations due to the possible decrease in the infected population size. This factor is taken into account in the present work, but is relatively weak in the multi-locus evolution regime, where mutation rate appears only in the argument of a large logarithm [46,50,55,56,58–60]. Thirdly, vaccination accelerates virus evolution due to extra selection pressure added to the population. When virus moves away from vaccine in antigenic space, its reproduction number increases, which creates the selection pressure for escape. In Eq. (8), this effect is described by the derivative of $R_{e}$ in antigenic coordinate x. The net effect of vaccination on the evolution rate and the infection incidence is the result of competition between these opposing effects: the first and second against the third.

Two observable parameters are calculated, as follows. Substitution rate is defined as the average speed at which new substitutions are acquired by virus (in contrast to mutation rate, which is the average number of mutations per antibody binding region per transmission). Annual incidence of infection is defined as the fraction of individuals infected during one year that comprises all virus variants.

Predicted changes in viral substitution rate caused by vaccination correlate strongly with the changes in the incidence. Both observables depend strongly on the neutralization breadth of vaccine $b_{z}$ and the vaccine vector lag behind viral peak, Δz (Fig 1C and D). Depending on these parameters, vaccination may either accelerate or decelerate virus evolution and, respectively, increase or decrease the infection incidence [52] (Fig 3).

If vaccine vector lags behind the infected peak by one aminoacid substitution, Δz=1, a licensed influenza vaccine with breadth $b_{z}=\text{1}.\text{7}$ is predicted to cause accelerated evolution and, consequently, elevated infection incidence (Fig 3A). Acceleration occurs due to the increase in selection pressure by the added immune memory, despite the fact that effective reproduction number $R_{e}$ is reduced (Fig 3). Only if vaccine neutralization is as broad as neutralization induced by infection, $b_{z}>b_{y}=\text{4}.\text{1},$ vaccine will have a positive efficacy (Fig 3A, middle).

The predicted effect of vaccination is sensitive to vaccine lag Δz. If vaccine vector does not lag behind the infected peak, but is directly on top or ahead in the antigenic trajectory (Δz<0, Fig 1D), vaccination slows down evolution instead of accelerating it, and vaccine efficacy becomes positive (Fig 3B).

Moreover, when a vaccine vector is ahead of virus, vaccination can cause viral extinction when coverage exceeds a threshold (Fig 3B). The larger vaccine lead |Δz|, the lower the threshold, unless the lead of vaccine |Δz| exceeds vaccine breadth. In the example in Fig 3B, which corresponds to the licensed influenza vaccine, $b_{z}$= 1.7, the lowest coverage threshold is ~ 30% (Fig 3B). Here basic reproduction number was set at 1.8, as for influenza A H3N2. For a faster- or slower-replicating strain, the extinction threshold may be respectively higher or lower.

### Vaccination with an overlapping effect

Suppose now that vaccination has a long-lasting effect overlapping over consecutive shots. In this case, the average effective selection coefficient in Eq. (8) is obtained by substitutions of Eqs. (S11) and (S12) in S1 Text (Supplement).

As in the last-vaccination scenario, vaccine will accelerate virus evolution (Fig 4). The differences are, as follows. Firstly, evolution rate almost always increases with population coverage regardless of the vaccine breadth and lag. Secondly, the changes in incidence and evolution rate are directly linked at small coverages only. At large coverages, the change in incidence with coverage follows the change in the effective reproduction number (Fig 4, middle and last columns). As a result, the change in incidence with coverage is not monotonous. When vaccination coverage is increased, the incidence first increases, goes through a maximum, and then decreases.

Further increase in vaccination coverage may cause the reproduction number drop below unit (Fig 4, column 3). When that happens, virus goes extinct. The extinction threshold is lowered when vaccine neutralization breadth becomes larger or vaccination interval become smaller. Near the extinction threshold, the number of the infected becomes so small, that the main assumption that many sites simultaneously evolve no longer applies. Detailed analysis in the vicinity of the extinction point is given in S3 Text.

Unlike in the last-vaccine scenario, virus extinction is possible even when the last vaccine lags behind the infected peak. For a vaccine with standard width and lag Δz=1,extinction is expected at 85% of vaccinated population (Fig 4A middle). Vaccine efficacy becomes positive at 75% coverage. At smaller coverages, efficacy can be positive if vaccine is either broadly neutralizing or ahead of virus (Fig 4A, $b_{z}=\text{4}.\text{1};$Fig 4B middle, Δz=−1).

An important parameter is the interval between vaccinations in antigenic coordinate, defined as $\Delta z_{\text{1}}=V_{\text{0}}t_{v}$, where $V_{\text{0}}$ is the average aminoacid replacement rate and $t_{v}$ is the time interval. For strain H3N2 with the evolution rate of $V_{\text{0}}=\text{2}.\text{6}$ aminoacid substitutions per year, biannual vaccination corresponds to $\Delta z_{\text{1}}=\text{1}.\text{3}$. If vaccination were more frequent, infection incidence would change faster when coverage increases (Fig 4C). For example, in the case of four vaccinations per year ($\Delta z_{\text{1}}=\text{0}.\text{6}\text{5}$), 60% coverage would ensure virus eradication, even if the last vaccine vector lags behind infected peak by one substitution (red line in Fig 4C). In the hypothetical limit of very frequent vaccination considered here for completeness, the evolution rate depends only on the combination of the product of efficacy and coverage divided by vaccination interval in antigenic coordinate, $\begin{array}{c} \begin{array}{c} \frac{c_{z}e_{z}}{\Delta z_{1}} \end{array} \end{array}$ (Fig 4D).

### Fitting model parameters to data on influenza in the absence of vaccination

The virus of choice is influenza A H3N2, whose parameters are partly inferred from vaccine challenge studies and neutralization assays [57] and partly obtained in the present work by fitting the data on evolution rate and phylogeny (Table 1 and S2 Text). Model parameters fit to the data on influenza (S2 Text) are summarized in Table 1. Breadth of the immune response $b_{y}$ and beneficial mutation rate $U_{b}$ are adjusted to fit the analytic expression for the substitution rate and the time to the most recent common ancestor obtained from multi-locus models to their observed values [21,64]. The fitting procedure is described in S2 Text. Programs *scaling_genealogy.m, recomb_train.m, recomb_train2.m, testS3andS5.m*, *main_fitting.m,* and *call_fitting.m* deposited at https://github.com/irouzine/Red-Queen are used to fit data and test the accuracy of the theoretical results used for fitting.

The best-fit values of $U_{b}$ and $b_{y}$ are sensitive to the form of the distribution of selection coefficient across sites, which is unknown at the level of population. Hopefully, new development of the methods of inference will shed light on this issue [66,67]. Here we assume a quasi-uniform distribution of selection coefficient within the interval, whose average is calculated from the model, Eqs. (1), as described in S1 Text. The resulting best-fit values are $U_{b}={\text{1}\text{0}}^{-\text{3}}$ and ${\text{1}\text{0}}^{-\text{2}.\text{5}}$and $b_{y}=\text{4}.\text{1}$ and 7.2 for influenza A H3N2 and H1N1, respectively (S3 Fig). In *Results* section, only the estimates for H3N2 are used. The breadth $b_{y}=\text{4}.\text{1}$ corresponds to the half-width a=15, which is similar to the previous estimates [21,30]. The predicted annual incidence of infection lies in the realistic range of 5–10% (S3 Fig).

## Discussion

Thus, the results of the present work demonstrate that vaccine has two opposite effects on virus evolution, both stemming from the decrease of viral fitness by vaccine: primary and secondary. The primary effect is to decrease fitness and slow down evolution, and the secondary effect is to evolve strains resistant to that decrease, which accelerates evolution. This interplay between the two opposite consequences of the same intervention is similar to the evolution of resistant strains in the presence of a suboptimal antiretroviral inhbitor. First, the drug decreases virus replication, and virus load drops, but then drug-resistant strains are being selected for and fixed in a population, and virus load rebounds. Under the condition of the selection for change (Red Queen effect), this mechanics is translated into the acceleration of evolution rate. Due to the complex interplay between these opposing factors, narrowly-neutralizing vaccines have a poor or even negative efficacy, while a broad vaccine could have a good efficacy or even get virus extinct.

The conclusions of the present work agree with the intuition gained from experimental and clinical studies. It has been known for a long time that a broadly-neutralizing antibody vaccine must be used against a virus with rapidly-evolving binding regions. HIV vaccine development has been focused in this direction since mid–1990s [68]. Due to rapid evolution, the efficacy of influenza vaccine is low and sometimes even negative [35], which the present study helps to explain. One contribution of the present work is to set precise quantitative criteria of how broad must vaccine neutralization be, for the vaccine to take an effect.

Given the limitations on producing broadly-neutralizing vaccines, a new and more attractive alternative strategy is developing forward-neutralizing vaccines with vectors directly in the center of virus variant distribution or a bit ahead of it in its evolutionary trajectory. If one could create a vaccine with a vector a few months ahead in the predicted virus trajectory, even the existing vaccines with narrow neutralization and modest coverage could prove effective. The main problem here is predicting the path of evolution in multi–dimensional antigenic space. The virus cloud moves along a complex trajectory affected by natural selection and stochastic factors, such as mutation, genetic drift, genetic linkage, and, generally, recombination [69,70]. Hence, any such prediction could only be probabilistic, with the error increasing in time. Vaccine would have to be sufficiently neutralizing in all directions orthogonal to the most probable trajectory. Moreover, the act of vaccination influences the trajectory. In a highly–dimensional model, the evolutionary trajectory might steer around the vaccination vector. However, the effort of forward vaccination may succeed nonetheless. Firstly, the main force driving viral cloud is the immune memory of natural infections, which is not affected by vaccine and creates certain inertia. Secondly, we need to put vaccine vector only slightly ahead on viral path, by a single substitution (Fig 3). Selecting viral variants around the obstacle takes finite time, and until that happens, virus incidence will drop a lot, which is the aim. Of course, multi–dimensional modeling is needed to have a definitive answer. For SARS-CoV-2, prognosis of virus evolution is even more challenging, because of the variants of concern emerging several times a year [71]. Prediction of virus evolution even for a short time ahead requires the combination of adaptive landscape measurements with multi-locus modeling [24,28,66,67,72,73].

The present model considers a one-dimensional trajectory of virus, to make the problem tractable for analysis. In fact, the dimensionality of antigenic space in which evolution occurs is very high. The justification for the 1D approximation is that computer simulation in 2D and on a tree demonstrate that virus evolves, in the long term, along a quasi-one-dimensional trajectory due to the competition between different virus variants for infectable individuals. The same result is observed in the real phylogeny of the virus resembling a single “vine,” where all local branches of a clade die out leaving a single branch giving rise to a new clade [18,26,30]. The mean time to the most recent common ancestor is 3–5 years, depending on a strain [21,64]. Essentially, the derivation assumes that reinfections of the same individual occur sufficiently far apart in time and hence far apart along the stem, so that the finite width in the orthogonal direction in genetic (and antigenic) space is not important. This approximation requires careful testing in multi-dimensional simulation and will be addressed elsewhere.

A Monte Carlo simulation was recently carried out for a 2-compartment model and a 3-compartment model with separate vaccinated and unvaccinated compartments, which are equivalent to each other and to the present model in the limit of frequent-vaccination regime [57] (S5 Text). Conclusions of [57] confirm the main conclusions of the present study. Simulation [57] predicts a smaller increase of the evolution rate with vaccination coverage than obtained in the present work. It is important to realize that the present analysis is carried out in the asymptotic limit of an ideal vaccine with a large breadth $b_{z}\gg \text{1}$ and frequent vaccination, as given by the condition $\Delta z\ll b_{z}$. For the existing vaccines, whose parameters are used in [57] and for the final calculations here, both the neutralization breadth $b_{z}$ and the vaccine lag Δz are on the order of 1. In this case, the present analysis falls at the border of its validity range, which explains the discrepancy. It is important, however, that both studies arrive at the same main conclusions regarding the role of the vaccine breadth and the vaccine lag.

Several recent studies [42–44,74] used compartmental infectious disease models to predict that vaccination decreases vaccine escape by reducing the number of infectious individuals and influx of mutations. For example, Gog and colleagues [43] assumed the vaccine escape pressure to be proportional to the number of vaccinated individuals. Gandon et al [75] also have shown that the speed of adaptation to vaccination results from the balance between influx of mutations and selection. Models of this type correspond to the case when immune escape mutations emerge and spread through the population one at a time (independent-locus models). This is the case for small population sizes, such that $N_{inf}U_{b}\ll \text{1},$ where $N_{inf}$ is the total number of infected individuals, and $\begin{array}{c} U_{b}={\text{1}\text{0}}^{-\text{3}} \end{array}$ is the mutation rate, i.e., average number of copy errors per transmission per all epitopes [21,30].

For the realistic population sizes of a country, ${N=\text{1}\text{0}}^{\text{7}}-{\text{1}\text{0}}^{\text{8}}$, infected fraction $\begin{array}{c} f_{inf}=10^{-3}-10^{-2} \end{array}$, and the best-fit estimate of mutation rate from influenza data, $U_{b}={\text{1}\text{0}}^{-\text{3}}$ per transmission per epitope region (Table 1), the total number of mutations occurring in a population is large, $\begin{array}{c} N_{inf}U_{b}=10-1000 \end{array}$, where $N_{inf}={Nf}_{inf}$ is the size of infected population. In this situation, $N_{inf}U_{b}\gg \text{1}$, escape mutations emerge simultaneously at multiple sites causing strong linkage effects requiring the use of more complex multi-locus models [46,56,58,69].

In agreement with this estimate of large infected population, influenza virus and SARS-CoV-2 are observed to have hundreds of genetically diverse, simultaneously evolving sites [1–6,18,21]. Even under the unprecedented public restriction measures and vaccination efforts undertaken for SARS-CoV-2, the incidence never dropped by more than several–fold, which is not enough to break the multi-locus regime. Only in the direct vicinity of the predicted extinction point that was never reached so far, the incidence will drop more than 100–fold, so that $N_{inf}U_{b}\ll \text{1},$mutations will emerge one at a time, and one-locus models will apply.

Therefore, the evolutionary part of the present model is based on the stochastic models of multi-locus evolution [69,70]. They proved to be sufficiently useful and realistic for predicting the evolutionary properties of various viruses including HIV [65,76,77], influenza [30,73], and polio [78]. These models take into account not only natural selection and mutation, but also stochastic genetic drift and genetic linkage [45,46,49,50,69]. These models predict that the cloud of variants evolves as a solitary wave moving in genetic space towards a higher or lower fitness, depending on parameter values and initial conditions. In the case of Red Queen effect considered here, there is no chosen direction of higher fitness, and the solitary wave movement occurs along a quasi-1D trajectory in any direction to escape population immunity, but otherwise the same results apply, as long as fitness landscape is properly calculated from an SIR model [30].

The substitution rate is roughly proportional to the selection pressure, which is obtained as the output of the epidemiological model, and relatively weakly (logarithmically) depends on other parameters, such as population size and mutation rate. Recombination existing in some viruses, such as HIV and SARS-CoV-2, is predicted to partly compensate for linkage effects, depending on the frequency of co-infection with two variants [48,65,79]. However, when the total number of loci is large, even very frequent recombination cannot cancel linkage effects [80]. The reasons are that the magnitude of linkage effects increases with the locus number [45,46], and that inter-genomic correlations at homologous loci due to their common ancestors make recombination less effective [81].

Other groups also linked epidemiological models to multi-locus evolution models to describe the Red Queen virus evolution in the absence of vaccination [31,32]. Unlike the present study structuring population according to the virus variant that left immune memory in an individual [25,30], they use a mean-field approach to describe collective immune memory. That approach proposed in [29] assumes essentially that all individuals in a human or animal population share a common set of memory cells, as if they are bacteria sharing CRISPR segments. Despite of such a radical approximation, these modeling studies [31,32] obtained similar expressions for the average selection coefficient as from the memory-structured models [30]. The question of the link between different approximations remains open.

It is worth noting that the present model is formulated at the population level and does not address the clinical picture in an individual host and a negative clinical efficacy reported for some rapidly-evolving viruses [82]. Addressing this question requires a multi–level modeling including the level of individual host [83].

One may raise the question whether and to which extent HIV could be described by a modified version of the present model accounting for their persistence in a host, in contrast to the acute infection by influenza and SARS CoV-2 considered here. Like influenza, HIV has the rapid evolution in antibody-binding regions that escapes B cell response. In that sense, the situation is similar to influenza infection, albeit at a different biological level, an individual host instead of a population. The distinct feature of HIV and HCV is their interference with the antigen-specific immune cytotoxic (CTL) response. HIV infects helper CD4 T cells, rendering CTL response much less sensitive to antigen and causing viral persistence in a host. The same mechanism makes existing vaccines against HIV useseless, including CTL vaccines [38,41]. Such interference with CTL response is absent in influenza infection, which is why it is cleared from a host in a few days. Hence, the direct application of the present model to HIV remains beyond the scope of the present work.

In conclusion, an epidemiological model was combined with a multi-locus evolution model to predict the conditions of vaccination efficacy against a respiratory virus. Results demonstrate that an efficacious vaccine should be either broadly–neutralizing compared to the response induced by infection or ahead of the dominant virus variant in the evolutionary path.

### Financial disclosure

I.M.R. worked within the framework of the state assignment of the Federal Agency for Scientific Organizations (FASO Russia: topic no. 075-00264-26-00). The funders had no role in study design, data collection and analysis, decision to publish, or preparation of the manuscript. I.M.R. received salary from his organization, IEPhB RAS, which is funded by the Federal Agency for Scientific Organizations.

## Supporting information

S1 Text Derivation of the substitution rate and infection incidence in the presence of vaccination (*vaccine_w_Dz_bz.m, all_vaccine_w_Dz_bz.m, vaccine.m*). (DOC)

S2 Text Multi-locus models and fitting data on influenza (*scaling_genealogy.m, recomb_train.m, recomb_train2.m, testS3andS5.m*, *main_fitting.m, call_fitting.m*). (DOC)

S3 Text Viral extinction and the critical point. (DOC)

S4 Text Approximations and simplifications. (DOC)

S5 Text The equivalence to a model with a vaccinated compartment. (DOC)

S1 Fig Testing the relation between adaptation rate and the time to the most recent common ancestor by Monte-Carlo simulation (S2 Text). (TIFF)

S2 Fig Testing predictions of analytic theory by Monte Carlo simulation, in the case of a quasi-uniform distribution of selection coefficient (S2 Text). (TIFF)

S3 Fig Fitting data for influenza A H3N2 to analytic predictions in the absence of vaccine (S2 Text). (TIFF)

S4 Fig Delocalization of the recovered density and viral extinction, when the effective reproduction number approaches 1. (TIFF)
