# Supplementary material for: Vaccination accelerates the race of influenza virus against collective immunity
Source: PLoS Pathog. 2026 Oct 12;22(10):e1014311. doi: 10.1371/journal.ppat.1014311 (PMC13705590; doi:10.1371/journal.ppat.1014311)
Supplement: Supplementary file 2 [file ppat.1014311.s011.pdf]

Response to Reviewers

Attention: Line numbers below refer to the manuscript version with marked changes. They differ from the unmarked version.

Reviewer #1:

The manuscript presents a mathematical model on the evolutionary epidemiology of human influenza viruses. The author assumes that the populations can be stratified in terms of viruses they were or are infected by. Using the distance between the two, they compute a transmission rate. Furthermore, the model incorporates vaccination campaigns at a given year to modulate the risk of infection. Model parameters are chosen using values from earlier studies. Finally, the virus population is allowed to evolve. The author then explores the effect of parameter values related to the vaccination process (breadth, lag with the circulating virus, and coverage) on key summary statistics such as reproduction number or incidence. As a modeller, I am happy that PLoS Pathogens is sending out for review such technical articles.

The author is equally happy that PLoS Pathogens sends out articles of this kind for review and sometimes even publishes them (see, for example, Rouzine and Rozhnova 2018).

I must confess my skills are not good enough to explore the validity of the detailed calculations. However, independently of this I have major concerns about several of the underlying assumptions made. Furthermore, I find many aspects regarding the biology unclear. My knowledge of influenza virus epidemiology is probably too limited (I will refer to the other reviews on this) but I was unsure about the realism of some of the assumptions made.

The author is very grateful to Prof. Alizon for examining the biological assumptions of the model. However, as demonstrated below, his impression of "flawed assumptions" is a result of misunderstanding caused, presumably, by opaque presentation and the chosen manuscript structure, with equations placed in the back. The revised manuscript is therefore restructured and rewritten.

Before addressing specific concerns, is worth noting that the present work does not come from vaccuum but follows the wake of author's collaboration with Michael Lässig (Cologne) in 2011-2018 and Ganna Rozhnova (Utrecht) in 2011-2025. The author worked in three virological labs in USA and at Université Pierre et Marie Curie, which resulted in 45 publications on viral evolution and immunology in *PNAS, PLOS Pathogens, Journal of Virology, Cell,* and *Cell Host and Microbe*, mostly, as the leading author.

Finally, the interpretation of the results could perhaps be improved, especially since the way they are currently presented raise ethical problems.....
... Before I comment on this, I wish to stress that the way this effect is currently reported in the manuscript is ethically problematic. As scientists, especially if we work on human diseases, our work is likely to have an echo in society. This implies we have to be extremely careful about how we present our results. In the present case, stating without any precautions (even in the abstract!) that vaccines will lead to higher incidence is an issue (to use a very polite word) as it is definitely something that will be reused by anti-vaccination lobbies.

The stated goal of the manuscript is helping with the design of better vaccines. Prompting production of better vaccines by pharmaceutical companies will be the echo in society. As for the "antivaxxers", they always talk about side effects, not efficacy.

1) The model. The model is difficult to understand, partly because it is very technical, but also because it is presented both in the results and in the methods, with part of the information in both sections.

To address this important concern, the revised manuscript is restructured. The two presentations of the model are merged into a new section *Model and Methods* that goes before *Results*. The algorithm of analytic dervation in S1 Text is added to the beginning of Results. Sections *Model and Methods* and *Results* now contain more equations from *Supplement*, which helps to understand the text. Fitting parameters to data on influenza is discussed in the end of Results.

1.1 My first major issue with the model has to do with the way vaccination is modelled. If my understanding is correct, the author assume that vaccine-derived immunity is independent from natural immunity. More precisely, vaccine-related immunity is directly incorporated into the virus transmission term: the more the population was vaccinated against virus x, the less the virus circulates. In my opinion, ignoring the correlation between the two at an individual level is problematic and I think that for individuals immunised against virus x, the decrease in infection risk thanks to vaccination $R_e(y,t)$ should depend on x. The reason why the current assumption is at odds with the biology is most visible when $x=y$. If individuals are in class $s(x)$, it means that they were infected by virus x. Therefore, in this class, we expect that few of them were vaccinated and, therefore, that the population-level protection from vaccination $R_e(x,t)$ lower than for other classes $s(y)$. This is an extreme case but I think it applies more widely to the correlation structure between natural and vaccine-derived immunity.

The manuscript considers the simplest population model with uniform risk behavior and random vaccination. In this case, the correlations are negligible. In the revised version, this point is explained, as follows:

Line 280: Thus, vaccinated individuals keep their natural immunity and acquire additional vaccine-induced immunity, and the two do not correlate (S4 Text). This assumption implies a simplest population model with uniform risk behavior and random vaccination. Indeed, the fraction of infected at on time is on the order of 0.01, so that the correlations in a uniform population are small.

S4 Text, Line 1930: The model assumes that vaccine-derived immunity is independent from natural immunity. In principle, this condition could be violated due to the fact that infected people are unvaccinated. However, the annual incidence of influenza usually does not exceed 20%-40%. With 5 days per infection, this corresponds to 0.3-0.6% individuals infected at any one time, or 0.6-1.2% if the seasonal nature of epidemic is taken into account (sprint and autumn). Hence the effect of correlation creates a neglible error of ~ 1%. Significant correlations between the two kinds of immunity could arise from the nonuniform patterns of risk behavior. People who are reckless are both at a higher risk of getting infected and less likely to get vaccinated, which could create a negative correlation between two types of immunity. The focus of the present manuscript is a simple model of a population with uniform human behavior and random vaccination.

1.2 My second issue with the model itself has to do with the way the virus evolution is modelled. Here, the issue is that I could not understand how it is modelled (there is alsmot nothing in the methods and all of the detailed are into an extremely technical appendix). Based on the results, I think there probably are problematic assumptions but I cannot be more specific given the lack of clarity of this aspect of the model....
...4.4 As mentionned earlier, I could not understand the evolutionary model. Sometimes this is the case for small details (as here for part of the epidemiological model), but here it is even the general approach that is unclear. The author is referring to some of their earlier articles and to MATLAB scripts but the model itself seems very unusual with respect to influenza. There is also Appdendix S.2 but this is impossible to understand (it is as if a whole theoretical biology had been squeezed into a one page appendix).

Thank you for this comment. In the revised version, the evolutionary model is explained upfront (Line 303):

Mutation and evolutionary model. Eqs. (1) determine the dynamics of viral strains that already exist. What drives viral evolution is the emergence of new strains. Each strain x occasionally and accidentally undergoes a mutation event which changes its ability to be recognized by antibodies (phenotype). In other words, mutation causes a change in antigenic coordinate by $\Delta x > 0$, where average over sites is by definition $\overline{\Delta x} = 1$. The new influenza strain with a new antigenic coordinate, $x + \Delta x$, is either cleared from the individual or (with small probability) transmitted to another person.

Parameters describing random mutation are its average rate per genome per infectious period U_b (Table 1) and the distribution density of its antigenic effect Δx. The actual distribution may be quite complex and should be measured in experiment. These relative effects vary broadly among substitutions and can be estimated from the HI assay or the transmission experiments on animals. In the present work, the phenotypic effect of mutation Δx is drawn randomly from a uniform distribution in the interval $[0, 2]$, with average 1. More complex forms of distributions are reviewed in S2 Text.

Above model in Eq. 1 is connected to the model of evolution of a haploid asexual population. Two methods can be used: numerical Monte Carlo simulation [57] and an analytic solution [30].

Monte-Carlo simulation is a computer realization of the stochastic version of Eqs. (1). Here the average number of infected $Ni(x)$ at each discrete coordinate x is replaced with the Poisson random number with the same average provided it is less than 10. This replacement introduces random genetic drift. The model also includes incoming mutations into discrete infected compartment $Ni(x)$ with average number $NU_b\Delta t[i(x+1)+i(x-1)]$ and outcoming mutations with average number $2NU_b\Delta t\, i(x)$. The average number of incoming mutations is replaced with its Poisson fluctuation if it is less than 10. This procedure is implemented in MATLAB™ program *OneDwaveNew.m* deposited at https://github.com/irouzine/RedQueen. An example of simulation is shown in Fig. 2. Because detailed simulation for a similar model is presented by another team [57], the focus of the present work is on analysis.

The analytic approach is based on deriving the average fitness landscape and the fraction of infected individuals from epidemiological equations, Eqs. (1). The fitness landscape defined as the number of secondary infections per infected individuals per transmission period $w(x,t)$ is found from the second of Eq. 1 as [30]

$$w(x,t) = \frac{\partial \log i(x,t)}{\partial t} = -1 + R_e(x,t)\int_{-\infty}^{x} dy\, r(y,t)K(x-y) \quad (5)$$

where the density of recovered $r(y,t)$ is derived analytically from Eqs. 1. Then average selection coefficient σ is calculated as the slope $dw(x,t)/dx$ at coordinate x equal to the average coordinate of circulating virus $\bar{x}(t) = \int_{-\infty}^{\infty} dx\, x\, i(x,t)$.

Annual incidence of infection is defined as the fraction of population infected during one year, which comprises all virus variants. The fraction of the infected individuals $f_{\inf}$ is defined as $f_{\inf} = \int_{-\infty}^{\infty} dx\, i(x,t)$. This quantity is less than the annual incidence by the factor of 70 for the infectious period of 5 days. The average selection coefficient σ and the fraction of infected $f_{\inf}$ are used as input parameters for a standard multi-locus model with uniformly distributed selection coefficient reviewed in *S2 Text*. The detailed derivation is given in *S1 Text.*

2) Biological assumptions

2.1 In general, many of the biological assumptions (e.g. regarding functional shapes or parameter values) rely on an unpublished preprint from 2025 (Ref. 56), which makes it difficult to assess their validity.

The preprint, now Ref. 57, has been published:

57. Willemsen MS, Rozhnova G. Impact of vaccination on the speed of antigenic evolution. medRxiv. 2025. doi: 10.64898/2026.03.10.26347605.

Furthermore, some strong assumptions are not supported, e.g. the claim that "the virus evolves as a traveling wave" in line 185 is not backed up by any reference.

Traveling wave is not an assumption, but a result of the broad class of multi–locus models. In the present work, traveling wave is demonstrated twice: analytically (Line 414).

Eqs. (1) have a traveling wave solution of of the form $r(x,t) = r(x-\bar{x}(t))$ and $i(x,t) = i(x-\bar{x}(t))$, where $x - \bar{x}(t) \equiv u$ is the relative antigenic coordinate of a strain.

...and by Monte Carlo (see Fig. 2 below). The references are added (Line 407):

According to these models, the distribution of virus variants represents a traveling wave in fitness coordinate [46, 50, 55, 56, 58-60].

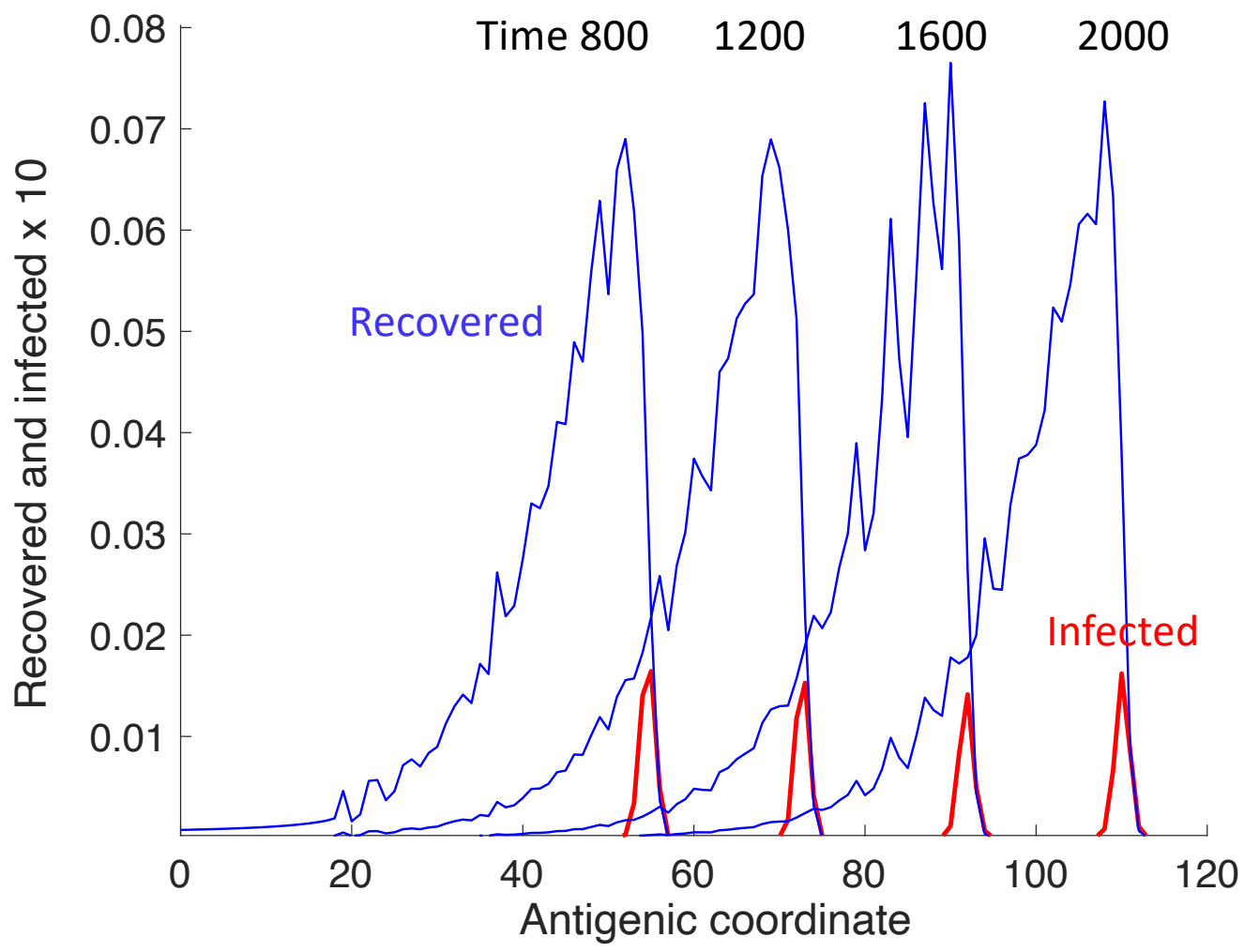

Figure 2. Monte Carlo simulation of virus evolution for the model in Fig. 1 (for illustration only). X axis: antigenic coordinate. Y axis, blue and red lines: density of recovered and infected individuals (multiplied by 10) at different times shown in viral generations (transmission cycles). Parameters in simulation: Basic reproduction number $R_0 = 2$, cross-immunity function $K(u) = 1 - \exp(-u/a)$, width of immunity to infection $a = 7$, mutation rate (probability) per neutralization region $U_b = 0.001$, total population number 10^8, no vaccination.

2.2 The author repeatedly claims that influenza evolution occurs along a one-dimensional axis. Hopefully another reviewer will be an influenza expert but, based on my limited knowledge of this virus, I thought the conclusion was that we needed two dimensions (Bedford et al. 2012 tried to implement 10 and concluded that 2 was enough). Furthermore, these earlier results are for a single gene (/hemaglutinin or neuraminidase). If the author is considering influenza in general, there should be at the very least 2 dimensions with these two proteins.

The same work, Bedford et al 2012, showed that the virus distribution in multi–dimensional antigenic space spontaneously forms a quasi–1D channel in the long–term evolution. This result was later confirmed later by Rouzine and Rozhnova 2018 on tree topology. Empiric phylogeny shows the same phenomenon (Strelkova and Lassig, 2003). The well-established fact of quasi–1D trjectory in the long term is used as a starting point of the present model. The revised manuscript explains it, as follows (Line 212):

The antibody–binding segments of hemaglutinin protein, the main target of neutralizing antibody response, comprise 60 aminoacids, and each can change to 10 biochemically distinct variants. Another target protein is neurominidase. Hence, the dimensionality of antigenic space in which evolution occurs is several hundred. However, empiric observations and computer simulation demonstrate that virus evolves, in the long term, along a quasi-one-dimensional trajectory [18, 21, 26, 27, 30, 32, 39]. The spontaneous canalization of virus trajectory in high-dimensional antigenic space occurs due to the competition between different virus variants for infectable individuals. Based on these previous results, the present model considers a one-dimensional trajectory of virus in multi–dimensional antigenic space and monitors the movement along that trajectory by a single antigenic coordinate x.

2.3 The author assumes that the distance in the antigenic space equates with a decrease in transmission rate. This needs to be better justified.

Clarified in the revision (Line 228):

Binding between antibodies and their target regions in viral surface proteins (for influenza, hemagglutinin and neuraminidase) is sequence-specific. An aminoacid replacement in a binding region weakens binding, which effect can be measured in an antibody–binding competition essay [18]. Hence, individuals recovered from virus variants that are antigenically remote from the circulating variants are easier to infect and transmit more virions than individuals recovered from more recent variants similar to the challenge virus.

2.4 I distinctly recall a presentation by Sarah Cobey stating that the year of birth, i.e. as a proxy the first influenza strain we are exposed to, has a long-lasting effect on host immunity. If this is correct, it would be strongly at odds with this model, which assumes only recent events matter.

Explained in Line 1923:

The bias of the immune system towards antibodies against the first viral strain it was exposed to is known in immunology as "original antigenic sin" or "immune imprinting". However, this effect does not matter for the population-level model, because the only parameter of the immune system that enters the model is the crossimmunity function $K(x)$, which is averaged over adults that have the imprinting by different strains. The same function is also averaged over the individual's level of memory cells left after infection or the individual's breadth of response. The form and breadth of these function are determined from fitting data.

3) Results. In general, the results appear to be rather intuitive: the vaccine works better if it is more cross-reactive and if there is not too much of a delay with the circulating strain. A very unexpected effect is that is some cases increasing vaccine coverage can increase disease incidence.

Actually, negative efficacy was reported in some field studies (Line 92):

Consequently, the efficacy of vaccination against influenza is highly variable and sometimes even negative, depending on the match of the vaccine vector to current circulating strains [33-35].

At the very least, the author should have discussed to what extent the biological assumptions and parameters ranges that lead to such behaviour are realistic or not (currently they only mention the 1-dimension issue).

Biological assumptions are discussed in Supplemental S4 Text referenced in *Model and Methods* in Line 361:

The model uses several assumptions based on empiric observations discussed in *S4 Text*.

Parameter limits are added (Line 378):

The limits of validity are set by the two following conditions. Firstly, the total number of of mutations arising in the infected part of population per transmission period must be large, $N_{inf}U_b \gg 1$. For typical parameters estimated for influenza virus (Table 1), the condition holds in a large city or a country with millions of residents (*Discussion*). Secondly, vaccine vector is assumed to lag behind the center of virus cloud by less than the vaccine neutralization breadth, $\Delta z < b_z$. For modern vaccination protocols, this is usually the case.

Regarding the nature of this "unexpected" effect, I must confess that its origin is difficult to assess since "incidence" is not defined in the article (appendix S1 is said to derive it but I could not find where). Since this "incidence" may be integrating a variety of virus over a variety of hosts at some sort of an equilibrium (since Figure 3 and 4 have a single metric for incidence), it potentially is non-trivial to define.

Annual incidence, which is a standard term in epidemiology, is now defined in Line 353:

Annual incidence of infection is defined as the fraction of population infected during one year, which comprises all virus variants. The fraction of the infected individuals f_{inf} is defined as $f_{\text{inf}} = \int_{-\infty}^{\infty} dx\, i(x,t)$. This quantity is less than the annual incidence by the factor of 73 for the infectious period of 5 days.

The author's claim is that vaccines select for different viruses. However, given the way the model is built, it is difficult to understand how this would work. Indeed, by construction, vaccination can only decrease virus circulation. Furthermore, this is a Red Queen model meaning that virus do not gain any "intrinsic" advantage in transmission rate (variations only result from antigenic

differences). Therefore, to explain this effect, we would need to envisage a situation where an inefficient vaccine selects for variants that gain a transmission advantage in the general population.

This mechanism is actually quite simple: when virus moves away from vaccine vector in antigenic space, its reproduction number increases, which means positive selection for escape. This resembles natural selection for drug resistance. The revised version explains it better (Line 700):

As follows from Eqs. (9) and (10), vaccination causes three main effects on virus evolution, as follows. Firstly, viral transmission decreases with vaccination, i.e., R_e in the first term in selection coefficient, Eq. (8), decreases, causing the flattening of fitness landscape and the deceleration of evolution. Another factor slowing down evolution is the reduction in the number of escape mutations due to the possible decrease in the infected population size. This factor is taken into account in the present work, but is relatively weak in the multi-locus evolution regime, where mutation rate appears only in the argument of a large logarithm [46, 50, 55, 56, 58-60]. Thirdly, vaccination accelerates virus evolution due to extra selection pressure added to the population. When virus moves away from vaccine in antigenic space, its reproduction number increases, which creates the selection pressure for escape. In Eq. (8), this effect is described by the derivative of R_e in antigenic coordinate x. The net effect of vaccination on the evolution rate and the infection incidence, calculated in *S1 Text*, is the result of competition between these opposing effects: the first and second against the third.

Based on the figure with the "substitution rate", which I think is more a phenotypic effect of the mutation,

Substitution rate, which is a standard term in population genetics, is now defined:

Line 358:

The output is the average substitution rate defined as the average number of non-synonymous nucleotide substitutions per year.

Line 418:

Here $V = d\bar{x}/dt$ is the wave speed. By the chosen scaling of antigenic distance, this value is also equal to the average number of non-synonymous nucleotide substitutions per year.

I am worried that the author simply assumes a direct link between vaccine coverage and the rate of phenotypic evolution (or the size of the mutations). If so, this would be a Lamarkian evolution issue because it would mean that vaccination increases evolutionary speed in general instead of only selecting variants that escape the selective pressure from the vaccine. Some of the answers may be hidden in Appendix S2 but I could not really make sense of it.

The author makes no such assumption. Vaccine enters calculations only through the effective reproduction number R_e, Eq. (3), whose definition has been moved to the front (Line 274):

Vaccination immunity. Vaccination is included into this framework by introducing the effective reproduction number $R_e(x,t)$ defined as the average number of persons that would be infected by a person infected with virus x, in the presence of the immune memory left by vaccination but in the absence of previous infections. Its value is less than the basic reproduction number in a naïve population, by a factor, which depends on the antigenic distance between vaccine vector and challenge virus and the breadth b_z of the neutralization response induced by the vaccine. Generally speaking, response to infection and vaccination have different breadths, $b_z \neq b_y$. Thus, vaccinated individuals keep their natural immunity and acquire additional vaccine-induced immunity, and the two do not correlate (S4 Text). This assumption implies a simplest population model with uniform risk behavior and random vaccination. Indeed, the fraction of the infected at any time is on the order of 0.01 or less, so that the correlations in a uniform population are small.

The effect of vaccination enters the model, Eq. (1), via the effective reproduction number in the presence of memory cells left by m previous vaccinations

$$R_e(x,t) = R_0 \prod_{j=1}^{m} \left[1 - c_z\left(1 - K_z(x - z_j)\right)\right], \qquad z_j \equiv jVt_v \tag{3}$$

Here R_0 is the basic reproduction number in a naïve population, c_z is the fraction of vaccinated population ("coverage"), and function $K_z(x - z_j)$, such that $K_z(\infty) = 1$, is the normalized transmission rate of virus x after vaccination with a vector with antigenic coordinate z_j.

More as a comment, the author presents an effect on the reproduction number R_e but this value depends on x so perhaps $R_e(x)$ would be more appropriate.

Fixed.

4) Clarity 4.1 As a general comment, the writing could be improved. This is true regarding the grammar, but also in terms of the general formulations (e.g. the first paragraphs of the introduction do not do a very good job at setting the general stage).

The author is not sure why Reviewer feels that way, but he is open to suggestions.

4.2 Regarding at topic I know better, I found the aspects related evolutionary biology often unclear. An example is at line 100 where the author writes that there is a "decrease in selective pressure due to the decrease in reproduction number". Unfortunately, they do not cite any reference to support this claim but it seems like a very strange statement because the reproduction number is a metric referring to the number of secondary infections caused by an infected host. At best, natural selection is going to act on this number, not the other way around.

In the revised manuscript, this sentence is rephrased in a more clear way (Line 125):

As demonstrated below, vaccination exerts two opposing effects on virus evolution, as follows. The deceleration due to the decrease in reproduction number caused by vaccine is opposed by acceleration due to the viral escape from additional vaccine immunity. The net effect of vaccine on the substitution rate and infection incidence is evaluated analytically, as a function of vaccine parameters, parameters of the immune system, and basic reproduction number.

There are also several finalistic statements that are at least clumsy (e.g. "in order to escape extinction in line 67).

Edited to "in order to avoid extinction".

4.3 Related to the previous point, there seems to be no distinction between mutation and substitution rate. More precisely, the author assumes that the phenotypic distance (i.e. in the antigenic space) is identical to the genetic distance (what they refer to as the substitution rate)

No such assumption is made. The manuscript states that the phenotypic (antigenic) distance **averaged** over a large number of substitutions is set to be equal to the **averaged** genetic distance by the choice of units. In other words, it is a matter of scaling (normalization). The two distances are scaled to be identical in the limit when both are very large. For a small number of substitutions, the two distances differ. This difference is now made clear (Line 167):

To account for the immunity to infections, the model classifies individuals according to virus variants they have been infected with in the past or are infected currently. Different virus variants are labeled by antigenic coordinate x representing the antigenic distance between a virus variant and the reference variant (Fig. 1). Antigenic distance is measured in amino-acid substitutions. Unlike in Hamming genetic distance, where aminoacid substitutions are simply counted, in antigenic distance they are weighted with their relative effects on the viral transmission rate, which vary broadly between sites (see below). The value of antigenic distance is normalized to be equal to the genetic distance when averaged over a large number of substitutions.

This is mainly a matter of formulation since there is no explicit fitness landscape in the model

The "missing" fitness landscape is moved from S1 Text to the front (Line 343):

The analytic approach is based on deriving the average fitness landscape and the fraction of infected individuals from epidemiological equations, Eqs. (1). The fitness landscape defined as the number of secondary infections per infected individuals per transmission period $w(x,t)$ is found from the second of Eq. 1 as [30]

$$w(x,t) = \frac{\partial \log i(x,t)}{\partial t} = -1 + R_e(x,t)\int_{-\infty}^{x} dy\, r(y,t)K(x-y) \quad (5)$$

where the density of recovered $r(y,t)$ is derived analytically from Eqs. 1. Then average selection coefficient σ is calculated as the slope $dw(x,t)/dx$ at coordinate x equal to the average coordinate of circulating virus $\bar{x}(t) = \int_{-\infty}^{\infty} dx\, x\, i(x,t)$.

...but the author should clarify that they are considering phenotypic evolution and that this may not relate to molecular evolution. 4.3 Combining the two previous points, the author seems to assume that all the regions of the genome evolve uniformly.

No such assumption is made anywhere in the manuscript.

The genome sections that are under selection will evolve much more rapidly

Indeed. This is why Introduction, from the very start, focuses reader's attention on the regions of genome of 4 viruses that bind neutralizing antibodies, undergo perpetual antigenic escape, and evolve most rapidly among other genomic regions. These are the only genomic regions considered in the manuscript. The revised version emphasizes this in Line 213:

The antibody–binding segments of hemaglutinin protein, the main target of neutralizing antibody response, comprise 60 aminoacids, and each can change to 10 biochemically–distinct variants. Another target protein is neurominidase.

The variation between sites within antibody-binding regions is taken into account as well:

Line 304:

Mutation and evolutionary model. Eqs. (1) determine the dynamics of viral strains that already exist. What drives viral evolution is the emergence of new strains due to mutation. Each strain x occasionally and accidentally undergoes a mutation event which changes its ability to be recognized by antibodies (phenotype). In other words, mutation causes a change in antigenic coordinate by $\Delta x > 0$, where average over sites is by definition $\overline{\Delta x} = 1$. The new influenza strain with a new antigenic coordinate, $x + \Delta x$, is either cleared from the individual or (with small probability) transmitted to another person.

Parameters describing random mutation are its average rate per genome per infectious period U_b (Table 1) and the distribution density of its antigenic effect Δx. The actual distribution may be quite complex and should be measured in experiment. These relative effects vary broadly among substitutions and can be estimated from the HI assay or the transmission experiments on animals. In the present work, the phenotypic effect of mutation Δx is drawn randomly from a uniform distribution in the interval $[0, 2]$, with average 1. More complex forms of distributions are reviewed in *S2 Text*.

Samuel Alizon, 24 Apr 2025

The author is grateful to Prof. Alizon for highlighting potential misunderstandings regarding the biological assumptions.

Reviewer #2:

This paper examines the theoretical effect of vaccination on the rate of adaptation of a pathogen like influenza virus. The high mutation allows the virus to escape host immunity that constantly builds up after recovery. The model assumes that the virus evolves in a 1 dimensional antigenic space. When the mutation rate is sufficiently high, the pathogen (and host immunity) is expected to reach a stationary regime where it moves at a constant speed. This speed (which can empirically measured with the substitution rate) is expected to depend on the number of infected hosts, the mutation rate and the intensity of selection. Following on previous analyses, the author argues that it is possible to derive useful approximations for the speed of viral adaptation under different vaccination scenarios. The author uses these approximations to examine the influence of vaccination on the speed of evolution and incidence. This approach can thus be used to discuss the potential risk of vaccination-driven evolution: the possibility that vaccination could speed up the rate of adaptation of the pathogen. The author argues that vaccination can accelerate viral

evolution and increase incidence and that to prevent this one may either use (1) vaccines with broader efficacy than natural immunity or (2) vaccines that are ahead in the evolutionary arms race. These conclusions are challenging the conventional wisdom that vaccination has only a weak effect on pathogen evolution. This question is interesting and I think it is important to rely on solid theoretical work to clarify the issue.

Thank you for expressing interest in the topic.

But does the present work provides this framework? I am not convinced because many points in the derivation of the model remain unclear to me.

I am grateful to Reviewer who helped to clarify the approximations of my model. However, as demonstrated below, his comments stem from misunderstanding caused, presumably, by insufficiently clear presention and the manuscript structure with important details hidden in the back.

To minimize the risk of any future misunderstanding, the revised manuscript is restructured. The two presentations of the model are merged into a new section *Model and Methods* that goes before *Results*. The algorithm of analytic dervation is moved from Supplement to the beginning of Results. Sections *Model and Methods* and *Results* now contain equations from *Supplement*, which helps to understand the text. Fitting parameters to data on influenza is discussed in the end of Results.

Impossible to check the validity of these approximations because the author does not compare predictions with simulation results...
...I don't understand why the author does not show simulation results to demonstrate the validity of his predictions. Simulations are only used to make Figure 2. ... and it is thus difficult to verify the accuracy of the approximations.

As demonstrated below, the approximations Reviewer is concerned about follow directly from empiric data. They are verified a priori and do not require simulation. Conversely, simulation cannot help to validate them.

In the present work, simulation was carried out to test the accuracy of previous multi–locus evolution model results used in the work (Fig. S1 and S2)

My main issue is the validity of the approximation used by the author to quantify the speed of adaptation. The author assumes that the system is in a stationary state and moves at a constant speed which is "roughly proportional to the selection pressure" (line 362). Under this regime, mutation is not limiting and since vaccination is increasing selection, it is generally increasing the speed of adaptation. Yet, when the coverage of vaccination is high, the incidence is expected to be low and mutations is expected to be limiting. The whole paper is built upon the assumption that the mutation rate of pathogen is very high so that one enters the "multi-locus evolution" but this requires the nb of infected * the mutation rate to be higher than 1. This cannot hold when the incidence drops so I am very unclear about the validity of the approximation.

The regime of multi-locus evolution follows from epidemiological data and several estimates of mutation rate U_b. To address this comment, the revised version reads:

Line 854: The resulting best-fit values are $U_b = 10^{-3}$ and $10^{-2.5}$ and $b_y = 4.1$ and 7.2 for influenza A H3N2 and H1N1, respectively (Fig S3).

Line 999: For the realistic population sizes of a country, $N = 10^7 - 10^8$, infected fraction $f_{\text{inf}} \sim 10^{-2}$, and the estimates of mutation rate from influenza data, $U_b = 10^{-4} - 10^{-3}$ per transmission (Table 1 and [21, 30]), the total number of mutations is $N_{\text{inf}} U_b \sim 10 - 1000$, where $N_{\text{inf}} = N f_{\text{inf}}$ is the size of infected population. In this situation, escape mutations emerge simultaneously at multiple sites causing strong linkage effects requiring the use of more complex multi-locus models [46, 56,

58, 66]. Even if the infection incidence drops several–fold due to restriction measures or vaccination, this multi-locus regime stays. Only near the theoretical extinction point, which has never been achieved so far, mutations become rare, and one could pass to one-locus evolution regime.

The author also points out that the approximation depends on the nb of loci (line 366) but never demonstrates that pathogens like the flu are characterized by many loci.

That the number of diverse sites in hemagluttinin or Spike is measured by dozens is common knowledge among virologists. The revised version clarifies it (Line 213):

The antibody–binding segments of hemagglutinin protein, the main target of neutralizing antibody response, comprise 60 aminoacids, and each can change to 10 biochemically distinct variants. Another target protein is neuraminidase. Many of these sites are evolving at the same time [6, 18, 21].

The author uses the preprint [56] which is based on stochastic simulations but the model is actually quite different

The collaborators of the author, Myrthe Willemsen and Prof. Ganna Rozhnova from Utrecht University, now cited as ref (57), considered two related models, one of which is similar to his (see S5 Text). Willemsen MS, Rozhnova G. "Impact of vaccination on the speed of antigenic evolution". medRxiv. 2025. doi: 10.64898/2026.03.10.26347605

The two models are compared in Discussion (Line 906):

A Monte Carlo simulation was recently carried out for a similar 2-compartment model and a 3-compartment model with separate vaccinated and unvaccinated compartments [58]. The 3–compartment model and the last–vaccination version of the present model are equivalent in the frequent-vaccination regime (*S5 Text*). Conclusions of the two studies agree qualitatively, but differ on the quantitative level. The simulation [58] predicts a smaller increase of the evolution rate and the infection incidence with vaccination coverage than the present work. It is possible that the quantitative difference is an artifact of a one–dimensional model used in both studies. The role of topology and dimensionality of antigenic space will be a subject of further investigation. It is important however that both studies arrive at the same conclusions regarding the role of broad and forward vaccination.

Another issue I have with the model is that many details are "hidden" in the supplementary information. It is completely impossible to understand the model based on the main text and Figure 1.

The author apologizes for inconvenience. To address this important concern, the revised manuscript is restructured: the two presentations of the model are now merged into a single section "Model and Methods" that goes before Results. The brief algorithm of derivation in S1 Text is now given in the beginning of Results.

None of the points raised in the S4 are actually discussed in the main text. I understand the aim of simplifying the message...

Correct.

...but the claim that vaccination can speed up pathogen adaptation disserves a very careful evaluation of the robustness of this effect.

The careful evaluation of assumptions is presented in S4 Text, as stated at the end of *Models and Methods*. Robustness to parameters is evident from results (Fig. 3 and 4)

The very first sentence of the abstract claims that "Respiratory viruses persist in a population because their rapid evolution allows them to escape recognition…". This is a very strong

statement that does not mention the possibility that waning immunity may also allow the persistence. The impact of waning immunity does not seem to be discussed in the model.

If immunity would wane, virus would not be pressured to evolve at such an enormous speed in antibody binding regions, 2.6 aa/year. In the revised version, a comment is added (Line 1942):

It is implied that infection leaves memory cells, which reach plateau in a few days or weeks and do not decay anymore. There is also theoretical possibility that waning immunity may contribute to persistence. However, data or models informed from data that confirm this possibility are unknown to the author. If immunity would wane, virus would not evolve at such an enormous speed, $\sim 10^{-3}$/site/year. Conversely, when virus evolves so fast, it does not matter if B cells it has escaped from wane or not. This is why all the models of influenza persistence developed by competing teams cited in the text explain persistence by evolution, not waning.

The author refers several times to HIV and HCV. The relevance of these examples is questionable because the life cycle of these viruses is very different with various forms of dormancy.

HIV and HCV are relevant in the sense that they too evolve rapidly in B cell epitopes. Introduction makes it very clear that they differ a lot from the respiratory viruses addressed in the manuscript. But since Reviewer asked about it, in an untreated chronic HIV infection, latency is very rare. Latency is important for HIV resilience to eradication by ART or during mucosal transmission (Rouzine et al 2015 Cell).

The author questions the validity of earlier studies focusing "on one-locus models of evolution that do not capture evolutionary dynamics properly" (line 86). He argues that one needs multi-locus models that account for interference between multiple mutations and refers to previous studies. Yet, it is difficult to understand from this statement why there is such a gap among previous theoretical studies. What "alters all evolutionary properties including the speed of adaptation…" (line 88).

Genetic linkage discovered by Thomas Hunt Morgan. Edited to (Line 111):

As it is known since 1930s, genetic linkage between genomic sites arising from their common ancestry alters all evolutionary properties of a population, including the speed of adaptation [45, 46], accumulation of deleterious mutations [46, 47], and the shape of phylogenetic trees [48-50]. The larger the number of evolving loci, the stronger linkage effects [46, 51].

Then he claims that "the present work is designed to shed light on this complex question" (line 94). I spent a lot of time on this draft (and on many of the references used by the author) but I am still very unclear about the robustness of the presented results. For instance, the author writes "vaccination exerts two opposing effects: a decrease in the selection pressure due to the decrease in reproduction number is opposed by an increase due to the vaccine immunity" (line 99_101). Both effects are due to increased immunity triggered by vaccination. What are the differences between these two effects remains very unclear to me. The authors starts the Discussion with a similar sentence but, again, I don't understand the difference between these two effects (both are driven by increased host immunity).
.... Line 206: three distinct effects of vaccination on virus evolution are distinguished but the differences between these effects are unclear. Are they all acting via the strength of selection or do they act via the input of mutation?

Two of these factors are acting through the decrease of viral fitness, as defined in Eq. (3), and one (the weaker one) through the input of mutation. In the revised version:

Line 125: As demonstrated below, vaccination exerts two opposing effects on virus evolution, as follows: deceleration due to the decrease in reproduction number and acceleration due to viral escape from that decrease.

Line 286: The effect of vaccination enters the model, Eq. (1), via the effective reproduction number in the presence of memory cells left by m previous vaccinations

$$R_e(x,t) = R_0 \prod_{j=1}^{m}\left[1 - c_z\left(1 - K_z(x - z_j)\right)\right], \qquad z_j \equiv jVt_v \qquad (3)$$

Line 479: The effect of vaccination enters via the effective reproduction number R_e, which is lowered by vaccination, as given by Eq. 3. Notably, the first term in Eq. (8) decreases with vaccination coverage, because it decreases R_e, while the second term is positive and increases with vaccination coverage. In other words, vaccine modifies the fitness landscape through decreasing the effective reproduction number. When virus moves away from vaccine in antigenic space, its reproduction number increases.

Line 700: As follows from Eqs. (9) and (10), vaccination is causing three main effects on virus evolution, as follows. Firstly, viral transmission decreases with vaccination, i.e., R_e in the first term in selection coefficient, Eq. (8), decreases, causing the flattening of fitness landscape and the deceleration of evolution. Another factor slowing evolution down is the reduction in the number of escape mutations due to the possible decrease in the infected population size. This factor is taken into account in the present work, but is relatively weak in the multi-locus evolution regime, where mutation rate appears only in the argument of a large logarithm [46, 50, 55, 56, 58-60]. Thirdly, vaccination accelerates virus evolution due to extra selection pressure added to the population. When virus moves away from vaccine in antigenic space, its reproduction number increases, which creates the selection pressure for escape. In Eq. (8), this effect is described by the derivative of R_e in antigenic coordinate x. The net effect of vaccination on the evolution rate and the infection incidence is the result of competition between these opposing effects: the first and second against the third.

Also, earlier studies [Gandon et al (2025) DOI: 10.1098/rsif.2025.0060] have shown that the speed of adaptation to vaccination results from the balance between (1) influx of mutations and (2) selection.

Citation is added (line 917):

For example, Gog and colleagues [43] assumed the vaccine escape pressure to be proportional to the number of vaccinated individuals. Gandon et al [71] showed that the speed of adaptation to vaccination results from the balance between influx of mutations and selection. Models of this type correspond to the case when immune escape mutations emerge and spread through the population one at a time (independent-locus models).

The argument is expected to be very different here because mutation is assumed to be non-limiting.

Correct. In the multi-site regime, mutation rate also affects the speed of evolution, but relatively weakly compared to the one-locus regime, due to the intereference between beneficial mutations accumulating at many loci at a time (see the review of multi-site models in S2 Text).

So what is the negative effect of vaccination on the speed of adaptation?

The decrease in the effective reproduction number R_e, as given by Eq. (3).

Besides, on line 331, the author seems to suggest that this negative effect is negligible. Is it?

On the contrary. The decrease in reproduction number R_e due to vaccine is the cause of the natural selection for resistant variants. Similarly, if you have an effective antiviral drug, it selects for resistant mutants unless it completely suppresses replication. The primary effect of the drug is the suppression of viral replication, and the secondary effect is the evolution of resistance.

The model assumes that vaccination changes the immunity of the host but they keep their intrinsic natural immunity.

Correct.

The author argues that it is consistent with an alternative model where unvaccinated and vaccinated hosts are distinguished. I do think it might be the case, but I find S5 very unclear. The notation does not match the one used in ref [56] which does not help.

Explanations are added to revised S5 Text:

Line 1953:

A related 3-compartment model with a separate vaccinated compartment was studied by Monte Carlo simulation in [57]. The cited authors also simulated the 2-compartment model with an effective reproduction number given by Eq. (3) in the last-vaccine scenario ($j = m$ only), similar to the model used in the present work. Below the 3-compartment model and the 2-compartment are shown to be equivalent in the limit of very frequent vaccination.

Line 2007:

The terms in the right-hand side of Eq. (S27) describe recovery of unvaccinated, natural population decline, reinfection, introduction of new uninfected into adult population. The model assumes that each vaccination cancels the effect of the previous vaccination and sends vaccinated individuals back to the unvaccinated compartment before revaccinating their fraction $c_z < 1$. This action is described by the terms with delta-functions in time. The terms in Eq. (S28) describe population decline and infection of vaccinated. The terms with delta-function are the same as in Eq. (S27), but with the opposite sign. The terms in Eq. (S29) describe the recovery of the infected, natural population decline, and the influx from vaccinated and unvaccinated.

The distinction between the two vaccination scenarios is interesting. The overlapping effect magnifies the effect of the vaccine and can lead to virus extinction for even modest vaccination coverage. The mutation rate is expected to be limiting near these extinction thresholds. The approximation used by the author is expected to collapse in these situations and this should be discussed.

Clarified in the revised version (Line 821):

Near the extinction threshold, the number of the infected becomes so small, that the main assumption that many sites simultaneously evolve no longer applies.

The discussion of alternative models of immunity is short and unclear. I did not understand the link between the present model and the mean-field approach.

The present approach is derived from the standard SIR approach fused with basic immunology and multi–locus evolution models (Lin et al 2003, Rouzine and Rozhnova 2018). The mean–field approach is postulated theoretically by a team of epidemiologists (Gog et al 2003). Comparison of alternative approaches is a complex matter that deserves a separate work. For now, I discuss it briefly (Line 999):

Other groups also linked epidemiological models to multi–locus evolution models to describe the Red Queen virus evolution in the absence of vaccination [31, 32]. Unlike the present study structuring population according to the virus variant that left immune memory in an individual [25, 30], they use a mean-field approach to describe collective immune memory. That approach proposed in [29] assumes essentially that all individuals in a human or animal population share a common set of memory cells, as if they are bacteria sharing CRISPR segments. Despite of such a radical approximation, these modeling studies [31, 32] obtained a similar expression for the average selection coefficient as from the memory-structured models [30]. The question of the link between different approximations remains open.

I find it strange to focus the perspectives on the development of vaccines that could target future variants because this seems very difficult. In contrast, current developments seem to focus on increasing cross-immunity (the b_z parameter).

Unfortunately for us all who cough and sneeze, these "current developments" go on for 30 years, with modest results. The licensed influenza vaccines have neutralization breadth only bz = 1.7 instead of 4 required for good efficacy (see Figs. 3 and 4). My immunological collaborators are skeptical that

increasing the breadth that much is even possible. In this situation, proposing an alternative strategy to the design of efficacious vaccines is very important.

Supplementary Information: a lot of things are going on p27-31. As far as I understand, the speed of adaptation is assumed to be proportional to the strength of selection...

The submitted version of the manuscript no longer uses this approximation. The difference is small though.

...which is computed numerically under different scenarios of vaccination.

Yes. More precisely, the present work is based on the analytic formulas for the substitution rate expressed in terms of the infected population size, mutation rate, and selection coefficient (Good et al 2012, S2 Text). The selection coefficient is found from the epidemiological equations, Eqs. (1). Numerical calculation is used only to solve the final system of algebraic equations.

This is highly non-trivial and I would have liked more explanations on the link between the parameters of equation (S4) and the speed of the traveling wave of the system.

To address this request, this part was moved from *S1 Text* to the front section *Model and Methods* (Line 343):

The analytic approach is based on deriving the average fitness landscape and the fraction of infected individuals from epidemiological equations, Eqs. (1). The fitness landscape defined as the number of secondary infections per infected individuals per transmission period $w(x,t)$ is found from the second of Eq. 1 as [30]

$$w(x,t) = \frac{\partial \log i(x,t)}{\partial t} = -1 + R_e(x,t) \int_{-\infty}^{x} dy\, r(y,t) K(x-y) \quad (5)$$

where the density of recovered $r(y,t)$ is derived analytically from Eqs. 1. Then average selection coefficient σ is calculated as the slope $dw(x,t)/dx$ at coordinate x equal to the average coordinate of circulating virus $\bar{x}(t) = \int_{-\infty}^{\infty} dx\, x\, i(x,t)$. Because detailed simulation for a similar model is presented by another team [57], the focus of the present work is on analysis.

Annual incidence of infection is defined as the fraction of population infected during one year, which comprises all virus variants. The fraction of the infected individuals f_{inf} is defined as $f_{\text{inf}} = \int_{-\infty}^{\infty} dx\, i(x,t)$. This quantity is less than the annual incidence by the factor of 70 for the infectious period of 5 days.

The fitness landscape and the fraction of infected are used as input parameters for a standard multi-locus model with uniformly distributed selection coefficient reviewed in *S2 Text*. Derivation is given in *S1 Text.*

A genuine description of the limits of the present model would be useful in the main text.... As indicated above, the authors does not clearly present the limits of the model.

The limits are added in Line 378:

The limits of validity are set by the two following conditions. Firstly, the total number of of mutations arising in the infected part of population per transmission period must be large, $N_{inf}U_b \gg 1$. For typical parameters estimated for influenza virus (Table 1), the condition holds in a large city or a country with millions of residents (*Discussion*). Secondly, vaccine vector is assumed to lag behind the center of virus cloud by less than the vaccine neutralization breadth, $\Delta z < b_z$. For modern vaccination protocols, this is usually the case.

Line 60: the author mentions "reproduction number" but, as far as I understand it means "transmission rate" because the duration of infection is assumed to be fixed (unit of time).

Edited to "low cost of mutation to viral fitness (reproduction number, transmission rate)"

Line 75: the author mentions the waning immunity of covid vaccines but natural immunity is also wanning and does affect the persistence.

See above.

Line 223: b_z > b_y = 4.1: The typo is fixed.

Line 274: what happens when \Delta z_1=0 on \alpha? Vaccination interval cannot be zero, one can only consider small values.

Line 427: K_z(u) -> K(u): Fixed.

Line 434: I don't understand this statement. Clarify.

Clarified: Intuitively, the virus that leaves memory is behind current strains in antigenic coordinate.

Equation (3) depends on m but you never define this parameter (the nb of vaccine shots?)

Defined: **...** reproduction number in the presence of memory cells left by m previous vaccinations

Line 452: over over: Extra word deleted.

Line 456: I don't understand the statement on the "step-like fashion".

Clarified: Eq. (3) is a discrete sum over vacine shots. When a new vaccine shot is adminstered, a new term is added to the sum, and there is an abrupt increase in R_e.

Line 975: (S16) -> (S21): The typo is fixed.

The author is indebted to the Reviewer for his help with clarifying approximations and improving presentation.

Reviewer #3:

Thanks for letting me review the manuscript titled "Vaccination accelerates the race of a respiratory virus against collective immunity". This is an interesting study relying on complex modelling. After reading it, my main though is that the Methods part is not very clear. The writing should be improved to make the study design and flow easier to understand. Because I couldn't understand the whole Methods part, I will not assess the Results now. The study addresses an important question about what is the impact of vaccination on influenza virus. The strength of the model was that cross-immunity from both natural infection and vaccination was incorporated and certain rates can be estimated by fitting data to influenza....

Incorporating vaccine strains as a vector into dynamics like travelling wave is quite unique.

Thank you.

The main weakness is that the Methods part is unclear...
...1. The Methods should be rewritten to make the description and flow clearer. Otherwise I couldn't finish reading the whole part and give critical comments.=
...1. Many derivations in the models are difficult to follow. I often need to switch back between main text and supplementary to understand the definitions. I think the main text should include more key modelling part.

As demonstrated below, Reviewer's concerns stem from manuscript's structure, with imporant details hidden in the back. I am grateful to Reviewer for highlighting this issue.

The revised manuscript is restructured. The two presentations of the model are merged into a new section *Model and Methods* that goes before *Results*. The brief algorithm of analytic dervation is added to the beginning of Results. Sections *Model and Methods* and *Results* now contain more equations from *Supplement*, which helps to understand the text. Fitting parameters to data on influenza is discussed in the end of Results.

.... and the assumption of the scenario in which a vaccination ahead of virus. In such case, one-dimensional trajectory does not make sense.
...2. Assuming that the vaccine vector is ahead of the dominant virus variant does not seem to be reasonable when using a one-dimensional antigenic trajectory. This assumption implies that virus antigenicity x will continue to move toward the vaccine vector z. However, viruses with antigenicity farther away from z will be selected. So the viruses will avoid the z. Thus, in a one-dimensional antigenic trajectory, a lagged vaccination scenario can be incorporated, but not a scenario in which a vaccination ahead of virus antigenic trajectory. In such case (vaccine vector is ahead of virus strains), two dimensional antigenic space can allow certain escaped virus species to be selected.

This point was already mentioned in Discussion. To be effective, vaccine must be only slightly ahead of the virus cloud center, by one aminoacid or less (Figs. 3 and 4). Also, a lot of selection pressure comes from the memory of past infections and past vaccines (Fig. 4). In view of these facts, it is likely that the virus will not have enough time to go around the new vaccine (provided it is equally broad in orthogonal direction) and will be suppressed by it strongly before that happens. Currently, my students are conducting multi–dimensional simulations to test this expectation. In the revised version, I clarify (Line 894):

Moreover, the act of vaccination influences the trajectory of viral cloud in antigenic space. In a highly–dimensional model, the evolutionary trajectory might steer around the vaccination vector. However, the effort of forward vaccination may succeed nonetheless. Firstly, the main force driving viral cloud is the immune memory of natural infections, which is not affected by vaccine and creates certain inertia. Secondly, we need to put vaccine vector only slightly ahead on viral path, by a single substitution (Fig. 3). Selecting viral variants around the obstacle takes finite time, and until that happens, virus incidence will drop a lot, which is the aim. Of course, multi–dimensional modeling is needed to have a definitive answer.

More often, certain notations are not described well. s was used as susceptible in the model but later s was used as selection coefficient.

In the revised version, notation s(x,t) is changed to r(x,t), which stands for "recovered".

2. The flow of the evolutionary process is quite hard to understand. For example, certain rates related to evolution were mentioned: mutation rate, substitution rate and adaptation rate. How the model link these three rates are not clearly described.

Now clarified (Line 712):

Two observable parameters are calculated, as follows. Substitution rate is defined as the average speed at which new substitutions are acquired by virus (in contrast to mutation rate, which is the average number of mutations per antibody binding region per transmission). Annual incidence of infection is defined as the fraction of individuals infected during one year that comprises all virus variants.

Line 1714:

...the adaptation rate defined as the derivative of average fitness in time. It is given by $v \equiv s_{\mathrm{eff}} V$

What are the assumptions of selection?

Fitness landscape is given in Eq. (5) (Line 344):

The fitness landscape defined as the number of secondary infections per infected individuals per transmission period $w(x,t)$ is found from the second of Eq. 1 as [30]

$$w(x,t) = \frac{\partial \log i(x,t)}{\partial t} = -1 + R_e(x,t) \int_{-\infty}^{x} dy\, r(y,t) K(x-y) \quad (5)$$

Should selection only happened at the population level that affect substitution?

All modeling is done at the host population level. The within-host level is not modeled explicitly. It enters as input parameters R_0, K(u), K_z(u) and mutation rate U_b.

What are the difference between substitution V and adaptation v.

One is the derivative of fitness in time, another is the number of substitutions acquired per unit time. The connection between the two is defined in Line 1714.

3. Ln 444, substitution rate is defined as V = dx_bar/dt. Then why in 868 x_bar = Vt. Which one dependent on the other is unclear. Why x_bar linearly increases in time given a very complex model in (1)?

Corrected (Line 418): Here $V = d\bar{x}/dt$ is the wave speed. By the chosen scaling of antigenic distance x, this value is also equal to the average number of non-synonymous nucleotide substitutions per year.

4. The use of Re is wrong. Describing Re as effective reproduction number and put that independent of s was not correct. Let say if the number of s is lower, the reproduction number from a single infected case should be lower. Re is more like a transmission rate in the model. I suggest using beta or something else in the model.
5. Ln 883 -888, again now w was defined as reproduction number. This should be fitness and clarify it was per capita rate.
6. Why need to calculate w and sigma (selection coefficient)? If w and sigma are used for estimating evolution rate, the model itself can produce the dynamics of x_bar already.

All this confusion is now resolved in *Model and Methods* (Line 343):

The analytic approach is based on deriving the average fitness landscape and the fraction of infected individuals from epidemiological equations, Eqs. (1). The fitness landscape defined as the number of secondary infections per infected individuals per transmission period $w(x,t)$ is found from the second of Eq. 1 as [30]

$$w(x,t) = \frac{\partial \log i(x,t)}{\partial t} = -1 + R_e(x,t) \int_{-\infty}^{x} dy\, r(y,t) K(x-y) \quad (5)$$

where the density of recovered $r(y,t)$ is derived analytically from Eqs. 1. Then average selection coefficient σ is calculated as the slope $dw(x,t)/dx$ at coordinate x equal to the average coordinate of circulating virus $\bar{x}(t) = \int_{-\infty}^{\infty} dx\, x\, i(x,t)$. Annual incidence of infection is defined as the fraction of population infected during one year, which comprises all virus variants. The fraction of the infected individuals f_{inf} is defined as $f_{\text{inf}} = \int_{-\infty}^{\infty} dx\, i(x,t)$. This quantity is less than the annual incidence by the factor of 70 for the infectious period of 5 days.

The average selection coefficient σ is used as input parameters for a standard multi-locus model with uniformly distributed selection coefficient reviewed in *S2 Text*. The output is the average substitution rate defined as the average number of non-synonymous nucleotide substitutions per year. The fraction of infected f_{inf} is calculated self-consistently (see Results). The detailed derivation is given in *S1 Text.* The model uses several assumptions based on empiric observations. They are discussed in *S4 Text*.

See also the beginning of *Results* (Line 416):

Eqs. (1) have a traveling wave solution of of the form $r(x,t) = r(x - \bar{x}(t))$ and $i(x,t) = i(x - \bar{x}(t))$, where $x - \bar{x}(t) \equiv u$ is the relative antigenic coordinate of a strain. Here $V = d\bar{x}/dt$ is the wave speed. By the chosen scaling of antigenic distance, this value is also equal to the average number of non-synonymous nucleotide substitutions per year. Without the loss of generality, the maximum of the infected $i(u)$ can be chosen at $u = 0$. The traveling wave solution of Eqs. (1) for infected and recovered individuals has the form

$$i(u) = AV\delta(u)$$
$$r(-u) = \begin{cases} A \exp\left[-AR_e \int_0^u du' K(u')\right] & u > 0 \\ 0 & u < 0 \end{cases} \quad (6)$$

Here $f(u)$ is a narrow peak with unit area and a width much less than that of the recovered, $r(u)$. Normalization factor A in Eq. (6) is found from the normalization condition $\int_{-\infty}^{0} du\, r(u) \approx 1$. The wave speed V and the shape of the infected density $f(u)$ are to be determined later on. The fraction of infected individuals

$$f_{\rm inf} = \int_{-\infty}^{\infty} du\, i(u) = AV \quad (7)$$

is assumed to be small, which is true for influenza (~ 10^{-2}).

Monte Carlo simulation confirms the existence of a steady traveling wave with two linked components moving in antigenic coordinate (Fig. 2). Infected wave $i(u)$ represents a narrow peak with a relatively small area. Recovered profile $r(u)$ displays a sharp step at the maximum of $i(u)$ and a tail decaying towards the negative coordinate. The sharp step is due to the continuous recovery of infected individuals. The decay in tail is caused by reinfection of recovered individuals once they become genetically remote from the moving front of wave $r(u)$. This part of derivation captures only the shape of the recovered peak leaving the narrow infected peak undefined so far.

Moving fitness landscape. In order to determine the infected individual distribution, $i(u)$, the models of multi–locus evolution are used [46, 50, 55, 56, 58-60]. An important input parameter is fitness landscape. The important feature of natural selection caused by immune memory is that the fitness landscape, which creates the traveling wave, travels with the wave. The recovered create fitness landscape for the evolution of the infected. Reinfection moves the recovered distribution forward in x and so on.

The form of fitness landscape is derived from the standard definition of viral fitness $w(x,t)$ as the average number of secondary infections caused by an infected individual. Formally, fitness $w(x,t)$ is the exponential expansion rate of infected density $i(x,t)$ per infectious period, as given by Eq. (5), and it can be expressed in terms of relative coordinate u with respect to virus peak. The value of $w(x,t)$ is positive ahead of the virus peak and negative behind the peak, indicating that virus strains are selected for in front of the infected peak and selected against in the wake of the wave, which pushes the wave forward.

In the vicinity of the narow infected peak $u = 0$, fitness landscape is approximately linear. The landscape slope σ represents the average fitness effect of mutation (average selection coefficient). From Eq. 5, one gets

$$\sigma = \left.\frac{\partial w(x,t)}{\partial x}\right|_{u=0} = R_e F(R_e) + \frac{dR_e}{dx}\int_0^{\infty} du\, K(u)\, r(-u) = R_e F(R_e) + \frac{dR_e}{R_e dx} \quad (8)$$
$$F(R_e) \equiv \int_0^{\infty} du\, r(-u) \frac{dK(u)}{du}$$

The effect of vaccination enters via the effective reproduction number R_e, which is lowered by vaccination, as given by Eq. 3. Notably, the first term in Eq. (8) decreases with vaccination coverage, because it decreases R_e, while the second term is positive and increases with vaccination coverage. In other words, vaccine modifies the fitness landscape through decreasing the effective reproduction number. When virus moves away from vaccine in antigenic space, its reproduction number increases. Specific expressions for Eq. (8) are given below for two limiting scenarios.

7. I suggest that the title be changed to something like the relationship or the impact of vaccination in Influenza Virus. The substitution pattern is largely dependent on your model assumption.

Even though the default set of parameters is chosen for influenza A, the main conclusions hold for some other respiratory viruses as well, such as SARS–CoV-2. Hence, a more general title is due.

8. ln 1002, why v = sV? If V = dx_bar/dt, then why need to have a v?

Because in the case of a distributed selection coefficient, adaptation rate v is the quantity directly calculated in the analytic theory (Good et al 2012).

The author thanks the Reviewer for his invaluable help with improving presentation.
