# Supplementary material for: Vaccination accelerates the race of influenza virus against collective immunity
Source: PLoS Pathog. 2026 Oct 12;22(10):e1014311. doi: 10.1371/journal.ppat.1014311 (PMC13705590; doi:10.1371/journal.ppat.1014311)
Supplement: Supplementary file 3 [file ppat.1014311.s012.pdf]

Line numbers below are given for the manuscript with traced changes.

Reviewer #1

The revision addresses several issues I raised...

Thank you for acknowledging this fact.

...but the manuscript remains very difficult to understand. I do think that this is a very interesting attempt to model the consequence of vaccination on the evolution of pathogens but I am expecting more than Fig 3 and 4 from theory. I mean, the analysis of a model should yield a better understanding of the process, not just a prediction. Perhaps the author has a good understanding of what is going on but, after reading the revision, I am still confused. I could probably spend more time on each step of the derivation but I feel the author should have tried harder to simplify key steps. Given the controversal nature of the claim, the argument has to be crystal clear and the manuscript is far from that. I list below some of the main issues that remain unresolved. The presentation of the model is very hard to follow. The model is complex because it mixes population dynamics and evolutionary dynamics.

Indeed, the problem tackled in the present manuscript lies at the intersection of epidemiology, immunology, virology, evolutionary modeling, and the techniques of theoretical physics. Such interdisciplinary work is not easy to read despite of all the effort spent on its presentation. The author appreciates Reviewer's patience.

Remaining concerns are addressed below.

But other models have tried to model the interplay between these two dynamics and highlighted the balance between the influx of mutation (vaccination reduces this influx) and the strength of selection (vaccination increases it). What is new here is the attempt to study the evolutionary outcome when the mutation rate is NOT limiting. The author claims that (i) mutation is not limiting in Influenza and (ii) that when it is not limiting we have to account for intereference among mutations and therefore we need to use a dedicated theoretical framework for that.

Correct.

Regarding (i): I specifically asked what was going on when the incidence drops and this assumption is not verified. In other words, it would be interesting to reconcile this formalism with previous attempts to model the effects of vaccination on the speed of adaptation. The S3 text does not really address that point. This is a pity because, again, the main value of theory is to clarify the connection with previous attempts to model similar problems. Otherwise, we are left with a collection of unrelated models that seem to compete against each other. I am genuinely expecting for more. Regarding (ii): I specificially asked about the importance of multilocus genetics to understand the adaptation to vaccination because I want to understand its importance on this specific problem. The answer of the author was to recall that linkage is known to affect evolutionary dynamics since 1930... and then jumps to more recent theory accounting for the intereference between mutations and genetic drift. But I was asking for an explanation for the transition between the two regimes (with or without limiting mutation). This is perhaps a difficult question but the author chose not to address it. Instead, he seems to argue that it is irrelevant for influenza because of the high rates

of mutation, the high nb of loci involved, the high incidence of the disease. Yet, when the vaccination coverage increases there must be a point where NinfUb<1 before the extinction threshold (as discussed by previous studies). Therefore, I think it is very important to acknowledge the different regimes of adaptation and to discuss the interface among them.

The two different regimes were already discussed (Line 616):

Several recent studies [42-44] used compartmental infectious disease models to predict that vaccination decreases vaccine escape by reducing the number of infectious individuals and influx of mutations. For example, Gog and colleagues [43] assumed the vaccine escape pressure to be proportional to the number of vaccinated individuals. Gandon et al [74] also have shown that the speed of adaptation to vaccination results from the balance between influx of mutations and selection. Models of this type correspond to the case when immune escape mutations emerge and spread through the population one at a time (independent-locus models). This is the case for small population sizes, such that $N_{\text{inf}}U_b \ll 1$, where N_{inf} is the total number of infected individuals, and $U_b \sim 10^{-3}$ is the mutation rate, i.e. average number of copy errors per transmission per all epitopes [21, 30].

For the realistic population sizes of a country, $N = 10^7 - 10^8$, infected fraction $f_{\text{inf}} \sim 10^{-2}$, and the best-fit estimate of mutation rate from influenza data, $U_b \sim 10^{-3}$ per transmission per epitope region (Table 1), the total number of mutations is $N_{\text{inf}}\,U_b \sim 100 - 1000$, where $N_{\text{inf}} = N f_{\text{inf}}$ is the size of infected population. In this situation, $N_{\text{inf}}\,U_b \gg 1$, escape mutations emerge simultaneously at multiple sites, which implies strong linkage effects requiring the use of more complex multi-locus models [46, 56, 58, 66].

To discuss the hypothetical transition between the two regimes, a new passage is added (Line 656):

In agreement with this estimate of large infected population, influenza virus and SARS-CoV-2 are observed to have hundreds of genetically diverse, simultaneously evolving sites [1-6, 18, 21]. Even under the unprecedented public restriction measures and vaccination efforts undertaken for SARS-CoV-2, the incidence never dropped by more than several–fold, which is not enough to break the multi-locus regime. Only in the direct vicinity of the predicted extinction point that was never reached so far, the incidence will drop more than 100–fold, so that $N_{\text{inf}}\,U_b \ll 1$, mutations will emerge one at a time, and one-locus models will apply.

... and in S3 Text, the last paragraph:

Influenza and SARS-CoV-2 genomes are observed to have hundreds of genetically diverse, simultaneously evolving sites (see references in Introduction). This is because these viruses evolve deeply into multi-locus regime, $N_{\text{inf}}\,U_b \sim 100 - 1000$. Only very close to the predicted extinction point, where the infection incidence drops by more than two orders of magnitude, the opposite condition will apply, $N_{\text{inf}}\,U_b \ll 1$, mutations will emerge one at a time, and vial evolution will be described by the one-locus models.

I made several comments that have not been taken into account: (i) the abstract claims that respiratory viruses persist in a population because of rates of immunity escape and does not mention the importance of waning immunity on the persistence of these viruses,...

This comment was already taken into account in the previous version. The new revision reads (Supplement, S4 Text):

An influenza or coronavirus infection is cleared within several days leaving behind memory T cells and B cells, which reach a plateau in a few days or weeks. Note that the renfection of the same person by virus occur several years apart, i.e., much later after the inital drop. There is also the theoretical possibility that the long-term waning of immunity could contribute to virus persistence. Some SIR models considered immune waning without strain-structuring the population [18-20]. However, the author is not aware of any clinical evidence that immunity against influenza keeps waning beyond the first several weeks. Moreover, the fast speed of evolution disagrees with waning immunity. If immunity would wane, the virus would not have evolve at such an enormous speed, $\sim 10^{-3}$/site/year. This is why the modern models of influenza persistence cited in Discussion neglect waning.

...(ii) the relevance of HIV and HCV is not clear because no vaccine against HIV and HCV.

This comment is new, because previously Reviewer mentioned HIV latency. To address it, a new paragraph is added (Discussion, Line 701):

One may raise the question whether and to which extent HIV could be described by a modified version of the present model accounting for their persistence in a host, in contrast to the acute infection by influenza and SARS CoV-2 considered here. Like influenza, HIV has a rapid evolution in antibody-binding regions that defies B cell response. In that sense, the situation is similar to influenza infection, albeit at a different biological level, a host instead of a population. The distinct feature of HIV and HCV is their interference with the antigen-specific immune cytotoxic (CTL) response. HIV infects helper CD4 T cells, rendering CTL response much less sensitive to antigen and causing viral persistence in a host. The same mechanism makes existing vaccines against HIV useseless, including CTL vaccines [38, 41]. Such interference with CTL response is absent in influenza infection, which is why it is cleared in several days from a host. The adaptation of the present model to HIV or HCV remains beyond the scope of the present work.

The explanation of the two opposite effects of vaccination remains very unclear. For instance around line 126 or around 793. Both effects seem to be related to selection. Yet, the author makes a distinction between "transmission" and "selection". It seems to imply that transmission is not under selection... My point here is that the reader starts the Discussion with a confusing statement and, again, I am expecting from theoretical papers to help simplify a problem.

The two opposite effects of vaccination are similar to those in drug resistance evolution. Clarified (Line 544):

Thus, the results of the present work demonstrate that vaccine has two opposite effects on virus evolution, both stemming from the decrease of viral fitness by vaccine: primary and secondary. The primary effect is to decrease fitness and hence slow down evolution, and the secondary effect is to evolve strains resistant to that decrease and accelerate evolution. This interplay between the two opposite consequences of the same intervention is similar to the evolution of resistant strains in the presence of a suboptimal antiretroviral inhbitor. First, the drug decreases virus replication and virus load drops, but then drug-resistant strains are being selected for and fixed in a population, and virus load rebounds. Under the condition of the selection for change (Red Queen effect), this mechanics is translated into acceleration of evolution rate.

The model is complex (this is inevitable given the interplay between multiple effects) but there are many parts in its presentation that need longer developments or even figures to explain what is being done. For instance, how you combine natural and vaccine immunity or what is the difference (or the relation) between py and ez (by and bz).

It is explained in detail in *Model and Methods*, lines 145-239.

Line 88: the virus do not mutate to avoid extinction or to avoid neutralization by antibodies! They mutate because replication is imperfect. This rate of mutation has evolutionary and epidemiological consequences... Reword to avoid the confusion.

Done.

Line 114 should be reworded

The comment is not clear.

Reviewer #2

The manuscript presents an investigation of analytic models important problem of the evolution of a respiratory virus in a population driven by the collective immunity left by previous infections and vaccines.My own area of expertise is in evolution modeling, including the infection spread in epidemics. I have read the text of the manuscript, the Rebuttal, and the referee reports. In my view, the manuscript represents a fair compromise between a rigorous mathematical work and biological realism. Mathematical derivations in the manuscript are solid (it is my area of expertise) and definitely more reliable than in the vast majority of contemporary biological articles on the topic. Reviewers' critiques have been adequately addressed by the author.

The author thanks Reviewer for the positive opinion about the manuscript.

In principle, I share the concern the first referee that it would be better to consider multi–dimensional space. On the other hand, the 1D assumption allows to make the problem tractable by using simple integral–differential equations, as opposed to doing numeric simulation in multi–dimensional space. I suggest the minor revisions: 1. Add a more detailed explanation regarding the validity of the one-dimensionality of antigenic space.

To address this comment, a paragraph is added to Discussion (Line 593):

The present model considers a one-dimensional trajectory of virus, to make the problem tractable for analysis. In fact, the dimensionality of antigenic space in which evolution occurs is very high. The justification for the 1D approximation is that computer simulation in 2D and on a tree demonstrate that virus evolves, in the long term, along a quasi-one-dimensional trajectory due to the competition between different virus variants for infectable individuals. The same result is observed in real phylogeny of the virus resembling a single "vine", where all local branches of a clade die out leaving a single branch giving rise to a new clade [18, 26, 30]. The mean time to the most recent common ancestor is 3-5 years, depending on a strain [21, 61]. Essentially, the derivation assumes that reinfections of the same individual occur sufficiently far apart in time and hence far apart along the stem, so that the finite width in the orthogonal direction in genetic (and antigenic) space is not important. This approximation requires careful testing in multi-dimensional simulation and will be addressed elsewhere.

2. Change the title to the more concrete one, following to the recommendation of a third referee.

"Respiratory virus" in the title is replaced with "influenza virus".

Reviewer #3

The editors sent me a revised version of the paper, along with the reviewers' questions and the author's responses. Therefore, I had the opportunity to review the discussion, which I did very carefully. Regarding the possibility of publishing the paper, I believe it should be done.

Thank you for the overall positive opinion.

Firstly, papers of this kind are, to some extent, aimed at bridging the gap between experimental biology, biological data processing, and purely mathematical modeling. Bridging these gaps is very difficult. In particular, I was very surprised to realize that the phenomenon of traveling waves in virology models is barely known, whereas the existence of such waves in models that are various modifications of the SIRV model with the addition of various effects, as well as the dependence of these waves' velocity on the reproduction number, is very well known in the modeling of biological processes and has a vast literature. Without claiming any generality, I would like to note reviews: Volpert, Petrovsky (2009, 2025), doi:10.1016/j.plrev.2009.10.002, 10.1016/j.plrev.2024.11.007, and a recent work Barker, 2026 https://doi.org/10.1016/j.matcom.2025.04.027 with the references contained therein.

The references have been added (Line 321):

Traveling waves appear often in modeling various biological processes [61-63].

Secondly, the paper raises an interesting topic and contains conclusions that will undoubtedly spark discussion. The results of the work are intuitive and seem natural.

Thank you for your intuition.

However, I have questions about the mathematical model the author uses to support his findings. I will not comment on the interpretation of the results, as I am not a virologist, and the qualifications of the respected reviewers and the author are sufficient to assess the quality of this interpretation. I would like to discuss the model and the results of its mathematical analysis, about which I have serious questions. The author belongs to a group that uses a modified model by Lin et al. [25]. This model appeared in [30]; it is quite simple and contains parameters that can be obtained from experimental data. However, I am quite confused by the analysis of this model.

This confusion is addressed below, point by point.

Clarification is added (Line 311):

The analytic derivation given in the matematical appendix (Supplement online, S1 Text) uses these strong inequalities to obtain approximate results neglecting small errors. Main steps of the derivation are listed below.

1. The solution for the traveling wave type depends on the self-similar variable u = x-ct. This is done in both [25] and [30]. However, in this paper \bar x \neq ct, so the velocity is not constant.

Clarified in Line 331:

Because the number of loci is assumed to be very large, the velocity can be considered approximately constant within the broad time interval, larger than the time interval of wave shift by its width.

2. P.7, Line 188: u=x-y. Is it different u?

Replaced with $p = x - y$.

3. Eq 3 states that R_e depends on time, the system variable. In fact, it depends on the time interval of vaccination t_v, so R_e=R_e(x) or R_e(x;t_v)

Quantity R_e depends on coordinate x, time t, and vaccination interval t_v, but the last is considered a fixed model parameter.

Clairifed (Line 229):

The dependence on time in Eq. (3) is step-wise: at each discrete vaccination time, the product in Eq. (3) acquires a new factor.

4. Now the most serious point. I do not understand how solution (6) is obtained. It does not satisfy system (1). .... Furthermore, for a traveling wave to exist, R_e must be R_e(u), but this is not the case. The solution differs from the solution given in [30] and referred to by the author. In [30], R_0 is a constant, and the existence of a traveling wave is not in doubt.

Eq. (6) is an approximate solution of Eqs. (1) in the asymptotic limit of frequent vaccination. Clarification is added (Line 335):

This solution is valid only in the asymptotic limit where vaccination is frequent, $\Delta z \ll b_z$, as assumed above. In that case, effective reproduction number R_e is almost constant, with small periodic oscillations in time and coordinate due to vaccination, Eq. (3). Hence the system of Eqs. (1) has approximate translational invariance, and the problem is reduced to that solved in [30], with R_e replacing R_0. The oscillatory component of R_e becomes important later on.

What is f (P.11, line 318)?

Another typo. Notation $\delta(u)$ in Eq. (6) is now replaced with $f(u)$.

Moreover, the results of the Monte Carlo simulation (Fig. 2) do not indicate that a delta function appears in the component i(u).

In the context of Eqs. (1), $f(u)$ is essentially a delta-function. Clarified after Eq. (6), Line 347:

In Eq. (6), $f(u)$ is a narrow peak with unit area and a width much less than that of the recovered distribution, $r(u)$ (Fig. 2).

Furthermore, I do not understand where the explicit form of this solution is used.

Explained in Line 349:

At this point, the explicit form of function $f(u)$ and the wave speed V are unknown and not required for the derivation of the average selection coefficient. Both are calculated later on in the evolutionary part of the model (S2 Text).

If the author solves system (1}, then they need initial conditions. What are these conditions?

As already mentioned, they are not important for a stationary traveling wave (Line 191):

A long-term solution of Eqs. (1) considered in the present work does not depend on the initial conditions $i(x, 0)$, $r(x, 0)$.

I have no way to verify the Monte Carlo simulation results, but I have no reason to distrust the author.

Reviewer can verify simulation results by downloading the program (Line 274):

This procedure is implemented in MATLAB program *OneDwaveNew.m* deposited at https://github.com/irouzine/RedQueen.

Note that the same traveling wave was obtained by by Willemsen and Rozhnova [57].

Figure 2 clearly shows the existence of a wave moving at a constant speed

Exactly.

(though I am confused by the note "for illustration only").

This figure is not used for calculating results shown in Fig 3 and 4 and serves only for the visualisation of the traveling wave. Words "for illustration" are removed and replaced with "Fig. 2. Example of simulation".

But since R_e is not constant,...

In the regime of frequent vaccination, R_e is almost constant, see the reply above.

...the picture must be different. What does the solution actually look like?

Similar. See the cited study by Willemsen and Rozhnova [57] who carried out the full simulation.

5. Is it possible to construct the dependence of the traveling wave velocity V on R_e with other parameters fixed?

The dependence on R_e is investigated in Supplement, S3 Text and Fig. S4.

How is this velocity found? Is this formula s25, s26?

Formula S26. As stated in *Model and Methods*, the present work assumes a uniformly distributed selection coefficient. Eq. S26 that was obtained in Good et al 2012 covers this case, if β is assumed very large.

They do not include R_e. Why are the notations for the velocity are different in s25, s26? ... 8. Where in the model the selection s (\zeta in the Table 1) is used, the different forms of distribution of which are discussed in S2?

Clarified after Eq. S26:

Eq. S26 includes parameter s_0, which is proportional to the average selection coefficient σ, as shown above. In turn, the average selection coefficient σ is is expressed in terms of R_e, as given by Eq. 8 (Eq. S3). See S1 Text.

6. A traveling wave in model [25] exists only for R_e>1. The paper does not explicitly state this, and (3) does not imply that R_e cannot become less than 1. In this case the decrease of R_e destroys the traveling wave.

In fact, the paper states viral extinction at $R_e < 1$ in six places: Lines 490 and 506, Figures 3 and 4, S3 Text, and Fig. S4.

7. The cross immunity function K(u) in the capture of Fig.2 is different from K(u) given as (2), where it is called the normalized transmission function. Are they different?

They are different. The simulation example in Fig. 2 helps to visualize the wave, but is not used for calculating the results shown in Figs 3 and 4. These are obtained analytically. For full simulation, see Willemsen and Rozhnova.

9. Are mutations taken into account in (1)? P11, line 303: "First, consider dynamics of the existing strains and neglect mutation. Mutation will be included later on". What model are we talking about here? As I understand from P9, line 255, mutation only occurs during the integration step. If this is the case, then we are solving a model different from (1).

The model structure is explained in the beginning of *Model and Methods* (Line 125):

Model. The model has two blocks, epidemiological and evolutionary, as described below. The epidemiological block is used to calculate the average selection coefficient created by the immunity of population. The average selection coefficient is plugged, as an input parameter, into a multi-locus model of evolution [55, 56].

Mutation enters the evolutionary block of the model (Line 241):

Mutation and evolutionary model. Eqs. (1) determine the dynamics of viral strains that already exist. What drives viral evolution is the emergence of new strains. Each strain x occasionally and accidentally undergoes a mutation event which changes its ability to be recognized by antibodies (phenotype). In other words, mutation causes a change in antigenic coordinate by $\Delta x > 0$, where average over sites is by definition $\overline{\Delta x} = 1$. The new influenza strain with a new antigenic coordinate, $x + \Delta x$, is either cleared from the individual or (with small probability) transmitted to another person.

Parameters describing random mutation are its average rate per genome per infectious period U_b (Table 1) and the distribution density of its antigenic effect Δx. The actual distribution may be quite complex and should be measured in experiment. These relative effects vary broadly among substitutions and can be estimated from the HI assay or the transmission experiments on animals. In the present work, the phenotypic effect of mutation Δx is drawn randomly from a uniform distribution in the interval $[0, 2]$, with average 1. More complex forms of distributions are reviewed in S2 Text.

10. The author constantly writes about memory effects. For example, "the vaccination events leave overlapping memory effects" (Fig 4). I do not understand how the memory effects are included in the model, in contrast, for example, with Liu, Sopasakis, 2024,
https://www.sciencedirect.com/science/article/pii/S2405844024143071
Taylor, Carr, 2009
https://doi.org/10.1007/s00285-009-0256-9
Khalifi, Britton, 2023
https://doi.org/10.1098/rsif.2023.0042

The three citations are added to Supplement, S4 Text, as [18-20], thank you.

These are very different models compared to the present study. The present study describes the evolutionary escape of a virus from the long-term collective immunity. This is why the SIR model, Eq. (1), is strain-structured and has a crossimmunity function K(x,y). By contrast, the three cited works do not consider any viral evolution, consider virus genetically uniform, and assume that immunity wanes in time. These models are not relevant for influenza, as explained in S4 Text:

An influenza or coronavirus infection is cleared within several days leaving behind memory T cells and B cells, which reach a plateau in a few days or weeks. Note that the renfection of the same person by virus occur several years apart, i.e., much later after the inital drop. There is also the theoretical possibility that the long-term waning

of immunity could contribute to virus persistence. Some SIR models considered immune waning without strain-structuring the population [18-20]. However, the author is not aware of any clinical evidence that immunity against influenza wanes beyond the first several weeks. Moreover, the fast speed of evolution disagrees with waning immunity. If immunity would wane, the virus would not have to evolve at such an enormous speed, $\sim 10^{-3}$/site/year. This is why the modern models of influenza persistence cited in Discussion neglect waning.

11. It would be nice to see a comparison of the findings of this work with the results of other models, for example, Wen et al. 2022, https://doi.org/10.1086/717410 where it is claimed that "On average, more vaccination decreased the incidence of infection. Notably, this decrease was driven partly by a vaccine-induced decline in the rate of antigenic evolution."

Citation added as [74], thank you. The same result appears in many studies based on one-locus models. However, one-locus models do not apply to the evolution of influenza virus or SARS CoV-2 that has hundreds of simultaneously-evolving, genetically-linked sites. This important point is already discussed in Discussion (Line 621):

Several recent studies [42-44, 74] used compartmental infectious disease models to predict that vaccination decreases vaccine escape by reducing the number of infectious individuals and influx of mutations. For example, Gog and colleagues [43] assumed the vaccine escape pressure to be proportional to the number of vaccinated individuals. Gandon et al [71] also have shown that the speed of adaptation to vaccination results from the balance between influx of mutations and selection. Models of this type correspond to the case when immune escape mutations emerge and spread through the population one at a time (independent-locus models). This is the case for small population sizes, such that $N_{\text{inf}}U_b \ll 1$, where N_{inf} is the total number of infected individuals, and $U_b \sim 10^{-3}$ is the mutation rate, i.e. average number of copy errors per transmission per all epitopes [21, 30].

For the realistic population sizes of a country, $N = 10^7 - 10^8$, infected fraction $f_{\text{inf}} \sim 10^{-2}$, and the best-fit estimate of mutation rate, $U_b = 10^{-3}$ per transmission per epitope region (Table 1), the total number of mutations is $N_{\text{inf}}\,U_b \sim 100 - 1000$, where $N_{\text{inf}} = N f_{\text{inf}}$ is the size of infected population. In this situation, escape mutations emerge simultaneously at multiple sites causing strong linkage effects requiring the use of more complex multi-locus models [46, 56, 58, 66]. Even if the infection incidence drops several–fold due to restriction measures or vaccination, this multi-locus regime stays. Only near the theoretical extinction point, which has never been achieved so far, mutations become rare, and one could pass to one-locus evolution regime.

Additional clarification is added in the revised version (Line 636):

In agreement with this estimate of large infected population, influenza virus and SARS-CoV-2 are observed to have hundreds of genetically diverse, simultaneously evolving sites. Even under the public restriction measures and unprecedented vaccination efforts undertaken for SARS-CoV-2, the incidence never drops more than several–fold, which is not enough to break the multi-locus regime. Only extremely close to the theoretical extinction point, where incidence will drop more than 100–fold, the opposite condition will take place, $N_{\text{inf}}\,U_b \ll 1$, mutations will emerge one at a time, and the evolution will be described by one-locus models.

12. Is the last equation (1) (conservation of the integral (i+r)) a consequence of the first two or is it an additional condition? If it is a consequence, then this must be proven. If this is an assumption, it may contradict the first two equations.

Clarified in Line 189:

The conservation of total population (last equation) follows from Eqs. 1 by adding them and integrating by parts.

Further, in (1), the differential sign appears immediately after the integral in the first two equations, while in the last equation, it appears at the end. This should be written consistently.

Fixed.

In summary, I can say that I am ready to accept model (1), (3), but its mathematical and numerical analysis is unconvincing and raises many questions. The mere fact of using a serious mathematical apparatus does not mean that this apparatus has been applied correctly.

The author hopes that now, after the above clarifications, the analysis looks more convincing.
