# Supplementary material for: Vaccination accelerates the race of influenza virus against collective immunity
Source: PLoS Pathog. 2026 Oct 12;22(10):e1014311. doi: 10.1371/journal.ppat.1014311 (PMC13705590; doi:10.1371/journal.ppat.1014311)
Supplement: Supplementary file 4 [file ppat.1014311.s013.pdf]

Response to Reviewer 3

1. It is claimed that the conservation of the integral from (i + r) is a consequence of the first two equations in (1). This is incorrect. This would be true if the kernel were symmetric (K(u)=K(-u)), but kernel (2) is asymmetric.

Reviewer is mistaken. The kernel is indeed asymmetric: $K(p) = 0$ at $p < 0$, as shown by the limits of integrals in (1). However, the symmetry of the kernel is not required for the conservation of the total population. Adding Eqs. (1), integrating in x over the full interval, and then swapping notation x and y in the second integral, one obtains

$$\frac{d}{dt}\int_{-\infty}^{\infty} dx\, [i(x,\, t) + r(x,t)] =$$

$$= -\int_{-\infty}^{\infty} dx \int_{-\infty}^{\infty} dy\, r(x,t)R_e(y,t)i(y,t)K(y-x) + \int_{-\infty}^{\infty} dx \int_{-\infty}^{\infty} dy\, r(y,t)R_e(x,t)i(x,t)K(x-y) = 0$$

where we assume $K(p) = 0$ at $p < 0$ to account for the integral limits in Eq. (1).

In the revised version, a clarification is added in Line 182:

Eq. (1) satisfy the detailed balance condition, so that the total population size is conserved. Conservation can be checked by adding Eqs. (1), integrating in x from minus to plus infinity assuming $K(p) = 0$ at $p < 0$ to account for integral limits, and then swapping notation x and y in the second integral. The conservation of population size is also seen in the simulation example in Fig. 2, where the net total area of two waves does not change in time. Hence one can use the normalization condition

$$\int_{-\infty}^{\infty} dx\, [i(x,\, t) + r(x,t)] = 1$$

The legend to Fig 2 is clarified, and the simulation in Fig 2 is rerun.

This problem does not exist in paper [57], to which the author constantly refers. There, the integral in nonlocal terms is taken over the entire axis, and the integral of the sum of the components is indeed conserved.

The team in [57] used almost the same kernel as the present study, see eq. (5) and table 1, where $K(u) \equiv 1 - e_z = 0.024$ in a half-interval $u > 0$. Here 0.024 is replaced with 0.

This problem also does not exist in paper [30] due to the special choice of the kernel (K(u) = 0, u > 0).

The same choice of kernel is made in the present work, except the cordinate sign is reversed, $p \equiv -u$. Clarified in line 192:

As the limits of integrals in Eqs. (1) imply, the model neglects infections of the individuals recovered from virus variants y ahead of the infecting virus x, i.e., with $y > x$. This condition is equivalent to an asymmetric kernel, $K(p) = 0$ at $p < 0$. The asymmetry removes an artifact of a mirror traveling wave and changes nothing else,

because only a tiny fraction of the recovered is located in front of the infected, and they can be safely neglected [30].

Clarified in Line 202:

At $p < 0, K(p) \equiv 0$, as implied by the finite limits of integration in Eqs. (1).

2. It is claimed that the form of the traveling wave is independent of the initial data.

The manuscript makes no such claim. The manuscript states that **the long-term stationary solution of Eqs. (1)** does not depend on the initial conditions.

This is also incorrect. In such models, both the possibility of forming a traveling wave and its velocity depend on the choice of initial data. This issue is discussed in detail in paper [57], and it is clearly stated that "Simulations were initialized from pre-generated steady-state traveling waves, equilibrated under each parameter set to avoid transient effects (Supplement 1.1)." (Beginning of Sec. 2.7)

The above quote from [57] implies that the transient dynamics of the wave depends on the initial conditions. It does not say that the long-term stationary wave depends on them. Now this point is explained better (Line 188):

As demonstrated in the present work, Eqs. (1) have a stationary solution, which is established after a long time has elapsed. The long-term solution does not depend on the initial conditions $i(x, 0), r(x, 0)$. Only the transient dynamics before the establishment of the stationary wave, and whether it is established, depends on the initial conditions.

Of course, I cannot demand a rigorous mathematical justification and stability of the traveling wave,...

Indeed, the work is written in the pragmatic style of theoretical physics, where one checks such things by simulation (Fig. 2). Clarified (Line 414):

The wave stabilty is analyzed by the computer simulation in [57].

... but now I have doubts about the very model that underlies the calculations.

The doubts are caused by misunderstanding, see the reply to Comment 1.

3. Regarding the formulation of the model: it should be clearly stated that it consists of the first two equations (1), and the third equation should not be included in the model; it should be a consequence of the first two, but in its current form, it is not a consequence).

Yes, it is, see the reply to Comment 1.

Throughout the text, dx should be either before or after the integrand, consistently. Preferably after.

Made consistent throughout.

4. I do not understand formula (8). It should be derived from (5), which is an analogue of (7) from [30].

The derivation of Eq. (8) is given in the mathematical appendix, *S1 Text*, cited in the text in several places. Added before Eq. (8):

From Eq. (5), the derivation given in *S1 Text* online yields

But then, apparently, the integral should be taken over the entire axis.

Integrals over the entire axis and the integrals with limits in Eq. (1) are the same thing, because at $p < 0, K(p) \equiv 0$.

Further, the derivative is taken incorrectly in any case.

Another wrong claim. The derivative is correct, please read *S1 Text.*

I believe all stages of the calculations should be presented in detail, in the spirit of [57]. Otherwise, the reasoning is unconvincing to the reader.

All stages of the analytic derivation are presented in detail in mathematical appendix in *S1 Text,* which is cited in the main text. Section *Results* contains the results.

Such structure is common for analytical studies published in biological journals, because the readership includes wet lab scientists and bioinformaticians who cannot read complex mathematical derivations. Paper [57] is a numerical study.
