# Supplementary material for: Vaccination accelerates the race of influenza virus against collective immunity
Source: PLoS Pathog. 2026 Oct 12;22(10):e1014311. doi: 10.1371/journal.ppat.1014311 (PMC13705590; doi:10.1371/journal.ppat.1014311)
Supplement: Supplementary file 5 [file ppat.1014311.s001.doc]

Supporting information for article

**"Vaccination accelerates the race of virus against collective immunity"**

Igor M. Rouzine

Sechenov Institute of Evolutionary Physiology and Biochemistry RAS

St Petersburg 194223, Russia, igor.rouzine@iephb.ru

Contents:

S1 Text. Derivation of the substitution rate and infection incidence in the presence of vaccination (programs *vaccine_w_Dz_bz.m, all_vaccine_w_Dz_bz.m, vaccine.m*).

S2 Text. Multi-locus models and fitting data on influenza (programs *scaling_genealogy.m,* *recomb_train.m, recomb_train2.m, testS3andS5.m*, *main_fitting.m,* and *call_fitting.m*).

S3 Text. Viral extinction and the critical point.

S4 Text. Approximations and simplifications.

S5 Text. The equivalence to a model with a vaccinated compartment.

Figure S1. Testing the relation between adaptation rate and the time to the most recent common ancestor by Monte-Carlo simulation (S2 Text).

Figure S2. Testing predictions of analytic theory by Monte Carlo simulation, in the case of a quasi-uniform distribution of selection coefficient (S2 Text).

Figure S3. Fitting data for influenza A H3N2 to analytic predictions in the absence of vaccine (S2 Text).

Figure S4. Delocalization of the recovered density and viral extinction, when the effective reproduction number approaches 1.

**S1 Text. Derivation of the substitution rate and infection incidence in the presence of vaccination**

Assuming sufficiently frequent vaccination, effective reproduction number in the presence of vaccine $R_{e}$ weakly oscillates in antigenic coordinate and time. Hence, in the first approximation, the right-hand sides of Eqs. (1) in the main text have continuous translational symmetry. They have a traveling wave solution of a form

$i\left( x,t \right)=i\left( u \right), r\left( x,t \right)=r\left( u \right), u\equiv x-\overset{̿}{x}\left( t \right)$,

$$i\left( u \right)=AV\delta\left( u \right)$$

$$r\left( -u \right)=\left\{ \begin{matrix} A \exp\left[ -AR_{e}\int_{0}^{u} du^{'}K\left( u^{'} \right) \right] u>0 (S1) \\ 0 u<0 \end{matrix} \right.$$

Here $V=d\overset{̿}{x}/dt=t$ is the substitution rate defined as the number of new mutations per unit time, $\delta\left( u \right)$ is a function with unit integral, which is very narrow compared to $K\left( u \right)$due to a small speed of the wave and Fisher Fundamental Theorem, which states that the substitution rate is proportional to the variance of coordinate. The exact shape of $i\left( u \right)$ was analyzed elsewhere using multi-locus models including stochastic factors, such as mutation and random drift [1-4]. In what follows below, we will not need the explicit expression for $i\left( u \right)$.

The factor $AV$ before delta-function in Eq. S1 comes from the boundary condition for $r(u)$ at $u=0$ obtained from Eqs. (1), as described previously [5]. Normalization factor $A$ in Eq. (S1) is found from the normalization condition $\int_{-\infty}^{0} du r\left( u \right)\approx1$, which assumes that the total fraction of infected individuals is small, as given by $f_{\inf}=AV\ll1$. If that inequality does not hold, there will be negative corrections to $A$, $V$, and $f_{\inf}$.

In order to connect this analysis to the existing models of multi-locus evolution, we need to derive the adaptive landscape (fitness) defined as the number of infections per one infection (reproductive number) minus 1, as a function of antigenic coordinate. Fitness $w(x,t)$ is obtained from the second of Eqs. (1) in the main text as

$$w\left( x ,t \right)=\frac{\partial i}{i \partial t}=-1+R_{e}(x,t)\int_{-\infty}^{x} dyr\left( y,t \right)K\left( x-y \right) (S2)$$

where effective reproduction number $R_{e}$ taking into account only vaccine immunity is given by Eq. (3). In contrast, fitness $w$ takes into account both vaccine immunity via $R_{e}$ and vaccine immunity. This adaptive landscape is traveling with the wave. Because the peak of infected individuals at $u=0$is narrow, and using traveling wave $s\left( x,t \right)=s\left( u \right), u\equiv x-\overset{̿}{x}\left( t \right)$, near the peak we obtain $w(u)\approx\sigma u$, where selection coefficient $\sigma$ is the mean relative change of reproduction number per mutation

$$\sigma=\left. \frac{\partial w\left( x,t \right)}{\partial x} \right|_{u=0}=R_{e}F\left( R_{e} \right)+\frac{dR_{e}}{dx}\int_{0}^{\infty} du K\left( u \right) r\left( -u \right)= R_{e}F\left( R_{e} \right) +\frac{dR_{e}}{R_{e}dx} (S3)$$

$$F\left( R_{e} \right)\equiv\int_{0}^{\infty} dur\left( -u \right)\frac{dK\left( u \right)}{du}$$

where $r(-u)$ in the integral depends on $R_{e}$, Eq. (S1). In the first term in Eq. (S3), we used the boundary condition $K\left( 0 \right) \approx0$ (Table 1 in the main text) [6]. The integral in Eq. (S3) is calculated by the substitution of $r\left( -u \right)$ from Eq. (S1), changing the variable of integration from $u$ to $\int_{0}^{u} du^{'}K\left( u^{'} \right)$, and then using the normalization condition $\int_{-\infty}^{0} du r\left( u \right)\approx1$ and Eq. (S1). The first term in Eq. (S3) comes from the immune memory to natural infection, and the second term represents the additional immune pressure due to vaccination. The first term is decreased, and the second term is increased by vaccination. Specific expressions for terms in Eq. (S3) are derived below for different particular cases.

According to the results of multi-locus evolution models, the substitution rate $V$ is approximately linearly proportional to $\sigma$ and slowly depends of population size and mutation rate [1-4]. The total fraction of infected individuals is obtained by integrating Eq. (S1)

$$f_{\inf}=AV\approx f_{\inf}^{0}\frac{A}{A_{0}}\frac{\sigma}{\sigma_{0}} (S4)$$

where $A$is normalization factor in (S1) and index 0 stands for the respective values in the absence of vaccine, $c_{z}=0$. Below we evaluate $\sigma/\sigma_{0}$ and $f_{\inf}/f_{\inf}^{0}$ for two different cases: with the overlap of vaccination effects and without.

**Scenario 1: Last-vaccination (Fig 3).** Consider a scenario, when only the last vaccine is important for immune memory. Formally, in Eq. (S3) for reproduction number, $K_{z}\left( x-z_{j} \right)$ is replaced with zero for all but the last term, $z_{m}\equiv-\Delta z.$ Antigenic coordinate of the last vaccine lags by distance $\Delta z$ behind the most representative strain, i.e., the peak of infected distribution $i(x)$. From Eq. (3) we get

$R_{e}\left( x,t \right)=R_{0}\left\{ 1-c_{z}\left[ 1-K_{z}\left( x+\Delta z \right) \right] \right\} (S5)$

$$\frac{{dR}_{e}\left( x,t \right)}{dx}=R_{0}c_{z}\frac{{dK}_{z}\left( x-z\left( t \right) \right)}{dx} \left( S6 \right)$$

where function $K_{z}\left( u \right)$is given by Eq. (4) of main text. Upon averaging the effective reproduction number given by Eq. (S5) over the coordinate of the infected, we have

$$\left\langle R_{e} \right\rangle=R_{0}\left[ 1-c_{z}(1-\left\langle K_{z}(x+\Delta z \right\rangle) \right] \left( S7 \right)$$

$$\left\langle K_{z}(x+\Delta z) \right\rangle=\frac{\int_{-\infty}^{\infty} dx i\left( x \right)K_{z}\left( x+\Delta z \right)}{\int_{-\infty}^{\infty} du i\left( x \right)} (S8)$$

where $i(x)$ is approximated with a Gaussian with center at 0 and variance $w^{2}$, which is calculated self-consistently as explained in *S3 Text*, and symmetry $K_{z}\left( u \right)=K_{z}\left( -u \right)$ is used. The averaged $\left\langle K_{z} \right\rangle$ depends on three parameters: maximal efficacy $e_{z}$, lag $\Delta z$, and the ratio of vaccine breadth to the infected width, $b_{z}/w$.

For the derivative of effective reproduction number, from Eq. (S6) we get

$$\left\langle\frac{dR_{e}}{dx} \right\rangle=R_{0}c_{z}\left\langle\frac{dK_{z}}{dx} \right\rangle=R_{0}c_{z}\frac{\int_{-\infty}^{\infty} dx i(x)\left\langle K_{z}^{'}(x+\Delta z) \right\rangle}{\int_{-\infty}^{\infty} dx i(x)} (S9)$$

where $K_{z}^{'}(u)=-K_{z}^{'}(-u)$ can be found analytically from Eq. (4). Here averaging $\left\langle f(x) \right\rangle$ is defined as

$$\left\langle f(x) \right\rangle=\frac{\int_{-\infty}^{\infty} dx i\left( x \right)f\left( x \right)}{\int_{-\infty}^{\infty} dx i\left( x \right)} (S10)$$

where $i(x)$ is approximated by a Gaussian with variance $w^{2}$ calculated self-consistently, as explained in *S2 Text*. To determine the effective selection coefficient, one substitutes Eqs. (S7) - (S9) into Eq. (S3). This procedure is implemented in program *vaccine_w_Dz_bz.m,* and the results are shown in Fig. 3 of the main text. Estimation of parameters from data for influenza A H2N3 is discussed in *S2 Text*.

**Scenario 2: multiple vaccination with an accumulating effect (Fig. 4A-C).** Assume now that vaccination has a lasting and overlapping effect. Vaccines are administered at a time interval $t_{v}$, i.e., at interval $\Delta z_{1}=Vt_{v}$ in antigenic coordinate, where $V$ is the substitution rate. Upon averaging the effective reproduction number in Eq. (S3) over the coordinate of the infected, as given by Eq. (S10), we have

$\left\langle R_{e} \right\rangle=R_{0}\prod_{i=1}^{\infty} \left\{ 1-c_{z}\left[ 1-\left\langle K_{z}\left( x-z_{i} \right) \right\rangle\right] \right\} (S11)$

$$\frac{d\left\langle R_{e} \right\rangle}{\left\langle R_{e} \right\rangle dx}=\sum_{i=1}^{\infty} \frac{c_{z}\left\langle K_{z}^{'}(x-z_{i}) \right\rangle}{1-c_{z}\left[ 1-\left\langle K_{z}\left( x-z_{i} \right) \right\rangle\right]} \left( S12 \right)$$

Here function $K_{z}\left( u \right)$is given by Eq. (4) of main text and vaccine locations are

$z_{i}=-\Delta z-(i-1)\Delta z_{1} (S13)$

Derivative $K_{z}^{'}(u)=-K_{z}^{'}(-u)$ can be found analytically from Eq. (4). To determine the effective selection coefficient, one averages over $x$, as defined in Eq. (S10), and then substitutes Eqs. (S11), (S12) into (S3). This procedure is implemented in program *all*_*vaccine_w_Dz_bz.m,* and the results are shown in Fig. 4A-C of the main text.

**Limit of frequent vaccination (Fig. 4D).** A particular case of Scenario 2 is the hypothetical limit, where vaccinations with a long-lasting effect are administered in small intervals $\Delta z_{1}=Vt_{v}\ll b_{z}$. Assume also that either coverage $c_{z}$ or maximal efficacy $e_{z}\equiv1-K_{z}\left( 0 \right)$ are small, so that $c_{z}e_{z}\ll1$. In this case, each term in the product in Eq. (3) for $R_{e}\left( x,t \right)$in the main text can be approximated with an exponential

$$R_{e}\left( x,t \right)\approx R_{0}\exp\left[ -c_{z}\sum_{j=1}^{m} \left( 1-K_{z}\left( x-z_{j} \right) \right) \right]\approx R_{0}\exp\left[ -\frac{c_{z}}{Vt_{v}}\int_{0}^{\infty} du \left( 1-K_{z}\left( u \right) \right) \right] (S14)$$

$$\frac{{\partial R}_{e}\left( x,t \right)}{\partial x}\approx R_{e}c_{z}\sum_{j=1}^{m} \frac{{dK}_{z}\left( x-z_{j} \right)}{dx}\approx R_{e}\frac{c_{z}}{Vt_{v}}\int_{0}^{\infty} du \frac{{dK}_{z}\left( u \right)}{du}=R_{e}\frac{c_{z}}{Vt_{v}}\left( 1-K_{z}\left( 0 \right) \right) (S15)$$

Here we approximate the sums in $z_{j}$ with an integral in a continuous variable $u$= $x-z_{j}$using condition $Vt_{v}\ll b_{z}$. The quantity $Vt_{v}$ in Eqs. (S14) and (S15) represents a small step in vaccine coordinate $z_{j}$ emerging due to this approximation. The final results in Eqs. (S14) and (S15) are approximately independent on $x,t$, because the discrete sum over vaccination coordinates is approximated with a continuous integral. The derivative in $x$ in Eq. (S15) is not zero, because it is calculated before the application of this continuous approximation and emerges from the first-order correction.

It is convenient to introduce a composite “vaccine parameter”

$$\alpha\equiv\frac{c_{z}e_{z}}{\Delta z_{1}} (S16)$$

where ${\Delta z_{1}=V}_{0}t_{v}$ is the interval between vaccinations in antigenic coordinate. The composite parameter $\alpha$ combines the vaccination frequency 1/$t_{v}$, coverage $c_{z}$, maximal efficacy $e_{z}$, and the inverse substitution rate without vaccine $V_{0}$. The last quantity was previously expressed [5] in terms of basic reproduction number $R_{0}$, normalized transmission rate $K(x-y)$, mutation rate per genome, and infected population size using standard multi-locus models [1-4]. As demonstrated below, the final answer also depends on the second composite parameter

$$\gamma\equiv\frac{1}{e_{z}}\int_{0}^{\infty} du \left( 1-K_{z}\left( u \right) \right) (S17)$$

Parameter $\gamma$ can be related to the parameters of the S-shaped transmission function interpolated to data on influenza virus [6] given by Eq. (8) of the main text

$$K_{z}\left( u \right)=\frac{1}{1+\frac{e_{z}}{1-e_{z}}\exp\left( -\frac{|u|}{b_{z}} \right)} (S18)$$

with efficacy $e_{z}=0.4.$ Substituting Eq. (S18) into (S17) and integrating, one obtains

$$\gamma=b_{z}\frac{log\frac{1}{1-e_{z}}}{e_{z}}$$

In the new notation, Eqs. (S14) and (S15) take a form

$$R_{e}=R_{0}e^{-\alpha\gamma\frac{V_{0}}{V}} (S19)$$

$$\frac{{\partial R}_{e}}{\partial x}=R_{e}\alpha\frac{V_{0}}{V} (S20)$$

where the average substitution rate in epitopes $V$ is unknown at this point.

Substituting $\frac{dR_{e}}{dx}$ from Eq. (S20) into (S3) yields selection coefficient

$$\sigma=R_{e}F\left( R_{e} \right)+ \alpha\frac{V_{0}}{V} (S21)$$

The first term in selection coefficient $\sigma$ comes from the immunity to natural infection, and the second term comes from vaccine. Effective reproduction number $R_{e}$ in Eq. (S20) depends on substitution rate $V$, as given by Eq. (S15), and $F\left( R_{e} \right)$ is defined in Eqs. (S3) and (S1).

Let $X\equiv\frac{V}{V_{0}}\approx\frac{\sigma}{\sigma_{0}}$ be the fractional change in the substitution rate (selection coefficient) due to the presence of vaccination. In this notation, Eq. (S21) represents an equation for $X$

$$X=\frac{R_{e}F\left( R_{e} \right)+\alpha/X}{R_{0}F\left( R_{0} \right)} (S22)$$

$$R_{e}=R_{0}e^{-\frac{\alpha\gamma}{X}} (S23)$$

where subscript 0 stands for the values in the absence of vaccine ($c_{z}=\alpha=0$). Note that $R_{e}$ depends on parameter $X$.

Solving Eq. (S22) for $X$ numerically, one obtains the relative changes due to vaccination in the substitution rate $X$ and in infection incidence. The results obtained with program *vaccine.m* are plotted as a function of vaccine intensity parameter $\alpha$ for several values of $b_{z}$ (Fig. 4D of the main text).

1.	Rouzine IM, Wakeley J, Coffin JM. The solitary wave of asexual evolution. Proc Natl Acad Sci U S A. 2003;100(2):587-92. Epub 2003/01/15. doi: 10.1073/pnas.242719299. PubMed PMID: 12525686; PubMed Central PMCID: PMCPMC141040.

2.	Rouzine IM, Coffin JM. Evolution of human immunodeficiency virus under selection and weak recombination. Genetics. 2005;170(1):7-18. Epub 2005/03/04. doi: 10.1534/genetics.104.029926. PubMed PMID: 15744057; PubMed Central PMCID: PMCPMC1449738.

3.	Rouzine IM, Brunet E, Wilke CO. The traveling-wave approach to asexual evolution: Muller's ratchet and speed of adaptation. Theor Popul Biol. 2008;73(1):24-46. Epub 2007/11/21. doi: 10.1016/j.tpb.2007.10.004. PubMed PMID: 18023832; PubMed Central PMCID: PMCPMC2246079.

4.	Good BH, Rouzine IM, Balick DJ, Hallatschek O, Desai MM. Distribution of fixed beneficial mutations and the rate of adaptation in asexual populations. Proc Natl Acad Sci U S A. 2012;109(13):4950-5. Epub 2012/03/01. doi: 10.1073/pnas.1119910109. PubMed PMID: 22371564; PubMed Central PMCID: PMCPMC3323973.

5.	Rouzine IM, Rozhnova G. Antigenic evolution of viruses in host populations. PLoS Pathog. 2018;14(9):e1007291. Epub 2018/09/13. doi: 10.1371/journal.ppat.1007291. PubMed PMID: 30208108; PubMed Central PMCID: PMCPMC6173453.

6.	Willemsen MS, Rozhnova G. Impact of vaccination on the speed of antigenic evolution. medRxiv. 2026. doi: 10.64898/2026.03.10.26347605.
