# Supplementary material for: Vaccination accelerates the race of influenza virus against collective immunity
Source: PLoS Pathog. 2026 Oct 12;22(10):e1014311. doi: 10.1371/journal.ppat.1014311 (PMC13705590; doi:10.1371/journal.ppat.1014311)
Supplement: Supplementary file 6 [file ppat.1014311.s002.doc]

**S2 Text. Multi-locus models and fitting data on influenza**

In this section, we discuss the existing results on multi-locus models with various distributions of selection coefficients. We compare these results to influenza data on the speed and the time to most recent common ancestor to estimate the mutation rate and the width of cross-reactivity function $K(u)$ for influenza virus.

For influenza A H1N1 and H2N3 strains, average reproduction number is $R_{0}=1.5$ and 1.8, respectively, the substitution rate in epitopes is $V=2.3$ and 2.6 amino acid per year, and the time to the most recent ancestors $T_{MRCA}=4.6$ and $3.0$ years [1, 2]. Assuming 5 days for both transmission and recovery cycle, these numbers translate into $V$= $0.031$and $0.036$ per cycle, and $T_{MRCA}=336$ and $220$ cycles, respectively.

**Time to the most recent common ancestor.** According to multi-locus models [3, 4], the time to the most recent ancestor $T_{MRCA}$ is related to the adaptation rate defined as as the derivative of average fitness in time. It is given by $v \equiv s_{\mathrm{eff}} V$, where $s_{\mathrm{eff}}$ is the most probable selection coefficient of fixed mutation, and $V$ is the aminoacid substitution rate

$$T_{MRCA}=\xi\sqrt{\frac{2 log(N_{\inf} \sigma)}{v}} (S24)$$

Here $N_{\inf}\equiv Nf_{\inf}$ is the infected population number and $\xi$ is a numerical factor on the order of unit (see below). The fraction of infected individual, $f_{\inf}=AV\ll1,$ is proportional to normalization coefficient $A$ of the recovered individual density in Eq. (S1) in *S1 Text*. The value of $\sigma$ is the average selection coefficient given by Eq. (S3) in the absence of vaccine, i.e., at $R_{e}=R_{0}$and the absence of the 2^nd^ term. The transmission function $K(u)$ is given by Eq. (2) of the main text, and $r(-u)$ by Eq. (S1).

In the present work, Eq. (S24) was tested by Monte-Carlo simulation using a standard Wright-Fisher algorithm for haploid asexual population developed in [5] for the more general case with recombination (programs *scaling_genealogy.m* and *recomb_train.m*). Fixed parameters were $N_{\inf}$ and the beneficial mutation rate per genome per generation, $U_{b}$. Four forms of the distribution of selection coefficient $s$ across sites $\rho(s)$ were considered, all with average selection coefficient $\left\langle s \right\rangle=\sigma$, as follows:

1. Fixed selection coefficient $\rho\left( s \right)=\delta(s-\sigma)$
2. Exponential distribution $\rho\left( s \right)=(1/\sigma)e^{-s/\sigma}, s > 0$
3. Half-Gaussian distribution $\rho\left( s \right)=(2/\pi\sigma)e^{-s^{2}/(\pi\sigma^{2})}$, $s > 0$
4. Quasi–uniform distribution $\rho\left( s \right)=1/(2\sigma)$ in the interval $s\in\left[ 0, 2\sigma\right]$, as given by

$$\rho\left( s \right)=\frac{1}{s_{0}\Gamma\left( 1+\frac{1}{\beta} \right)}e^{-\left( \frac{s}{s_{0}} \right)^{\beta}}, s > 0$$

where average $s$ equals $\sigma$, if $s_{0}$is chosen to be

$$s_{0}\equiv2\sigma\frac{\Gamma\left( 1+\frac{1}{\beta} \right)}{\Gamma\left( 1+\frac{2}{\beta} \right)}$$

Below we assume $\beta\gg1.$ In all cases, Eq. (S24) was confirmed to be accurate in a broad range with $\xi\approx3,$ provided $U_{b}$ $\leq$ ${10}^{-2} (Fig S1)$. At $U_{b}={10}^{-1},$ the value $\xi\approx4$ was interpolated (Fig S1).

**Fitting the speed in the existing analytic models.** The general expressions for the adaptation rate $v$, substitution rate $V,$ the most probable fixed selection coefficient $s^{*}$, the lead of distribution $x_{c}$, and the lead in the mutation number $x_{0}$ in terms of model parameters were obtained, for the case of fixed selection coefficient, in [6] and, for the case of variable $s$, in [7].

*Case 1: Fixed selection coefficient:* The case was analyzed in a semi-deterministic approach of traveling wave, with a correction for discreteness of allele number [6]. From eqs. 39 and 51 in [6] we get

$$V=2\sigma{\log\left[ N_{\inf}\sqrt{\frac{\sigma^{3}U_{b}}{V^{2}\log\frac{V}{U_{b}}}} \right]}/\left( \log^{2}\frac{V}{eU_{b}}+1 \right), V\gg\sigma, U_{b} (S25)$$

$$x_{0}=\frac{V}{\sigma}\log\frac{V}{eU_{b}}$$

where $x_{0}$ is the average lead, i.e., the excess of beneficial mutations in the best-fit genome compared to the average genome. The arguments of both logarithms are supposed to be much larger than 1. The variance of allele number $\mathrm{Var}\left[ x \right]\equiv w^{2}$is given by$w^{2}=V/\sigma$, which is a case of Fisher theorem. The adaptation rate is given by $v=V\sigma$. The accuracy of these expressions has been confirmed by Monte Carlo simulation [6].

*Case 2: Exponential distribution of* $s$*:* The case $\rho\left( s \right)=(1/\sigma)e^{-s/\sigma}$ was analyzed by a branching method calculating the allele fixation probability and two other approaches, all three based on the approximation of the genetic background of a smoothly moving fitness distribution [7]. Eqs. 12-14 from [7] represent а system of transcendental equations for the average fitness increase per generation (adaptation rate) $v$ and the most probable selection coefficient of established mutation $s^{*}$, as well as the threshold in fitness where $x_{c}=s^{*}+\frac{v}{\sigma}$ , above which clonal interference is no longer important [7]. The accuracy of the final results for $s^{*}$ and $v$ (not shown here) was confirmed by Monte Carlo simulation previously [7]. In terms of these results, one obtains substitution rate $V$, variance $w^{2}$, and lead$x_{0}$ in allele number

$$V=\frac{v}{s^{*}} , w^{2}=\frac{V}{s^{*}}, x_{0}=\frac{x_{c}}{s^{*}},$$

*Case 3: Half-Gaussian distribution of s:* The same approximation was used to analyze the case of arbitrary distribution of selection coefficient [7]. For the half Gaussian distribution $\rho\left( s \right)=(2/\pi\sigma)e^{-s^{2}/(\pi\sigma^{2})}$, from eqs. 17-19 in [7] we get

$$v=\frac{\pi\sigma^{2}}{2}\left( x_{0}-1 \right), x_{0}\equiv\frac{x_{c}}{s^{*}}$$

The values of $x_{0}$ and $\zeta^{*}$ are found by solving together the following system of equations

$$x_{0}=1+\epsilon+\sqrt{\epsilon+\epsilon^{2}} , \epsilon\equiv\frac{\log\left[ \frac{2N_{\inf} s^{*}U_{b}}{\pi\sigma}\frac{{x_{0}}^{\frac{3}{2}}}{x_{0}-1} \right]}{\log\left( \frac{s^{*}}{U_{b}}\sqrt{\frac{x_{0}-1}{x_{0}}} \right)}$$

$$s^{*}=\sigma\sqrt{\frac{\pi{(x}_{0}-1)}{2x_{0}-1}\log\left( \frac{s^{*}}{U_{b}}\sqrt{\frac{x_{0}-1}{x_{0}}} \right)}$$

These equations are solved numerically by iterations, which converge fast. Substitution rate and allele number variance are $V=v/s^{*}$and $w^{2}=V/s^{*}$. Unfortunately, these analytic results perform poorly when compared to Monte Carlo simulation (not shown). Hence, this case will not be considered further.

*Case 4: Quasi-uniformly distributed s*. The approach in [7] works better in the case of a sharply decaying distribution

$$\rho\left( s \right)=\frac{1}{s_{0}\Gamma\left( 1+\frac{1}{\beta} \right)}e^{-\left( \frac{s}{s_{0}} \right)^{\beta}}, s > 0$$

where $\beta\gg1$. Average $s$ equals $\sigma$, if $s_{0}$is chosen to be

$$s_{0}\equiv2\sigma\frac{\Gamma\left( 1+\frac{1}{\beta} \right)}{\Gamma\left( 1+\frac{2}{\beta} \right)}$$

Assuming $\beta\gg1$ makes $\rho\left( s \right)$ similar to a uniform distribution in the interval $s\in\left[ 0, 2\sigma\right].$ In Monte-Carlo simulation (below), the uniform distribution is used, but the analytic approach in [7] is amenable only to a smooth dependence on $s$. The results [7] have an approximate form

$$v\approx\frac{2s_{0}^{2}\log\left[ Ns_{0}\log^{\frac{1}{2}}\left( Ns_{0} \right) \right]}{\log^{2}\left[ \frac{\sqrt{\beta}s_{0}}{U_{b}}\log^{\frac{1}{2}}\left( \frac{\sqrt{\beta}s_{0}}{U_{b}} \right) \right]}\left[ 1+\frac{\log\left( \frac{\sqrt{\beta}s_{0}}{U_{b}} \right)}{4\log\left( Ns_{0} \right)} \right]^{-2}$$

$$s^{*}\approx s_{0}\left[ \frac{\sqrt{2}}{\beta}\log\frac{s_{0}}{U_{b}} \right]^{\frac{1}{\beta-1}} (S26)$$

The first formula in Eqs. (S26) is eq. 21 in [7]. The second formula is eq. 14 in [8] derived in that work from eqs. 18-21 in [7].

Eq. S26 includes parameter $s_{0}$, which is proportional to the average selection coefficient $\sigma$, as shown above. In turn, the average selection coefficient $\sigma$ is is expressed in terms of $R_{e}$, as given by Eq. 8 (Eq. S3). See S1 Text.

Comparison with Monte Carlo simulation for a representative parameter set and $\beta=10$ performed with the use of programs *testS3andS5.m* and *recomb_train2.m* demonstrates a fair accuracy of both expressions (*Fig. S2*). Independently on analytic Eqs. (S26), the Fisher relation $w^{2}=V/s^{*}$ for Monte Carlo results holds with a good accuracy (*Fig. S2*), which confirms that a population with distributed selection coefficient $s$ can be described [7] in terms of a single effective $s^{*}$.

Beneficial mutation rate $U_{b}$ and the breadth of natural immunity $b_{y}$ were adjusted to fit the experimental values of $V$ and $T_{MRCA}$ for the case of fixed $s$, the exponential distribution, and the uniform distribution of $s$. The fitting procedure is illustrated for the uniform distribution of $s$ used in the main text (Fig. S3). The fitting error to data on influenza A H3N2 strain, $T_{MRCA}$ and $V$ relative to the observed values (3 year and 2.6 aa/year), the annual incidence of infection, average selection coefficient $\sigma$, the effective selection coefficient $s^{*},$ and the peak variance $w^{2}$ are plotted as a function of $U_{b}$ and $b_{y}$ (Fig. S3). Calculations were performed using programs *main_fitting.m* and *call_fitting.m*.

The best-fit values of $U_{b}$ and $b_{y}$ are sensitive to the form of the distribution of $s$ that remains unknown. The exponential distribution of $s$ predicts an unrealistically large immune breadth, $b_{y}=18.7$, which corresponds to the half-width of transmission function equal to 70 amino acid substitutions: $K(70)=0.5$. This is way too large, given the limited size of the five antibody epitopes that together comprise no more than 60 amino acids. (Please note that, at very large $U_{b}$and $b_{y},$ iterations to solve eqs. 13, 14 in [7] diverge, and one has to use the method of division by two.) Note that, even though the exponential form of DFE is often observed for viruses and bacteria evolving under purifying selection, which observation has been explained by an evolution model [9], in the Red Queen regime considered here, adaptive landscape moves with the solitary wave, there is no best-fit sequence, and the theory predicting an exponential DFE does not apply [8].

The fixed-$s$ scenario, on the other hand, predicts a very small breadth, $b_{y}=1.7$, i.e. $a=7$, and $U_{b}=3{10}^{-5}.$ While this is close to the value $b_{y}=2$observed in hemagglutinin binding assay where an exponential distribution was observed [10], the fixed–s scenario is also not very realistic.

The case of uniform distribution, intermediate between the two above cases, gives the most realistic results: $U_{b}={10}^{-3}$ and ${10}^{-2.5}$and $b_{y}=4.1$ and $7.2$ for influenza A H3N2 and H1N1, respectively (Fig. S3). The breadth $b_{y}=4.1$ corresponds to the half-width of function $K(x)$, defined as $K(a)=1/2$, equal to $a=$15, which is similar to the previous estimates [1, 8]. The predicted annual incidence of infection is in the realistic range 5-10% (Fig. S3).

1.	Bedford T, Riley S, Barr IG, Broor S, Chadha M, Cox NJ, et al. Global circulation patterns of seasonal influenza viruses vary with antigenic drift. Nature. 2015;523(7559):217-20. Epub 2015/06/09. doi: 10.1038/nature14460. PubMed PMID: 26053121; PubMed Central PMCID: PMCPMC4499780.

2.	Biggerstaff M, Cauchemez S, Reed C, Gambhir M, Finelli L. Estimates of the reproduction number for seasonal, pandemic, and zoonotic influenza: a systematic review of the literature. BMC Infect Dis. 2014;14:480. Epub 20140904. doi: 10.1186/1471-2334-14-480. PubMed PMID: 25186370; PubMed Central PMCID: PMCPMC4169819.

3.	Desai MM, Walczak AM, Fisher DS. Genetic diversity and the structure of genealogies in rapidly adapting populations. Genetics. 2013;193(2):565-85. Epub 2012/12/12. doi: 10.1534/genetics.112.147157. PubMed PMID: 23222656; PubMed Central PMCID: PMCPMC3567745.

4.	Neher RA, Hallatschek O. Genealogies of rapidly adapting populations. Proc Natl Acad Sci U S A. 2013;110(2):437-42. Epub 2012/12/28. doi: 10.1073/pnas.1213113110. PubMed PMID: 23269838; PubMed Central PMCID: PMCPMC3545819.

5.	Batorsky R, Kearney MF, Palmer SE, Maldarelli F, Rouzine IM, Coffin JM. Estimate of effective recombination rate and average selection coefficient for HIV in chronic infection. Proc Natl Acad Sci U S A. 2011;108(14):5661-6. Epub 2011/03/26. doi: 10.1073/pnas.1102036108. PubMed PMID: 21436045; PubMed Central PMCID: PMCPMC3078368.

6.	Rouzine IM, Brunet E, Wilke CO. The traveling-wave approach to asexual evolution: Muller's ratchet and speed of adaptation. Theor Popul Biol. 2008;73(1):24-46. Epub 2007/11/21. doi: 10.1016/j.tpb.2007.10.004. PubMed PMID: 18023832; PubMed Central PMCID: PMCPMC2246079.

7.	Good BH, Rouzine IM, Balick DJ, Hallatschek O, Desai MM. Distribution of fixed beneficial mutations and the rate of adaptation in asexual populations. Proc Natl Acad Sci U S A. 2012;109(13):4950-5. Epub 2012/03/01. doi: 10.1073/pnas.1119910109. PubMed PMID: 22371564; PubMed Central PMCID: PMCPMC3323973.

8.	Rouzine IM, Rozhnova G. Antigenic evolution of viruses in host populations. PLoS Pathog. 2018;14(9):e1007291. Epub 2018/09/13. doi: 10.1371/journal.ppat.1007291. PubMed PMID: 30208108; PubMed Central PMCID: PMCPMC6173453.

9.	Barlukova A, Rouzine IM. The evolutionary origin of the universal distribution of mutation fitness effect. PLoS Comput Biol. 2021;17(3):e1008822. Epub 2021/03/09. doi: 10.1371/journal.pcbi.1008822. PubMed PMID: 33684109; PubMed Central PMCID: PMCPMC7971868.

10.	Willemsen MS, Rozhnova G. Impact of vaccination on the speed of antigenic evolution. medRxiv. 2026. doi: 10.64898/2026.03.10.26347605.
