# Supplementary material for: Vaccination accelerates the race of influenza virus against collective immunity
Source: PLoS Pathog. 2026 Oct 12;22(10):e1014311. doi: 10.1371/journal.ppat.1014311 (PMC13705590; doi:10.1371/journal.ppat.1014311)
Supplement: Supplementary file 7 [file ppat.1014311.s003.doc]

**S3 Text.** **Viral extinction and the critical point**

As mentioned in main text, the model predicts viral extinction in the parameter region, where $R_{e}\leq1$ (Fig. 4). At the point $R_{e}=1$, the incidence vanishes, as illustrated in Fig. S4. The result is expected, because the virus cannot spread at basic reproduction number $R_{e}$equal or less than unit. Formally, the density of recovered individuals $r(-u, R)$ given by Eq. (S1) cannot be normalized at $R_{e}\leq1$.When $R_{e}$ is approaching unit, function $r(-u,R)$ becomes broader and broader, and, at $R_{e}=1$, “spills out” into an infinite range of $u$ (Fig. S4). At this point, the natural-immunity component of selection coefficient given by the first term in the right-hand side of Eq. (S7) vanishes. It is important, therefore, to allow in numerical code for a very broad range of antigenic coordinate, up to 3000, otherwise, delocalization will be smeared out.

The need for a very broad range near extinction point is the artifact of a 1D model. In real life, delocalization occurs in a multi-dimensional space, which allows for using a much smaller range of antigenic coordinate. The 1D model is justified, because a quasi-1D path is formed spontaneously in multi-dimensional space [1, 2]. However, because the model disregards the existence of other dimensions, it creates this issue with delocalization.

Influenza and SARS-CoV-2 genomes are observed to have hundreds of genetically diverse, simultaneously evolving sites (see references in Introduction). This is because these viruses evolve deeply into multi-locus regime, $N_{\inf}U_{b} \sim100-1000$. Only very close to the predicted extinction point, where the infection incidence drops by more than two orders of magnitude, the opposite condition will apply, $N_{\inf}U_{b}\ll1,$mutations will emerge one at a time, and vial evolution will be described by the one-locus models.

1.	Bedford T, Rambaut A, Pascual M. Canalization of the evolutionary trajectory of the human influenza virus. BMC Biol. 2012;10:38. Epub 2012/05/02. doi: 10.1186/1741-7007-10-38. PubMed PMID: 22546494; PubMed Central PMCID: PMCPMC3373370.

2.	Rouzine IM, Rozhnova G. Antigenic evolution of viruses in host populations. PLoS Pathog. 2018;14(9):e1007291. Epub 2018/09/13. doi: 10.1371/journal.ppat.1007291. PubMed PMID: 30208108; PubMed Central PMCID: PMCPMC6173453.
