# Supplementary material for: Vaccination accelerates the race of influenza virus against collective immunity
Source: PLoS Pathog. 2026 Oct 12;22(10):e1014311. doi: 10.1371/journal.ppat.1014311 (PMC13705590; doi:10.1371/journal.ppat.1014311)
Supplement: Supplementary file 8 [file ppat.1014311.s004.doc]

**S4 Text.** **Approximations and simplifications**

The model in Fig. 1 makes several simplifications and approximations, as follows.

Location of epitopes is assumed to be the same across individuals. This assumption is appropriate for the neutralizing B-cell response in many viruses, where the location of antibody binding sites is almost fixed in the envelope protein. The assumption does not apply to T-cell response, where epitope location varies broadly between individuals according to their HLA subtypes.

Cost of escape mutation is assumed to be negligible compared to the gain due to antigenic escape. This assumption is appropriate for the neutralizing B-cell response in the cited viruses. Their surface proteins contain 4-5 protrusions that evolved to serve as decoys for neutralizing antibodies and, hence, can vary easily, in contrast to the receptor-binding regions that are not accessible to antibodies and are conserved. The approach, again, would not work for T-cell epitopes, where an average mutation cost is high [1, 2]. In that case, the tradeoff between mutation gain and mutation cost determines the order of escape mutations and limits the number of escape events [3]. Some escape mutations in SARS-CoV-2 protein Spike incur a finite cost, which is why they are assisted by compensatory mutations [4-7], so that this assumption needs to be tested experimentally in the future.

Antigenic coordinate is assumed to be one-dimensional, because modeling and observation demonstrate that a quasi 1D path is formed by virus variants in multidimensional antigenic space [8-10]. Hemaglutinin includes 60 aminoacids, each can mutate to 10 checmically distinct variants, which implies the space dimension of several hundred. The quasi-one-dimensionality prediction of the models agrees with the phylogenetic studies of influenza virus [11, 12] and with the fact that large variants of SARS-CoV-2 co-exist only transiently [13-16].

The immune memory of infections that happened before the last infection of individual is neglected. This approximation is valid due to a high dimensionality of genetic space, within which the one-dimensional path of antigenic escape is formed [8, 10]. It is highly unlikely that the path, which is also self-repelling, can cross itself. Hence, old memory cells are antigenically-remote and not relevant for protection against the circulating virus variants.

Infections of individuals located back in antigenic coordinate are neglected to prevent the artifact of mirror wave in antigenic space. This approximation introduces a minor error in the results [10].

B-cell memory cells left by an infection with a virus or a vaccine have a sequence of B cell receptor (antibody) that varies around an average sequence. This variation is not neglected, but taken into account in the model by the breadth of normalized transmission functions $K\left( u \right)$and $K_{z}(u)$ determined from fitting data [17] (*S2 Text*). The averaged-out variation of the level of memory cells between patients is also included in functions $K(u)$ and $K_{z}(u)$.

The fraction of infected individuals is assumed to be small, which holds for influenza virus and SARS-CoV-2 even during the seasonal outbreaks.

Natural population turnover is neglected, because the lifespan is much longer than the relevant time scales: the interval between infections and the time to the most recent common ancestor.

Seasonal oscillations have a small effect on results, because periodic seasonal effects average out over the long-term stationary process.

The effect of bias of immune system after the exposure to the first strain is known in immunology as "original antigenic sin" or "immune imprinting". However, it does not matter for the model, because the only parameter of the immune system that enters the model is the crossimmunity function $K(x),$ which is averaged over different adults that have different age and imprinting of different strains. The same function is also averaged over the individual's level of memory cells left after infection or the individual's breadth of response. And its form and breadth determined from fitting data anyway.

The model assumes that vaccine-derived immunity is independent from natural immunity. In principle, this condition could be violated due to the fact that infected people are unvaccinated. However, the annual incidence of influenza usually does not exceed 20%-40%. With 5 days per infection, this corresponds to 0.3-0.6% individuals infected at any one time, or 0.6-1.2% if the seasonal nature of epidemic is taken into account (sprint and autumn). Hence the effect of correlation creates a neglible error of ~ 1%. Significant correlations between the two kinds of immunity could arise from the nonuniform patterns of risk behavior. People who are reckless are both at a higher risk of getting infected and less likely to get vaccinated, which could create a negative correlation between two types of immunity. The focus of the present manuscript is a simple model of a population with uniform human behavior and random vaccination.

An influenza or coronavirus infection is cleared within several days leaving behind memory T cells and B cells, which reach a plateau in a few days or weeks. Note that the renfection of the same person by virus occur several years apart, i.e., much later after the inital drop. There is theoretical possibility that long-term waning of immunity could contribute to virus persistence. Some SIR models considered immune waning without strain-structuring the population [18-20]. However, the author is not aware of any clinical evidence that immunity against influenza wanes beyond the first several weeks. Moreover, the fast speed of evolution disagrees with waning immunity but is consistent with antigenic escape. If immunity would wane, virus would not have to evolve at such an enormous speed, ${\sim10}^{-3}$/site/year. This is why the modern models of influenza persistence cited in Discussion neglect waning.

1.	Ganusov VV, Goonetilleke N, Liu MK, Ferrari G, Shaw GM, McMichael AJ, et al. Fitness costs and diversity of the cytotoxic T lymphocyte (CTL) response determine the rate of CTL escape during acute and chronic phases of HIV infection. J Virol. 2011;85(20):10518-28. Epub 2011/08/13. doi: 10.1128/JVI.00655-11. PubMed PMID: 21835793; PubMed Central PMCID: PMCPMC3187476.

2.	Ganusov VV, De Boer RJ. Estimating costs and benefits of CTL escape mutations in SIV/HIV infection. PLoS Comput Biol. 2006;2(3):e24. Epub 20060331. doi: 10.1371/journal.pcbi.0020024. PubMed PMID: 16604188; PubMed Central PMCID: PMCPMC1420660.

3.	Batorsky R, Sergeev RA, Rouzine IM. The route of HIV escape from immune response targeting multiple sites is determined by the cost-benefit tradeoff of escape mutations. PLoS Comput Biol. 2014;10(10):e1003878. Epub 2014/10/31. doi: 10.1371/journal.pcbi.1003878. PubMed PMID: 25356981; PubMed Central PMCID: PMCPMC4214571.

4.	Carabelli AM, Peacock TP, Thorne LG, Harvey WT, Hughes J, Consortium C-GU, et al. SARS-CoV-2 variant biology: immune escape, transmission and fitness. Nat Rev Microbiol. 2023;21(3):162-77. Epub 20230118. doi: 10.1038/s41579-022-00841-7. PubMed PMID: 36653446; PubMed Central PMCID: PMCPMC9847462.

5.	Moulana A, Dupic T, Phillips AM, Desai MM. Genotype-phenotype landscapes for immune-pathogen coevolution. Trends Immunol. 2023;44(5):384-96. Epub 20230404. doi: 10.1016/j.it.2023.03.006. PubMed PMID: 37024340; PubMed Central PMCID: PMCPMC10147585.

6.	Phillips AM, Maurer DP, Brooks C, Dupic T, Schmidt AG, Desai MM. Hierarchical sequence-affinity landscapes shape the evolution of breadth in an anti-influenza receptor binding site antibody. Elife. 2023;12. Epub 20230110. doi: 10.7554/eLife.83628. PubMed PMID: 36625542; PubMed Central PMCID: PMCPMC9995116.

7.	Witte L, Baharani VA, Schmidt F, Wang Z, Cho A, Raspe R, et al. Epistasis lowers the genetic barrier to SARS-CoV-2 neutralizing antibody escape. Nat Commun. 2023;14(1):302. Epub 20230119. doi: 10.1038/s41467-023-35927-0. PubMed PMID: 36653360; PubMed Central PMCID: PMCPMC9849103.

8.	Bedford T, Rambaut A, Pascual M. Canalization of the evolutionary trajectory of the human influenza virus. BMC Biol. 2012;10:38. Epub 2012/05/02. doi: 10.1186/1741-7007-10-38. PubMed PMID: 22546494; PubMed Central PMCID: PMCPMC3373370.

9.	Smith DJ, Lapedes AS, de Jong JC, Bestebroer TM, Rimmelzwaan GF, Osterhaus AD, et al. Mapping the antigenic and genetic evolution of influenza virus. Science. 2004;305(5682):371-6. Epub 2004/06/26. doi: 10.1126/science.1097211. PubMed PMID: 15218094.

10.	Rouzine IM, Rozhnova G. Antigenic evolution of viruses in host populations. PLoS Pathog. 2018;14(9):e1007291. Epub 2018/09/13. doi: 10.1371/journal.ppat.1007291. PubMed PMID: 30208108; PubMed Central PMCID: PMCPMC6173453.

11.	Bedford T, Riley S, Barr IG, Broor S, Chadha M, Cox NJ, et al. Global circulation patterns of seasonal influenza viruses vary with antigenic drift. Nature. 2015;523(7559):217-20. Epub 2015/06/09. doi: 10.1038/nature14460. PubMed PMID: 26053121; PubMed Central PMCID: PMCPMC4499780.

12.	Bedford T, Suchard MA, Lemey P, Dudas G, Gregory V, Hay AJ, et al. Integrating influenza antigenic dynamics with molecular evolution. Elife. 2014;3:e01914. Epub 2014/02/06. doi: 10.7554/eLife.01914. PubMed PMID: 24497547; PubMed Central PMCID: PMCPMC3909918.

13.	Volz E, Mishra S, Chand M, Barrett JC, Johnson R, Geidelberg L, et al. Assessing transmissibility of SARS-CoV-2 lineage B.1.1.7 in England. Nature. 2021;593(7858):266-9. Epub 2021/03/27. doi: 10.1038/s41586-021-03470-x. PubMed PMID: 33767447.

14.	Davies NG, Abbott S, Barnard RC, Jarvis CI, Kucharski AJ, Munday JD, et al. Estimated transmissibility and impact of SARS-CoV-2 lineage B.1.1.7 in England. Science. 2021;372(6538): eabg3055. Epub 2021/03/05. doi: 10.1126/science.abg3055. PubMed PMID: 33658326; PubMed Central PMCID: PMCPMC8128288.

15.	Campbell F, Archer B, Laurenson-Schafer H, Jinnai Y, Konings F, Batra N, et al. Increased transmissibility and global spread of SARS-CoV-2 variants of concern as at June 2021. Euro Surveill. 2021;26(24):pii=2100509. Epub 2021/06/19. doi: 10.2807/1560-7917.ES.2021.26.24.2100509. PubMed PMID: 34142653; PubMed Central PMCID: PMCPMC8212592.

16.	Graham MS, Sudre CH, May A, Antonelli M, Murray B, Varsavsky T, et al. Changes in symptomatology, reinfection, and transmissibility associated with the SARS-CoV-2 variant B.1.1.7: an ecological study. Lancet Public Health. 2021;6(5):e335-e45. Epub 2021/04/16. doi: 10.1016/S2468-2667(21)00055-4. PubMed PMID: 33857453; PubMed Central PMCID: PMCPMC8041365

17.	Willemsen MS, Rozhnova G. Impact of vaccination on the speed of antigenic evolution. medRxiv. 2026. doi: 10.64898/2026.03.10.26347605.

18.	Liu D, Sopasakis A. A combined neural ODE-Bayesian optimization approach to resolve dynamics and estimate parameters for a modified SIR model with immune memory. Heliyon. 2024;10(19):e38276. Epub 20240924. doi: 10.1016/j.heliyon.2024.e38276. PubMed PMID: 39391478; PubMed Central PMCID: PMCPMC11466598.

19.	Taylor ML, Carr TW. An SIR epidemic model with partial temporary immunity modeled with delay. J Math Biol. 2009;59(6):841-80. Epub 20090306. doi: 10.1007/s00285-009-0256-9. PubMed PMID: 19266170.

20.	El Khalifi M, Britton T. Extending susceptible-infectious-recovered-susceptible epidemics to allow for gradual waning of immunity. J R Soc Interface. 2023;20(206):20230042. Epub 20230913. doi: 10.1098/rsif.2023.0042. PubMed PMID: 37700711; PubMed Central PMCID: PMCPMC10498349.
