# Supplementary material for: Vaccination accelerates the race of influenza virus against collective immunity
Source: PLoS Pathog. 2026 Oct 12;22(10):e1014311. doi: 10.1371/journal.ppat.1014311 (PMC13705590; doi:10.1371/journal.ppat.1014311)
Supplement: Supplementary file 9 [file ppat.1014311.s005.doc]

**S5 Text. The equivalence to a model with a vaccinated compartment**

A related 3-compartment model with a separate vaccinated compartment was studied by Monte Carlo simulation in [1]. The cited authors also simulated the 2-compartment model with an effective reproduction number given by Eq. (3) in the last-vaccine scenario ($j=m$ only), similar to the model used in the present work. Below the 3-compartment model and the 2-compartment are shown to be equivalent in the limit of very frequent vaccination.

The deterministic part of 3-compartment model is formalized by integro-differential equations, which, in the present notation, have a form [1]

$$\frac{\partial u\left( x,t \right)}{\partial t}=i\left( x,t \right)-u\left( x,t \right)\left( \gamma+\beta\int_{x}^{\infty} dy K\left( x-y \right)i\left( y,t \right) \right)$$

$+\delta(x-x_{in})\gamma$ + $\delta(t-mt_{v})\left[ v\left( x,t \right)-c_{z}\left[ u\left( x,t \right)+v\left( x,t \right) \right] \right] (S27)$

$$\frac{\partial v\left( x, t \right)}{\partial t}=-v\left( x,t \right)\left( \gamma+ \beta\int_{x}^{\infty} {dy K}_{v}\left( x-y,x-z_{m} \right)i\left( y,t \right) \right)$$

$+\delta(t-mt_{v})\left[ -v\left( x,t \right)+c_{z}\left[ u\left( x,t \right)+v\left( x,t \right) \right] \right]$ (S28)

$\frac{\partial i(x, t)}{\partial t}=-(1+\gamma)i\left( x,t \right)+\beta i\left( x, t \right)\int_{-\infty}^{x} dy \left[ K\left( x-y \right)u\left( y,t \right)+K_{v}\left( x-y,x-z_{m} \right)v\left( y,t \right) \right]$(S29)

$$\beta\equiv R_{0}(\gamma+r)$$

$$\int_{-\infty}^{\infty} dx \left[ i\left( x, t \right)+u\left( x,t \right)+ v\left( x,t \right) \right]=1$$

$K_{v}\left( x-y, x-z \right)\equiv K\left( x-y \right)K_{z}\left( x-z \right)$	(S30)

Here $u(x,t)$ and $v(x,t)$ are the density of unvaccinated and vaccinated compartment of population, respectively, with the memory of virus with antigenic coordinate $x$, and $i\left( x, t \right)$ are the density of infected individuals infected with virus $x$. The last vaccination is administered by a pulse at time $t=mt_{v}$, where $m$ is an integer number and is $t_{v}$ is the vaccination interval, and $z_{m}=Vmt_{v}$ is the antigenic coordinate of the vaccine vector, i.e., as in Eq. (3) with term $j=m$only.

The terms in the right-hand side of Eq. (S27) describe recovery of unvaccinated, natural population decline, reinfection, introduction of new uninfected into adult population. The model assumes that each vaccination cancels the effect of the previous vaccination and sends vaccinated individuals back to the unvaccinated compartment before revaccinating their fraction $c_{z}<1$. This action is described by the terms with delta-functions in time. The terms in Eq. (S28) describe population decline and infection of vaccinated. The terms with delta-function are the same as in Eq. (S27), but with the opposite sign. The terms in Eq. (S29) describe the recovery of the infected, natural population decline, and the influx from vaccinated and unvaccinated.

Parameter $\gamma$ is the population turnover (birth and death) time, and $x_{in}$ is any arbitrary large negative coordinate, where the unvaccinated enter the population. In the present work, it is neglected, $\gamma=0$, because the time to the most recent infection with a respiratory virus is much smaller than the average life span. Hence, coefficient $\beta\equiv R_{0}.$ Function $K_{v}\left( x-y,x-z \right)$ is the normalized transmission function taking into account the natural immunity to virus variant $x$left from infection with variant $y$, $K\left( x-y \right)$, and the immunity left from vaccine $z$, $K_{z}(x-z)$. Their effect is multiplicative based on the assumption that the virus has to pass through the two sets of antibodies to infect at the systemic level, each being a binary event (“pass - no pass”) with some probability, and that the probability to escape capture by either set is statistically independent. Normalized transmission functions $K\left( x-y \right)$ and $K_{z}(x-z)$ are averaged out over the variation of memory B cell level among individual patients, as well as over their antigenic heterogeneity around the nominal antigenic coordinate $y$ and $z$. These effects are incorporated into their form estimated from data [1].

When vaccine is very frequent, exchanges between vaccinated and susceptible compartments are very frequent, and oscillations in the state variables induced by the periodic pulses are small. In this asymptotic limit, each delta-function in time in Eq. (S27-28) can be approximated with its time average $1/t_{v}$. Taking into account $\gamma=0, r=1$, and Eq. (S30), Eq. (S27-29) simplify to

$\frac{\partial u\left( x,t \right)}{\partial t}=i\left( x,t \right)-R_{0} u\left( x,t \right)\int_{x}^{\infty} K\left( x-y \right)i\left( y,t \right)dy+ \frac{1}{t_{v}}\left\{ v\left( x,t \right)-c_{z}\left[ u\left( x,t \right)+v\left( x,t \right) \right] \right\}$	(S31)

$\frac{\partial v\left( x, t \right)}{\partial t}=-R_{0}v\left( x,t \right)K_{z}\left( x-z_{m} \right)\int_{x}^{\infty} K\left( x-y \right)i\left( y,t \right)dy- \frac{1}{t_{v}}\left\{ v\left( x,t \right)-c_{z}\left[ u\left( x,t \right)+v\left( x,t \right) \right] \right\}$(S32)

$\frac{\partial i(x, t)}{\partial t}=-i\left( x,t \right)+R_{0}i\left( x, t \right)\int_{-\infty}^{x} K\left( x-y \right)\left\{ u\left( y,t \right)+K_{z}\left( x-z_{m} \right)v\left( y,t \right) \right\}dy$	(S33)

Because $1/t_{v}$ is assumed to be very large, for the solution of Eqs. (S31-S33) not to be infinite, the terms in curly brackets must be very small, so that

$$v\left( x,t \right)\approx c_{z}\left[ u\left( x,t \right)+v\left( x,t \right) \right], u\left( x,t \right)\approx{(1-c}_{z})\left[ u\left( x,t \right)+v\left( x,t \right) \right] (S34)$$

Adding together Eqs. (S31) and (S33) and substituting Eqs. (S34) into Eq. (S33), for the sum $r\left( x,t \right)\equiv u\left( x,t \right)+v\left( x,t \right)$, one arrives at Eq. (1) of the main text. The effective reproduction number $R_{e}$ is given by Eq. (3), where only the last vaccination event, $j=m$, is included.

1.	Willemsen MS, Rozhnova G. Impact of vaccination on the speed of antigenic evolution. medRxiv. 2026. doi: 10.64898/2026.03.10.26347605.
