# Supplementary material for: Vaccination accelerates the race of influenza virus against collective immunity
Source: PLoS Pathog. 2026 Oct 12;22(10):e1014311. doi: 10.1371/journal.ppat.1014311 (PMC13705590; doi:10.1371/journal.ppat.1014311)
Supplement: Peer review history

# Author comment: Author response to Decision Letter 0

**DOI:** 10.1371/journal.ppat.1014311.r001

1 Jun 2026

Attachment Submitted filename: Rebuttal.pdf

# Editor report: Decision Letter 0

**DOI:** 10.1371/journal.ppat.1014311.r002

**Authors:** Ronald Swanstrom, Peter Lidsky

15 Jun 2026

PPATHOGENS-D-26-01345

Vaccination accelerates the race of a respiratory virus against collective immunity

PLOS Pathogens

Dear Dr. Rouzine,

As requested, the manuscript is send back to you for minor updates prior to review.

Kind regards,

Peter V. Lidsky, Ph.D.

Academic Editor

PLOS Pathogens

Ronald Swanstrom

Section Editor

PLOS Pathogens

Sumita Bhaduri-McIntosh

Editor-in-Chief

PLOS Pathogens

orcid.org/0000-0003-2946-9497

Michael Malim

Editor-in-Chief

PLOS Pathogens

orcid.org/0000-0002-7699-2064

**JOURNAL REQUIREMENTS:**

1) Please provide an Author Summary. This should appear in your manuscript between the Abstract (if applicable) and the Introduction, and should be 150-200 words long. The aim should be to make your findings accessible to a wide audience that includes both scientists and non-scientists. Sample summaries can be found on our website under Submission Guidelines:

https://journals.plos.org/plospathogens/s/submission-guidelines#loc-parts-of-a-submission

2) We do not publish any copyright or trademark symbols that usually accompany proprietary names, eg ©,  ®, or TM  (e.g. next to drug or reagent names). Therefore please remove all instances of trademark/copyright symbols throughout the text, including:

- TM on page: 5 and 10.

3) Please upload all main figures as separate Figure files in .tif or .eps format. For more information about how to convert and format your figure files please see our guidelines:

https://journals.plos.org/plospathogens/s/figures

4) Please upload a copy of Figure Fig 1-4 which you refer to in your text on pages 6, 11, and 14 and 17. Or, if the figure is no longer to be included as part of the submission please remove all reference to it within the text.

5) We notice that your supplementary Figures are included in the manuscript file. Please remove them and upload them with the file type 'Supporting Information'. Please ensure that each Supporting Information file has a legend listed in the manuscript after the references list.

6) Please amend your detailed Financial Disclosure statement. This is published with the article. It must therefore be completed in full sentences and contain the exact wording you wish to be published.

1) State what role the funders took in the study. If the funders had no role in your study, please state: "The funders had no role in study design, data collection and analysis, decision to publish, or preparation of the manuscript."

2) If any authors received a salary from any of your funders, please state which authors and which funders..

If you did not receive any funding for this study, please simply state: u201cThe authors received no specific funding for this work.u201d

7) Thank you for stating "No new data have been collected. Programs are available at https://github.com/irouzine/Red-Queen." However, we were unable to access the sequence data using the information provided. Please review and update the Data Availability Statement to ensure that the underlying data are publicly accessible and that all accession numbers, links, or repository details are correct and functional.

8) We have amended your Competing Interest statement to comply with journal style. We kindly ask that you double check the statement and let us know if anything is incorrect.

9) Your current Financial Disclosure states, “I.M.R. worked within the framework of the state assignment of the Federal Agency for Scientific Organizations (FASO Russia: topic no. 075-00264-26-00).” However, the funding information provided in the submission form does not match the information reported in the Financial Disclosure statement. Please indicate by return email the full and correct funding information for your study and confirm the order in which funding contributions should appear. Please be sure to indicate whether the funders played any role in the study design, data collection and analysis, decision to publish, or preparation of the manuscript.

# Author comment: Author response to Decision Letter 1

**DOI:** 10.1371/journal.ppat.1014311.r003

16 Jun 2026

Attachment Submitted filename: Rebuttal_auresp_1.pdf

# Peer review: Decision Letter 1

**DOI:** 10.1371/journal.ppat.1014311.r004

**Authors:** Ronald Swanstrom, Peter Lidsky

6 Aug 2026

Vaccination accelerates the race of a respiratory virus against collective immunity

PLOS Pathogens

Dear Dr. Rouzine,

Thank you for submitting your manuscript to PLOS Pathogens. After careful consideration, we feel that it has merit but does not fully meet PLOS Pathogens's publication criteria as it currently stands. Therefore, we invite you to submit a revised version of the manuscript that addresses the points raised during the review process.

Please submit your revised manuscript by Oct 05 2026 11:59PM. If you will need more time than this to complete your revisions, please reply to this message or contact the journal office at plospathogens@plos.org. When you're ready to submit your revision, log on to https://www.editorialmanager.com/ppathogens/ and select the 'Submissions Needing Revision' folder to locate your manuscript file.

Please include the following items when submitting your revised manuscript:

* A letter that responds to each point raised by the editor and reviewer(s). You should upload this letter as a separate file labeled 'Response to Reviewers'. This file does not need to include responses to any formatting updates and technical items listed in the 'Journal Requirements' section below.

* A marked-up copy of your manuscript that highlights changes made to the original version. You should upload this as a separate file labeled 'Revised Manuscript with Track Changes'.

* An unmarked version of your revised paper without tracked changes. You should upload this as a separate file labeled 'Manuscript'.

We look forward to receiving your revised manuscript.

Kind regards,

Peter V. Lidsky, Ph.D.

Academic Editor

PLOS Pathogens

Ronald Swanstrom

Section Editor

Editor-in-Chief

PLOS Pathogens

orcid.org/0000-0003-2946-9497

Editor-in-Chief

PLOS Pathogens

orcid.org/0000-0002-7699-2064

**Additional Editor Comments (if provided):**

Dear Dr. Rouzine,

We got mixed feedback from the reviewers again. While all reviewers generally appreciate the importance of the paper, some serious criticisms are raised.

Reviewer #1, who also handled your manuscript during the last iteration, requested clarification on some questions that were not resolved previously. The paper's clarity still does not meet this reviewer's standards. Please address the issues raised by this reviewer, as clarity is of paramount importance, specifically in the case of conceptual papers such as yours.

Reviewer #2 raised additional concerns regarding the mathematical apparatus. Please address these comments as well.

With kind regards,

Peter

**Reviewers' Comments:**

Reviewer's Responses to Questions

**Part I - Summary**

Please use this section to discuss strengths/weaknesses of study, novelty/significance, general execution and scholarship.

Reviewer #1: The revision addresses several issues I raised but the manuscript remains very difficult to understand. I do think that this is a very interesting attempt to model the consequence of vaccination on the evolution of pathogens but I am expecting more than Fig 3 and 4 from theory. I mean, the analysis of a model should yield a better understanding of the process, not just a prediction. Perhaps the author has a good understanding of what is going on but, after reading the revision, I am still confused. I could probably spend more time on each step of the derivation but I feel the author should have tried harder to simplify key steps. Given the controversal nature of the claim, the argument has to be crystal clear and the manuscript is far from that. I list below some of the main issues that remain unresolved.

Reviewer #2: The manuscript presents an investigation of analytic models important problem of the evolution of a respiratory virus in a population driven by the collective immunity left by previous infections and vaccines.

My own area of expertise is in evolution modeling, including the infection spread in epidemics. I have read the text of the manuscript, the Rebuttal, and the referee reports.

In my view, the manuscript represents a fair compromise between a rigorous mathematical work and biological realism.

Mathematical derivations in the manuscript are solid (it is my area of expertise) and definitely more reliable than in the vast majority of contemporary biological articles on the topic.

Reviewers' critiques have been adequately addressed by the author.

Reviewer #3: The editors sent me a revised version of the paper, along with the reviewers' questions and the author's responses. Therefore, I had the opportunity to review the discussion, which I did very carefully. Regarding the possibility of publishing the paper, I believe it should be done.

Firstly, papers of this kind are, to some extent, aimed at bridging the gap between experimental biology, biological data processing, and purely mathematical modeling. Bridging these gaps is very difficult. In particular, I was very surprised to realize that the phenomenon of traveling waves in virology models is barely known, whereas the existence of such waves in models that are various modifications of the SIRV model with the addition of various effects, as well as the dependence of these waves' velocity on the reproduction number, is very well known in the modeling of biological processes and has a vast literature. Without claiming any generality, I would like to note reviews

Volpert, Petrovsky (2009, 2025)

doi:10.1016/j.plrev.2009.10.002

10.1016/j.plrev.2024.11.007

and a recent work

Barker, 2026

https://doi.org/10.1016/j.matcom.2025.04.027

with the references contained therein.

Secondly, the paper raises an interesting topic and contains conclusions that will undoubtedly spark discussion. The results of the work are intuitive and seem natural.

However, I have questions about the mathematical model the author uses to support his findings.

**********

**Part II – Major Issues: Key Experiments Required for Acceptance**

Please use this section to detail the key new experiments or modifications of existing experiments that should be absolutely required to validate study conclusions.

Generally, there should be no more than 3 such required experiments or major modifications for a "Major Revision" recommendation. If more than 3 experiments are necessary to validate the study conclusions, then you are encouraged to recommend "Reject".

Reviewer #1: The presentation of the model is very hard to follow. The model is complex because it mixes population dynamics and evolutionary dynamics. But other models have tried to model the interplay between these two dynamics and highlighted the balance between the influx of mutation (vaccination reduces this influx) and the strength of selection (vaccination increases it). What is new here is the attempt to study the evolutionary outcome when the mutation rate is NOT limiting. The author claims that (i) mutation is not limiting in Influenza and (ii) that when it is not limiting we have to account for intereference among mutations and therefore we need to use a dedicated theoretical framework for that.

Regarding (i): I specifically asked what was going on when the incidence drops and this assumption is not verified. In other words, it would be interesting to reconcile this formalism with previous attempts to model the effects of vaccination on the speed of adaptation. The S3 text does not really address that point. This is a pity because, again, the main value of theory is to clarify the connection with previous attempts to model similar problems. Otherwise, we are left with a collection of unrelated models that seem to compete against each other. I am genuinely expecting for more.

Regarding (ii): I specificially asked about the importance of multilocus genetics to understand the adaptation to vaccination because I want to understand its importance on this specific problem. The answer of the author was to recall that linkage is known to affect evolutionary dynamics since 1930... and then jumps to more recent theory accounting for the intereference between mutations and genetic drift. But I was asking for an explanation for the transition between the two regimes (with or without limiting mutation). This is perhaps a difficult question but the author chose not to address it. Instead, he seems to argue that it is irrelevant for influenza because of the high rates of mutation, the high nb of loci involved, the high incidence of the disease. Yet, when the vaccination coverage increases there must be a point where NinfUb<1 before the extinction threshold (as discussed by previous studies). Therefore, I think it is very important to acknowledge the different regimes of adaptation and to discuss the interface among them.

I made several comments that have not been taken into account: (i) the abstract claims that respiratory viruses persist in a population because of rates of immunity escape and does not mention the importance of waning immunity on the persistence of these viruses, (ii) the relevance of HIV and HCV is not clear because no vaccine against HIV and HCV.

The explanation of the two opposite effects of vaccination remains very unclear. For instance around line 126 or around 793. Both effects seem to be related to selection. Yet, the author makes a distinction between "transmission" and "selection". It seems to imply that transmission is not under selection... My point here is that the reader starts the Discussion with a confusing statement and, again, I am expecting from theoretical papers to help simplify a problem.

The model is complex (this is inevitable given the interplay between multiple effects) but there are many parts in its presentation that need longer developments or even figures to explain what is being done. For instance, how you combine natural and vaccine immunity or what is the difference (or the relation) between py and ez (by and bz).

Reviewer #2: (No Response)

Reviewer #3: I will not comment on the interpretation of the results, as I am not a virologist, and the qualifications of the respected reviewers and the author are sufficient to assess the quality of this interpretation.

I would like to discuss the model and the results of its mathematical analysis, about which I have serious questions.

The author belongs to a group that uses a modified model by Lin et al. [25]. This model appeared in [30]; it is quite simple and contains parameters that can be obtained from experimental data. However, I am quite confused by the analysis of this model.

1. The solution for the traveling wave type depends on the self-similar variable u = x-ct. This is done in both [25] and [30]. However, in this paper \bar x \neq ct, so the velocity is not constant.

2. P.7, Line 188: u=x-y. Is it different u?

3. Eq 3 states that R_e depends on time, the system variable. In fact, it depends on the time interval of vaccination t_v, so R_e=R_e(x) or R_e(x;t_v)

4. Now the most serious point.

I do not understand how solution (6) is obtained. It does not satisfy system (1). What is f (P.11, line 318)? Furthermore, for a traveling wave to exist, R_e must be R_e(u), but this is not the case.

The solution differs from the solution given in [30] and referred to by the author. In [30], R_0 is a constant, and the existence of a traveling wave is not in doubt.

Moreover, the results of the Monte Carlo simulation (Fig. 2) do not indicate that a delta function appears in the component i(u).

Furthermore, I do not understand where the explicit form of this solution is used.

If the author solves system (1}, then they need initial conditions. What are these conditions?

I have no way to verify the Monte Carlo simulation results, but I have no reason to distrust the author. Figure 2 clearly shows the existence of a wave moving at a constant speed (though I am confused by the note "for illustration only"). But since R_e is not constant, the picture must be different. What does the solution actually look like?

5. Is it possible to construct the dependence of the traveling wave velocity V on R_e with other parameters fixed? How is this velocity found? Is this formula s25, s26? They do not include R_e. Why are the notations for the velocity are different in s25, s26?

6. A traveling wave in model [25] exists only for R_e>1. The paper does not explicitly state this, and (3) does not imply that R_e cannot become less than 1. In this case the decrease of R_e destroys the traveling wave.

7. The cross immunity function K(u) in the capture of Fig.2 is different from K(u) given as (2), where it is called the normalized transmission function. Are they different?

8. Where in the model the selection s (\zeta in the Table 1) is used, the different forms of distribution of which are discussed in S2?

9. Are mutations taken into account in (1)?

P11, line 303: "First, consider dynamics of the existing strains and neglect mutation. Mutation will be

included later on".

What model are we talking about here?

As I understand from P9, line 255, mutation only occurs during the integration step. If this is the case, then we are solving a model different from (1).

10. The author constantly writes about memory effects. For example, "the vaccination events leave overlapping memory effects" (Fig 4)

I do not understand how the memory effects are included in the model, in contrast, for example, with

Liu, Sopasakis, 2024, https://www.sciencedirect.com/science/article/pii/S2405844024143071

Taylor, Carr, 2009 https://doi.org/10.1007/s00285-009-0256-9

Khalifi, Britton, 2023 https://doi.org/10.1098/rsif.2023.0042

11. It would be nice to see a comparison of the findings of this work with the results of other models, for example,

Wen et al. 2022,

https://doi.org/10.1086/717410

where it is claimed that "On average, more vaccination decreased the incidence of infection. Notably, this decrease was driven partly by a vaccine-induced decline in the rate of antigenic evolution."

12. Is the last equation (1) (conservation of the integral (i+r)) a consequence of the first two or is it an additional condition? If it is a consequence, then this must be proven. If this is an assumption, it may contradict the first two equations.

Further, in (1), the differential sign appears immediately after the integral in the first two equations, while in the last equation, it appears at the end. This should be written consistently.

In summary, I can say that I am ready to accept model (1), (3), but its mathematical and numerical analysis is unconvincing and raises many questions.

The mere fact of using a serious mathematical apparatus does not mean that this apparatus has been applied correctly.

**********

**Part III – Minor Issues: Editorial and Data Presentation Modifications**

Please use this section for editorial suggestions as well as relatively minor modifications of existing data that would enhance clarity.

Reviewer #1: Line 88: the virus do not mutate to avoid extinction or to avoid neutralization by antibodies! They mutate because replication is imperfect. This rate of mutation has evolutionary and epidemiological consequences... Reword to avoid the confusion.

Line 114 should be reworded

Reviewer #2: In principle, I share the concern the first referee that it would be better to consider multi–dimensional space. On the other hand, the 1D assumption allows to make the problem tractable by using simple integral–differential equations, as opposed to doing numeric simulation in multi–dimensional space.

I suggest the minor revisions:

1. Add a more detailed explanation regarding the validity of the one-dimensionality of antigenic space.

2. Change the title to the more concrete one, following to the recommendation of a third referee.

Reviewer #3: (No Response)

**********

PLOS authors have the option to publish the peer review history of their article (what does this mean?). If published, this will include your full peer review and any attached files.

If you choose “no”, your identity will remain anonymous but your review may still be made public.

**Do you want your identity to be public for this peer review?**  For information about this choice, including consent withdrawal, please see our Privacy Policy.

Reviewer #1: No

Reviewer #2: No

Reviewer #3: No

[NOTE: If reviewer comments were submitted as an attachment file, they will be attached to this email and accessible via the submission site. Please log into your account, locate the manuscript record, and check for the action link "View Attachments". If this link does not appear, there are no attachment files.]

**Figure resubmission:**

**Reproducibility:**

?>

# Author comment: Author response to Decision Letter 2

**DOI:** 10.1371/journal.ppat.1014311.r005

13 Aug 2026

Attachment Submitted filename: RebuttalRQ.pdf

# Peer review: Decision Letter 2

**DOI:** 10.1371/journal.ppat.1014311.r006

**Authors:** Ronald Swanstrom, Peter Lidsky

11 Sep 2026

Vaccination accelerates the race of influenza virus against collective immunity

PLOS Pathogens

Dear Dr. Rouzine,

Thank you for submitting your manuscript to PLOS Pathogens. After careful consideration, we feel that it has merit but does not fully meet PLOS Pathogens's publication criteria as it currently stands. Therefore, we invite you to submit a revised version of the manuscript that addresses the points raised during the review process. The paper may go back out for review.

Please submit your revised manuscript by Nov 10 2026 11:59PM. If you will need more time than this to complete your revisions, please reply to this message or contact the journal office at plospathogens@plos.org. When you're ready to submit your revision, log on to https://www.editorialmanager.com/ppathogens/ and select the 'Submissions Needing Revision' folder to locate your manuscript file.

Please include the following items when submitting your revised manuscript:

* A letter that responds to each point raised by the editor and reviewer(s). You should upload this letter as a separate file labeled 'Response to Reviewers'. This file does not need to include responses to any formatting updates and technical items listed in the 'Journal Requirements' section below.

* A marked-up copy of your manuscript that highlights changes made to the original version. You should upload this as a separate file labeled 'Revised Manuscript with Track Changes'.

* An unmarked version of your revised paper without tracked changes. You should upload this as a separate file labeled 'Manuscript'.

We look forward to receiving your revised manuscript.

Kind regards,

Peter V. Lidsky, Ph.D.

Academic Editor

PLOS Pathogens

Ronald Swanstrom

Section Editor

Editor-in-Chief

PLOS Pathogens

orcid.org/0000-0003-2946-9497

Dominique Soldati-Favre

Editor-in-Chief

PLOS Pathogens

orcid.org/0000-0003-4156-2109

**Journal Requirements:**

1. Your current Financial Disclosure states, 'I.M.R. worked within the framework of the state assignment of the Federal Agency for Scientific Organizations (FASO Russia: topic no. 075-00264-26-00). The funders had no role in study design, data collection and analysis, decision to publish, or preparation of the manuscript. I.M.R. received salary from his organization, IEPhB RAS, which is funded by the Federal Agency for Scientific Organizations.'. However, your funding information on the submission form does not indicate your name in the Grant Recipient column . Please indicate by return email the full and correct funding information for your study and confirm the order in which funding contributions should appear. Please be sure to indicate whether the funders played any role in the study design, data collection and analysis, decision to publish, or preparation of the manuscript.

2. Tables should be placed in the manuscript file immediately following the paragraph in which they are first mentioned. Please ensure your Tables are included in the correct locations in your manuscript file.

3. Some material included in your submission may be copyrighted. According to PLOS’s copyright policy, authors who use figures or other material (e.g., graphics, clipart, maps) from another author or copyright holder must demonstrate or obtain permission to publish this material under the Creative Commons Attribution 4.0 International (CC BY 4.0) License used by PLOS journals. Please closely review the details of PLOS’s copyright requirements here: PLOS Licenses and Copyright. If you need to request permissions from a copyright holder, you may use PLOS's Copyright Content Permission form.

Please respond directly to this email and provide any known details concerning your material's license terms and permissions required for reuse, even if you have not yet obtained copyright permissions or are unsure of your material's copyright compatibility. Once you have responded and addressed all other outstanding technical requirements, you may resubmit your manuscript within Editorial Manager.

Potential Copyright Issues:

Figure 1 : Please confirm whether you drew the images / clip-art within the figure panels by hand. If you did not draw the images, please provide (a) a link to the source of the images or icons and their license / terms of use; or (b) written permission from the copyright holder to publish the images or icons under our CC BY 4.0 license. Alternatively, you may replace the images with open source alternatives. See these open source resources you may use to replace images / clip-art:

- https://commons.wikimedia.org

- https://openclipart.org/

If the reviewer comments include a recommendation to cite specific previously published works, please review and evaluate these publications to determine whether they are relevant and should be cited. There is no requirement to cite these works unless the editor has indicated otherwise.

[Note: HTML markup is below. Please do not edit.]

**Reviewers' Comments:**

Reviewer's Responses to Questions

**Part I - Summary**

Please use this section to discuss strengths/weaknesses of study, novelty/significance, general execution and scholarship.

Reviewer #2: In the revised version the author changed the text following t my comments.

I strongly support the acceptance of the article, from the perspectives of evolution biology.

As I mentioned already, the method is unique in quantitative biology- looking both rigorous methods and realistic model,

a good compromise, very valuable.

Even if the author did not compare his method with the all alternative methods, the article is valuable for the sciene.

Reviewer #3: Some points have been clarified, but the mathematical model contains several important flaws. Since the main conclusions are drawn from the simulation, these cannot be dismissed.

1. It is claimed that the conservation of the integral from (i + r) is a consequence of the first two equations in (1). This is incorrect. This would be true if the kernel were symmetric (K(u)=K(-u)), but kernel (2) is asymmetric. This problem does not exist in paper [57], to which the author constantly refers. There, the integral in nonlocal terms is taken over the entire axis, and the integral of the sum of the components is indeed conserved. This problem also does not exist in paper [30] due to the special choice of the kernel (K(u)=0, u>0).

2. It is claimed that the form of the traveling wave is independent of the initial data. This is also incorrect. In such models, both the possibility of forming a traveling wave and its velocity depend on the choice of initial data. This issue is discussed in detail in paper [57], and it is clearly stated that

"Simulations were initialized from pre-generated steady-state traveling waves, equilibrated under each

parameter set to avoid transient effects (Supplement 1.1)." (Beginning of Sec.2.7)

Of course, I cannot demand a rigorous mathematical justification and stability of the traveling wave, but now I have doubts about the very model that underlies the calculations.

3. Regarding the formulation of the model: it should be clearly stated that it consists of the first two equations (1), and the third equation should not be included in the model; it should be a consequence of the first two (but in its current form, it is not a consequence). Throughout the text, dx should be either before or after the integrand, consistently. Preferably after.

4. I do not understand formula (8). It should be derived from (5), which is an analogue of (7) from [30]. But then, apparently, the integral should be taken over the entire axis. Further, the derivative is taken incorrectly in any case.

I believe all stages of the calculations should be presented in detail, in the spirit of [57]. Otherwise, the reasoning is unconvincing to the reader.

The paper requires major revision.

**********

**Part II – Major Issues: Key Experiments Required for Acceptance**

Please use this section to detail the key new experiments or modifications of existing experiments that should be absolutely required to validate study conclusions.

Generally, there should be no more than 3 such required experiments or major modifications for a "Major Revision" recommendation. If more than 3 experiments are necessary to validate the study conclusions, then you are encouraged to recommend "Reject".

Reviewer #2: (No Response)

Reviewer #3: (No Response)

**********

**Part III – Minor Issues: Editorial and Data Presentation Modifications**

Please use this section for editorial suggestions as well as relatively minor modifications of existing data that would enhance clarity.

Reviewer #2: (No Response)

Reviewer #3: (No Response)

**********

PLOS authors have the option to publish the peer review history of their article (what does this mean?). If published, this will include your full peer review and any attached files.

If you choose “no”, your identity will remain anonymous but your review may still be made public.

**Do you want your identity to be public for this peer review?**  For information about this choice, including consent withdrawal, please see our Privacy Policy.

Reviewer #2: No

Reviewer #3: No

[NOTE: If reviewer comments were submitted as an attachment file, they will be attached to this email and accessible via the submission site. Please log into your account, locate the manuscript record, and check for the action link "View Attachments". If this link does not appear, there are no attachment files.]

**Figure resubmission:**

**Reproducibility:**

?>

# Author comment: Author response to Decision Letter 3

**DOI:** 10.1371/journal.ppat.1014311.r007

16 Sep 2026

Attachment Submitted filename: Response to Reviewer 3.pdf

# Peer review: Decision Letter 3

**DOI:** 10.1371/journal.ppat.1014311.r008

**Authors:** Ronald Swanstrom, Peter Lidsky

29 Sep 2026

Dear Dr. Rouzine,

We are pleased to inform that your manuscript, "Vaccination accelerates the race of influenza virus against collective immunity", has been editorially accepted for publication at PLOS Pathogens.

Before your manuscript can be formally accepted and sent to production, you will need to complete our formatting changes, which you will receive by email within a week. Please note that your manuscript will not be scheduled for publication until you have made the required changes.

IMPORTANT NOTES

(1) Copyediting and Proofreading: The corresponding author will receive a typeset proof for review, to ensure errors have not been introduced during production. Please review the PDF proof of your manuscript carefully, as this is the last chance to correct any errors. Please note that major changes, or those which affect the scientific understanding of the work, will likely cause delays to the publication date of your manuscript.

(2) Appropriate Figure Files: Please remove all name and figure # text from your figure files. Please also take this time to check that your figures are of high resolution, which will improve the readbility of your figures and help expedite your manuscript's publication. Please note that figures must have been originally created at 300dpi or higher. Do not manually increase the resolution of your files. For instructions on how to properly obtain high quality images, please review our Figure Guidelines, with examples at: http://journals.plos.org/plospathogens/s/figures.

We encourage you to use PACE (http://pace.apexcovantage.com/), a figure-checking tool which helps authors ensure all figures meet PLOS requirements so that figures can publish in the highest quality. PACE is a digital diagnostic and conversion tool for figure files. It will provide information about any failed check(s) and, if able, will automatically convert the figure into an acceptable file that passes quality checks.

To use PACE, you must first register as a user. Then, login and navigate to the UPLOAD tab, where you will find detailed instructions on how to use the tool.

(3) Striking Image: Please upload a striking still image to accompany your article if one is available (you can include a new image or an existing one from within your manuscript). Should your paper be accepted, this image will be considered for our monthly issue image and may also appear on our website to feature your article. Please upload this as a separate file, selecting "striking image" as the file type upon upload. Please also include a separate "Other" file with a caption, including credits and any potential copyright information. Please do not include the caption in the main article file. If your image is from someone other than yourself, please ensure that the artist has read and agreed to the terms and conditions of the Creative Commons Attribution License at http://journals.plos.org/plospathogens/s/content-license. Please note that PLOS cannot publish copyrighted images.

(4) Press Release or Related Media: If you or your institution will be preparing press materials for this manuscript, please inform our press team in advance at plospathogens@plos.org as soon as possible. We ask that you contact us within one week to plan ahead of our fast Production schedule. If you need to know your paper's publication date for related media purposes, you must coordinate with our press team, and your manuscript will remain under a strict press embargo until the publication date and time.

(5) Update your Profile Information: Now that your manuscript has been provisionally accepted, please log into Editorial Manager and update your profile, if needed. Go to http://www.editorialmanager.com/ppathogens, log in, and click on the "Update My Information" link at the top of the page. Please update your user information to ensure an efficient production and billing process.

(6) LaTeX users only: Our staff will ask you to upload a TEX file in addition to the PDF before the paper can be sent to typesetting, so please carefully review our Latex Guidelines http://journals.plos.org/plospathogens/s/latex in the meantime.

Thank you again for supporting Open Access publishing; we are looking forward to publishing your work in PLOS Pathogens.

Best regards,

Peter V. Lidsky, Ph.D.

Academic Editor

PLOS Pathogens

Ronald Swanstrom

Section Editor

PLOS Pathogens

Dominique Soldati-Favre

Editor-in-Chief

PLOS Pathogens

***********************************************************

Reviewer's Responses to Questions

**Part I - Summary**

Please use this section to discuss strengths/weaknesses of study, novelty/significance, general execution and scholarship.<br/><br/>

Reviewer #3: I am satisfied with the author's responses and believe that the current version of the article can be published.

--------------------

**Part II – Major Issues: Key Experiments Required for Acceptance**

Please use this section to detail the key new experiments or modifications of existing experiments that should be absolutely required to validate study conclusions.<br/><br/>Generally, there should be no more than 3 such required experiments or major modifications for a "Major Revision" recommendation. If more than 3 experiments are necessary to validate the study conclusions, then you are encouraged to recommend "Reject".<br/><br/>

Reviewer #3: (No Response)

--------------------

**Part III – Minor Issues: Editorial and Data Presentation Modifications**

Please use this section for editorial suggestions as well as relatively minor modifications of existing data that would enhance clarity.<br/><br/>

Reviewer #3: (No Response)

--------------------

PLOS authors have the option to publish the peer review history of their article (what does this mean?). If published, this will include your full peer review and any attached files.

If you choose “no”, your identity will remain anonymous but your review may still be made public.

**Do you want your identity to be public for this peer review?**  For information about this choice, including consent withdrawal, please see our Privacy Policy.

Reviewer #3: No

# Editor report: Acceptance letter

**DOI:** 10.1371/journal.ppat.1014311.r009

**Authors:** Ronald Swanstrom, Peter Lidsky

Dear Dr. Rouzine,

We are delighted to inform you that your manuscript, "Vaccination accelerates the race of influenza virus against collective immunity," has been formally accepted for publication in PLOS Pathogens.

We have now passed your article onto the PLOS Production Department who will complete the rest of the pre-publication process. All authors will receive a confirmation email upon publication.

Thank you again for supporting open-access publishing; we are looking forward to publishing your work in PLOS Pathogens.

Best regards,

Dominique Soldati-Favre

Editor-in-Chief

PLOS Pathogens
